# The burden of anxiety, depression, and substance use disorders attributable to childhood maltreatment among adolescents in Africa: insights from the global burden of disease study 2021

**DOI:** 10.1186/s12991-025-00611-8

**Published:** 2025-11-18

**Authors:** Louisa Esi Mackay, Blen Dereje Shiferaw, Yunjiao Luo, Na Yan, Yuhao Wang, Yingxue Wang, Yihan Wang, Xinyi Hu, SuSu Tian, Jiayi Tian, Huihang Lan, Yuxuan Liu, Wei Wang

**Affiliations:** 1 School of Public Health, Xuzhou Medical University, 209 Tong Shan Road, Xuzhou, 221004 Jiangsu China; 2 Department of Public Health, The Affiliated Xuzhou Oriental Hospital of Xuzhou Medical University, Xuzhou, Jiangsu China; 3 Research Center for Psychological Crisis Prevention and Intervention of College Students in Jiangsu Province, Xuzhou Medical University, Xuzhou, China; 4 Jiangsu Engineering Research Center of Biological Data Mining and Healthcare Transformation, Xuzhou Medical University, Xuzhou, 221004 Jiangsu China

**Keywords:** Anxiety, Depression, Substance use disorder, Childhood maltreatment, Adolescents

## Abstract

**Background:**

Childhood maltreatment is a major risk factor for mental and substance use disorders particularly among adolescents however, there’s no comprehensive report of its attributable burden on anxiety, depression, and substance use disorder in Africa. This research sort to delineate the Disability-adjusted life years (DALYs) trend and age-period-cohort effects on these disorders attributable to childhood maltreatment among 10–24-year-olds in Africa.

**Methods:**

DALY estimates and 95% uncertainty intervals for anxiety, depression, and substance use disorder attributable to childhood maltreatment among adolescents in Africa were extracted from the Global Burden of Disease Study (GBD) 2021. Trends between 1990 and 2021 were analyzed using Joinpoint regression analysis. The Age-Period-Cohort model was used to determine the effect of age, period, and cohort on these disorders.

**Results:**

In 2021, the DALY rate for anxiety was 95.41 (37.32–185.13), 117.57 (54.31–209.16) for depression, and 3.82 (0.63–10.38) for substance use disorder per 100,000 population among adolescents in Africa. Trend analysis showed an increase in anxiety [AAPC: 0.88 (0.84–0.91)] and depressive disorders [AAPC: 0.71(0.68–0.74)]. Niger recorded the highest growth in average annual percentage change (AAPC): 3.17 [95% CI 3.08–3.27] for anxiety, Burkina Faso recorded the highest rise in AAPC: 1.96 [95% CI 1.85–2.08] for depression, and Uganda recorded the highest incline in AAPC: 0.65 [95% CI 0.59–0.72] for substance use disorder. Higher age effects were noted in all disorders, particularly in the 15–19-year group. Period effects were higher for anxiety and depressive disorders and lower for substance use disorders. A higher cohort effect was observed in all disorders during the study period.

**Conclusions:**

Anxiety and depressive disorders due to childhood maltreatment have been on the rise for 3 decades in Africa. Given the implications of the early onset and lifetime burden of mental and substance use disorders, a comprehensive policy framework that facilitates increased access to specialists in childhood maltreatment-related mental disorder services is essential.

**Supplementary Information:**

The online version contains supplementary material available at 10.1186/s12991-025-00611-8.

## Background

Childhood maltreatment encompassing physical abuse, sexual abuse, or negligence has emerged as a critical public health concern with far-reaching consequences that often persist into adulthood [1]. Yearly, about 4–16% of children are physically abused, while one in ten children is psychologically abused [2]. Recent global health data has highlighted the severity of this issue. Among 88 risk factors for diseases in 2021, bullying victimization and childhood sexual abuse ranked 7th and 19th respectively [3]. Alarmingly, bullying victimization saw a 12.4% increase between 2000 and 2021 among children aged 5–14 years, while a 12.9% rise was observed in the 15–49 age group over the same period [3]. Reports from Africa have not been different. About 48.4% of young adults in South Africa have experienced some form of childhood maltreatment [4] while the rates of sexual, physical, and emotional abuse ranged from 14.5% to 89.4% of teenagers in Burundi [5]. The long-term effects of childhood maltreatment on the development of behavioral and psychiatric disorders later in life, such as non-suicidal self-injury, post-traumatic stress disorder (PTSD), depression, substance use, and anxiety disorders [1, 6–8] draws further attention to its detrimental effects.

In 2019, the global number of disability-adjusted life years (DALYs) due to mental disorders increased from 80.8 million (95% uncertainty interval [UI] 59.5–105.9) in 1990 to 125.3 million (93.0–163.2) [9]. Of these, depressive and anxiety disorders accounted for the largest proportion, recording 37.3% and 22.9% respectively. In Africa, the prevalence of depression and anxiety among adolescents was 26.9% and 29.8% respectively [10] while substance use disorder is on the rise [11]. It is known that approximately 40% of all mental health disorders are associated with childhood maltreatment [12]. The increasing burden of depression and anxiety disorders further highlights the long-term effects of childhood maltreatment. Substance use disorders, on the other hand, showed a marginal decrease of 0.1% [13], and accounted for 32.5 million (95% UI 26.8–38.8) DALYs of which alcohol use disorders made up 52.1% (47.3–56.6) [14]. Although there was a slight decrease, the number was still alarming.

Despite these trends, many existing studies have focused on high-income countries such as Europe [15], the United Kingdom [16], and Asia [17] leaving low to middle-income countries largely unexamined. Moreover, the age between 10 and 24 years is the formative and transitioning years of adulthood [18] and the peak time for the onset of mental disorders and substance use disorders [4]. Given these factors, there is an urgent need for comprehensive research to fully understand the scope and impact of childhood maltreatment on adolescents in Africa.

This study aimed to quantify the burden of anxiety, depression, and substance use disorder attributed to specific forms of childhood maltreatment, namely childhood sexual abuse and bullying victimization, across 52 African countries using the most recent available data from the Global Burden of Disease (GBD) study. Our analysis utilized Joinpoint regression to examine trends in the number and age-standardized rates of DALYs associated with these conditions. Furthermore, we conducted an Age, Period, and Cohort analysis to elucidate the effects of age, period, and cohort on depression, anxiety, and substance use disorders attributed to childhood maltreatment.

## Methods

### Data sources

The Global Burden of Diseases, Injuries, and Risk Factors Study (GBD) 2021 estimates exposure levels, relative health risks, and attributable disease burden for 88 risk factors in 204 countries and territories and 811 subnational locations, from 1990 to 2021 [3]. Data from randomized controlled trials, cohort studies, case–control studies, and meta-analyses that reported relative risks (RR) of mortality or morbidity from a given health outcome as a function of risk exposure were compiled and analyzed to generate relative risk estimates for risk-outcome pairs. Risk-specific estimates of summary exposure value (SEV), relative risk (RR), population attributable fraction (PAF), risk attributable burden measured in disability-adjusted life years (DALYs; the sum of years of life lost to premature mortality and years lived with disability), and deaths were provided by GBD [19]. Detailed information on the data sources used for risk factor estimation in the GBD 2021 is also available (Additional File Appendix C, https://ghdx.healthdata.org/gbd-2021/sources).

DALY estimates for GBD 2021 were informed by 100,983 data sources (including 19,189 sources newly used in 2021) [13]. To calculate DALYs, YLDs, and YLLs were summed according to location, age, sex, year, and cause. The 95% uncertainty interval (UI) is defined by the 25th and 975th values of the ordered 1000 estimates based on the GBD algorithm [20]. Further details of the general methodologies for GBD have been described elsewhere [3]. Data and the protocol for GBD can be accessed through the Global Health Data Exchange Results Tool (Additional File Appendix B, http://ghdx.healthdata.org/gbd-results-tool).

GBD also grouped countries according to the socio-demographic index (SDI), a composite measure of general socioeconomic development status [20]. The SDI was computed using the total fertility rate of females under 25 years of age, lag-distributed income per person, and educational attainment of those who were 15 years of age or older. Higher SDI values, which ranged from 0 to 1, indicated greater development. Countries were categorized into five groups based on the SDI quintiles: low, low-middle, middle, high-middle, and high SDI.

For this study, the DALYs number and rate of depression, anxiety, and substance use disorders attributable to childhood maltreatment for the 10–24 age group (males, females, and both sexes combined), and 52 African countries were directly extracted from GBD 2021 at the disease surveillance system on the Global Health Data Exchange website (http://ghdx.healthdata.org). Although substance use disorders are classified separately from mental disorders in the GBD hierarchy, the International Classification of Diseases (ICD) classifies substance use disorders as mental disorders; therefore, in this study, the DALYs of substance use disorders, specifically alcohol use, were also estimated [21]. This study complied with the guidelines for accurate transparent reporting of health estimates [22].

### Data analysis

#### Joinpoint regression analysis

Firstly, this study aimed to report the trends in anxiety, depression, and substance use disorders attributable to childhood maltreatment in 52 African countries. The Joinpoint regression program version 5.2.0 (Statistical Research and Applications Branch, National Cancer Institute) was used to identify the changing DALYs trend of childhood maltreatment attributable to anxiety, depression, and substance use disorder among adolescents and young adults aged 10–24.

Joinpoint regression analysis is a statistical tool that analyzes temporal patterns [23]. The model’s fundamental principle is to find the inflection point and then divide the long-term epidemiological trend of a disease into multiple segments (up to six segments maximum) based on the minimum and maximum number of join points selected [24]. A straight line is then fitted to each segment to describe the epidemiological features of the disease over a specific period. For each statistically significant segment, the Annual Percent Change (APC), which quantifies the rate of change between two connecting points, is calculated [25]. The Average Annual Percentage Change (AAPC), which describes the overall rate of change, is also calculated [26]. The Z-test determines whether AAPC or APC is different from 0. If APC/AAPC > 0, then the trend is upward. If APC/AAPC < 0, then the trend is downward.

#### Age-period-cohort analysis

Secondly, we aimed to report the effects of age, period, and cohort on anxiety, depression, and substance use disorder attributable to childhood maltreatment in 52 African countries. The age-period-cohort model is frequently regarded as an advanced method that transcends conventional analysis in the social and health sciences, with an emphasis on evaluating the influence of medical technologies and social factors related to natural history, contemporary time, health practices, and early life social exposure on disease trends [27]. It provides valuable insights into the impact of aging processes and external factors on disease prevalence by breaking down the disease epidemic trend into three components: age, period, and cohort effects.

The age effect refers to the influence of physiological and social processes on an individual’s age [28]. Period effects are changes with time that affect each age group simultaneously and may be due to changes in the social, cultural, economic, or natural environment [29]. Cohort effects are defined as the unique experiences of social events, historical shifts during formative ages, and the changes between groups of persons born in the same year [28, 30–32]. In this study, the web-based age, period, and cohort model (https://analysistools.cancer.gov/apc/) was used to analyze the effects of age, period, and cohort on anxiety, depression, and substance use disorders attributable to childhood maltreatment in adolescents and young adults.

For this model, age and period intervals must be equal; therefore, ages were divided into 5-year age groups (10–14 years, 15–19 years, and 20–24 years), and periods were divided into 5-year groups (1992–1996, 1997–2001, …, 2017–2021). Finally, eight partially coincidental birth cohorts were generated using age and time groups. In addition, for this model, the longitudinal representation of the age effect involves fitting a set number of birth cohorts, with adjustments made for period bias to a specific age rate. The period/cohort effect is depicted by the period/cohort relative DALYs, calculated as the ratio of the age-specific DALY rate in each period/cohort relative to the reference period/cohort. The reference age for this study was 10 years and 1992 was the reference period. This choice did not influence the interpretation of results [33]. The statistical significance of these parameters was tested using the Wald χ^2^ test, with a significance level of 0.05. These analyses were performed using R version 4.2.1.

## Results

### Joinpoint regression analysis of DALYs of anxiety, depression, and substance use disorders attributable to childhood maltreatment

#### Trends in anxiety, depression, and substance use disorders in Africa

**Fig. 1:**
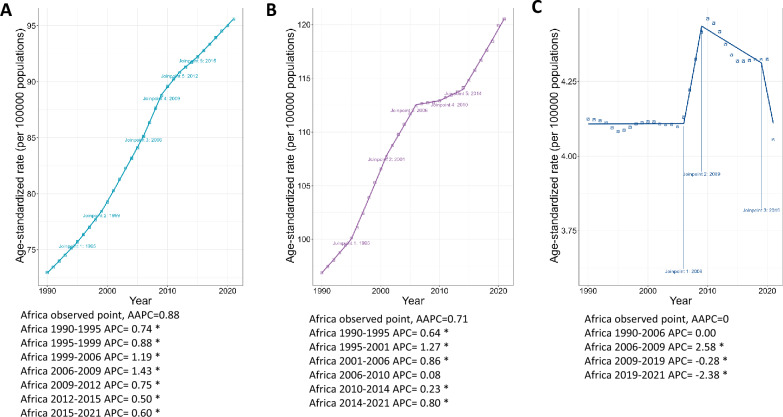
Joinpoint regression of DALY rate of anxiety (**a**), depression (**b**), and substance use (**c**) disorders and their AAPC from 1990 to 2021. The joinpoints indicate a turning point demarking significance. *Indicates p < 0.05. *AAPC* average annual percentage change, *APC* Annual percentage change

In 2021, 421,302 cases of anxiety disorder, 519,184 (239,833–923,623) cases of depressive disorder, and 16,852 (2771–45,857) cases of substance use disorder attributable to childhood maltreatment were recorded among adolescents in Africa (Table 1). Males recorded higher cases and Daly rates for anxiety and substance use disorders, whereas females recorded higher cases and DALY rates for depressive disorders. Among the age groups, the 15–19-year-olds had higher rates of anxiety and depressive disorders while the 20–24-year-olds had higher DALY rates for substance use disorder (Table 1). The trend analysis revealed an increasing average annual percentage (AAPC) for anxiety (0.88 [0.84–0.91]) and depression (0.71[0.68–0.74]) and a stable AAPC for substance use disorder [0(− 0.13–0.13)] (Fig. 1).

**Table 1:** Number of cases, DALYs rate, and AAPC of anxiety, depression, and substance use between 1990 and 2021

	Case(n) 1990	DALYs rate 1990 (per 100,000)	Case(n) 2021	DALYs rate 2021(per 100,000)	AAPC	P-Value
**Anxiety disorders**						
Africa	146,508 (52,513–298,584)	72.9 (26.13–148.56)	421,302 (164,797–817,479)	95.41 (37.32–185.13)	0.88 (0.84–0.91)	< 0.00
**Sex**						
Male	85,770 (33,394–164,207)	86.48 (33.67–165.56)	227,378 (91,956–421,570)	103.45 (41.84–191.81)	1.25 (1.23–1.26)	< 0.00
Female	60,738 (18,132–135,740)	59.66 (17.81–133.33)	193,924 (63,138–410,293)	87.43 (28.47–184.99)	0.58 (0.55–0.62)	< 0.00
**Age group**						
10–14 years	53,184 (19,200–110,012)	67.58 (24.4–139.8)	146,069 (57,390–290,745)	86.35 (33.93–171.87)	0.79 (0.76–0.83)	< 0.00
15–19 years	54,284 (19,148–112,032)	81.93 (28.9–169.08)	156,853 (60,027–311,770)	106.51 (40.76–211.71)	0.85 (0.81–0.89)	< 0.00
20–24 years	39,040 (12,118–84,464)	69.67 (21.63–150.74)	118,380 (39,646–249,662)	94.59 (31.68–199.49)	0.99 (0.98–1.01)	< 0.00
**Depressive disorders**						
Africa	188,407 (83,231–343,268)	93.74 (41.41–170.79)	519,184 (239,833–923,623)	117.57 (54.31–209.16)	0.71 (0.68–0.74)	< 0.00
**Sex**						
Male	97,425 (42,604–179,255)	98.23 (42.96–180.74)	254,226 (115,024–467,278)	115.67 (52.33–212.61)	0.93 (0.9–0.95)	< 0.00
Female	90,982 (40,714–169,200)	89.37 (39.99–166.2)	264,958 (124,933–478,011)	119.46 (56.33–215.52)	0.5 (0.46–0.54)	< 0.00
**Age group**						
10—14 years	34,334 (14,108–65,267)	43.63 (17.93–82.94)	93,836 (39,629–170,572)	55.47 (23.43–100.83)	0.79 (0.71–0.86)	< 0.00
15–19 years	76,044 (32,366–141,249)	114.77 (48.85–213.18)	210,472 (94,631–379,941)	142.92 (64.26–258)	0.71 (0.68–0.74)	< 0.00
20–24 years	78,029 (33,579–152,228)	139.26 (59.93–271.68)	214,877 (95,138–411,654)	171.69 (76.02–328.93)	0.68 (0.66–0.69)	< 0.00
**Substance use disorders**						
Africa	7679 (1236–20,350)	3.82 (0.61–10.13)	16,852 (2771–45,857)	3.82 (0.63–10.38)	0 (− 0.13–0.13)	< 0.00
**Sex**						
Male	4773 (763–12,533)	4.81 (0.77–12.64)	9852 (1644–27,608)	4.48 (0.75–12.56)	0.29 (0.25–0.32)	< 0.00
Female	2906 (457–7744)	2.85 (0.45–7.61)	6999 (1123–18,345)	3.16 (0.51–8.27)	− 0.22 (− 0.34 to − 0.11)	< 0.00
**Age group**						
10—14 years	197 (29–547)	0.25 (0.04–0.7)	433 (53–1251)	0.26 (0.03–0.74)	0.11 (0.06–0.16)	< 0.00
15–19 years	2108 (333–5759)	3.18 (0.5–8.69)	4858 (841–12,687)	3.3 (0.57–8.61)	0.16 (0.08–0.25)	< 0.00
20–24 years	5375 (847–14,202)	9.59 (1.51–25.35)	4858 (841–12,687)	9.24 (1.4–25.06)	− 0.06 (− 0.22–0.09)	0.42

*DALYs* disability-adjusted life years, *AAPC* average annual percentage change

#### National trends in anxiety, depressive, and substance use disorders

Among the 52 African countries, Egypt recorded the highest number of cases, 76,537 (34,457–137,090 95% UI) in 2021 (Additional file Table 1). Niger (AAPC: 3.17 [3.08–3.27]), Burkina Faso (AAPC: 3.06 [2.98–3.14]), Burundi (AAPC: 2.45 [2.39–2.5]), Djibouti (AAPC: 2.41 [2.32–2.51]), and Guinea (AAPC: 2.33 [2.27–2.39]) recorded the top five highest AAPC (Additional file Fig S3 A) while Lesotho (AAPC: 0.36 [0.31–0.41]), Gabon (AAPC: 0.36 [0.32–0.4]), Zimbabwe (AAPC: 0.3 [0.27–0.33]), South Africa (AAPC: 0.3 [0.27–0.33]), and Congo (AAPC: 0.29 [0.27–0.32]) recorded the least increase (Additional file Fig. 2a).

**Fig. 2:**
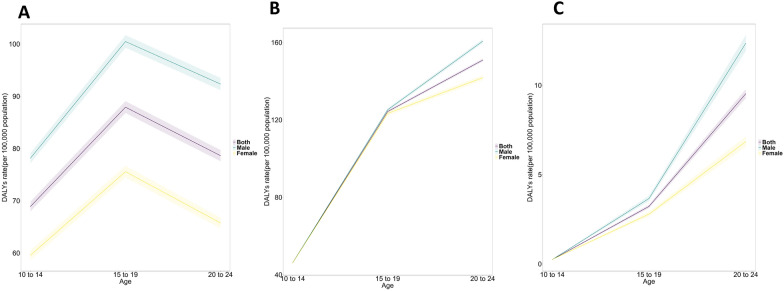
Age effects on anxiety (**a**), depression (**b**), and substance use (**c**) disorders attributable to childhood maltreatment in Africa. The age effect on DALY of anxiety is depicted through the longitudinal rates specific to age, adjusted for variations across different birth cohorts, considering the period-specific deviations. The shaded areas denote the corresponding 95% CIs. *DALY* Disability-adjusted life years

Egypt also recorded the highest number of cases of depression, 67,948 (34,947–118,505 95% UI) in 2021 (Additional file Table 2). In the trend analysis, a similar increasing trend in anxiety was observed in depressive disorders. All 52 African countries showed an increasing trend except for Congo (AAPC: − 0.06 [− 0.11 to − 0.01]) (Additional file Fig. 2B). Among the remaining 51 nations with a rising trend, Burkina Faso (AAPC: 1.96 [1.85–2.08]), Niger (AAPC: 1.9 [1.85–1.96]), Guinea (AAPC: 1.77 [1.72–1.83]), Djibouti (AAPC: 1.76 [1.68–1.84]) and Somalia (AAPC: 1.58 [1.51–1.64]) recorded the highest increase (Additional file Fig S3 b).

Nigeria recorded the highest number of cases [3330 (580–7607 95% UI)] of substance use disorder (Additional file Table 3). The trends declined in some countries and increased in others. Eswatini (AAPC: − 1.23 [− 1.35 to − 1.11]), South Africa (AAPC: − 0.91 [− 1.2 to − 0.62]), Kenya (AAPC: − 0.57 [− 0.6 to − 0.55]), Sudan (AAPC: − 0.4 [− 0.46 to − 0.34]) and Burundi (AAPC: − 0.36 [− 0.49 to − 0.24]) recorded the fastest decline (Additional file Fig. 2C). Uganda (AAPC: 0.65 [0.59–0.72]), Lesotho (AAPC: 0.22 [0.16–0.28]), Ethiopia (AAPC: 0.22 [0.12–0.31]), Zambia (AAPC: 0.17 [0.1–0.23]) and Chad (AAPC: 0.16 [0.06–0.26]) showed the highest increase in AAPC (Additional file Fig S3 c). Trends in Uganda showed dramatic changes between 1999 and 2007 (APC: 1.08 [0.94–1.21]) and between 2007 and 2017 (APC: 0.19 [0.1–0.28]) (Additional file Fig. 3c).

**Fig. 3:**
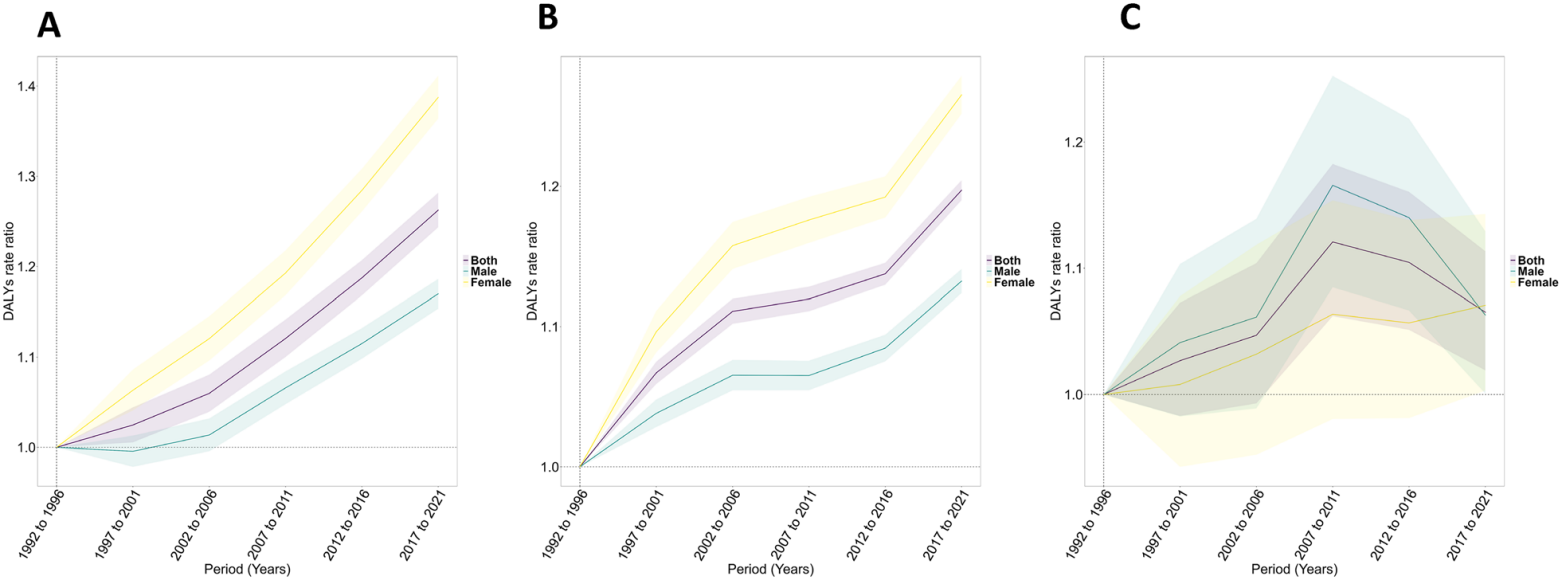
Period effects on anxiety (**a**), depression (**b**), and substance use (**c**) disorders attributable to childhood maltreatment in Africa. Period effects are shown through the relative risk of DALYs of anxiety during different periods, calculated as the ratio of the age-specific rates from 1992–1996 to 2017–2021, with the reference period set as 1992–1996. The shaded areas denote the corresponding 95% CIs. *DALY* Disability-adjusted life years

### Age, period, and cohort effects

#### Age effects

Figure 2 graphically presents the longitudinal age curves of sex-specific anxiety, depression, and substance use disorders attributable to childhood maltreatment in Africa. Generally, the DALY rates of anxiety, depression, and substance use disorder increased prominently with age in both sexes. However, after reaching the highest peak at 15–19 years for anxiety, a sharp decline was observed (Fig. 2a). On the other hand, depression and substance use disorders continued to rise for 24 years (Fig. 2b, c). For all disorders and ages, males showed higher age risks than females.

#### Period effects

Figure 3 shows the period curves for sex-specific anxiety, depression, and substance use disorders attributable to childhood maltreatment in Africa. An increasing trend was observed over time for anxiety and depression (Fig. 3a, b). Substance use disorder on the other hand showed an inverted ‘V’ shape increasing between 2002 and 2011 and decreasing from 2012 to 2021 (Fig. 3c). In contrast to the age effects, females recorded higher DALY rates for anxiety and depressive disorders over the years, while males only recorded higher values for substance use disorders in period effects.

#### Birth effects

**Fig. 4:**
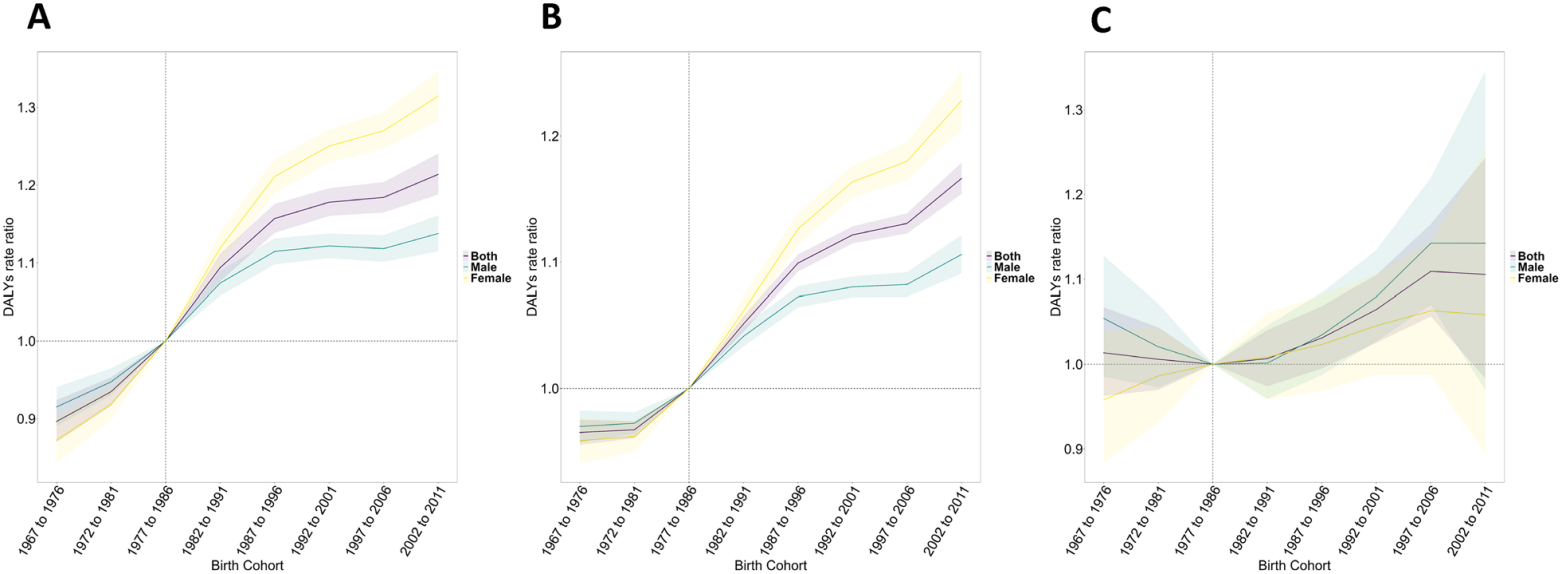
Cohort effects on anxiety (**a**), depression (**b**), and substance use (**c**) disorders attributable to childhood maltreatment in Africa. Birth cohort effects are demonstrated by the cohort relative risk of DALYs and calculated as the ratio of age-specific rates from the 1967–1976 cohort to the 2002–2011 cohort, with the reference cohort set at 1977–1986. The shaded areas denote the corresponding 95% CIs. *DALY* Disability-adjusted life years

Figure 4 depicts the birth cohort curves of sex-specific anxiety, depression, and substance use disorders attributable to childhood maltreatment in Africa. The relative cohort risk increased in the subsequent birth cohorts for all disorders. Similar to the period effects, females recorded higher DALY rates for anxiety and depressive disorders (Fig. 4a, b), while males recorded higher rates for substance use disorders (Fig. 4c).

### Associations with the SDI

**Fig. 5:**
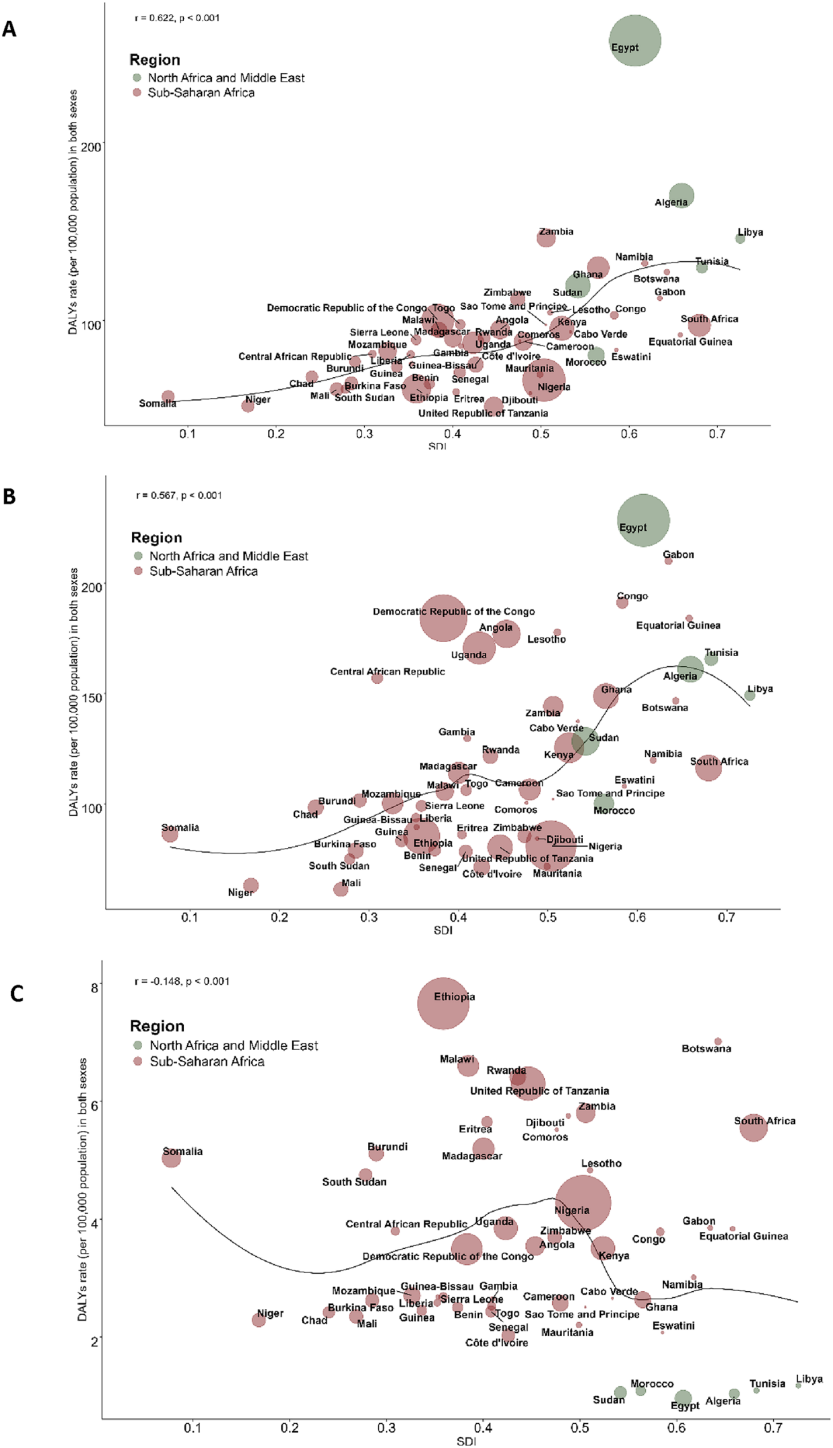
Trends in DALYs rate for anxiety (**a**), depression (**b**), and substance use (**c**) disorders in Africa by SDI in 2021. SDI: Socio-demographic Index; the Black line represents the expected mortality based on SDIs in all countries

Figure 5 shows the observed versus expected DALYs attributable to anxiety, depression, and substance use disorders based on the SDI at the national level. Anxiety disorders showed a rising trend according to the SDI (Fig. 5a), with the highest DALYs rate recorded in Egypt, a low-middle SDI country. A similar trend was observed for depressive disorders however, low-middle SDI countries had similar DALY rates to those of the middle and high-middle SDI (Fig. 5b). For substance use disorders, low SDI countries had higher observed DALY rates than middle and high-middle SDI countries (Fig. 5c).

## Discussion

This present study provides an overview of the DALYs of anxiety, depression, and substance use disorder attributable to childhood maltreatment among adolescents in Africa. Generally, the DALY rate of anxiety and depressive disorders attributable to childhood maltreatment increased significantly aligning with the global increase recorded by the GBD 2021 study [13]. The global burden of mental disorders attributable to childhood maltreatment is an important area of research with substantial implications for public health policies and interventions [34]. It has been estimated that about 1.8 million cases of anxiety, and depressive disorders could be prevented if childhood maltreatment is eliminated [12] implying that comprehensive preventative interventions, improved public health policies, and more funds are crucial to preventing childhood maltreatment and reducing the global burden of mental health disorders.

### Trends in anxiety and depressive disorder

Our study reported variations in the distribution of anxiety and depression, attributable to childhood maltreatment in Africa. A general increase was observed in the number of cases of anxiety and depression. In 2021, Egypt reported the highest cases of anxiety and depression. Child maltreatment is a known prevalent practice across successive generations in Egypt [35]. As such, it may be the probable cause for the elevated number of cases observed in this country. For AAPC, Niger recorded the highest in anxiety, and Burkina Faso recorded the highest in depressive disorders. The inclining trend over this period highlights the prevalence of childhood maltreatment in Africa, coupled with a lack of access to mental health and socio-economic stressors [36]. However, the increasing DALYs could also be reflective of heightened awareness and better reporting mechanisms [37]. Early intervention strategies focusing on trauma recovery and addressing cultural and systemic barriers surrounding access to mental health are crucial to mitigate these issues [38]. In addition, promoting the development of supportive and interconnected social networks can lead to improvements in mental health [39]. Among the 52 African countries, Congo was the only country that recorded the least increase in AAPC for anxiety and a decrease in AAPC for depression, attributed to childhood maltreatment. Given this distinctive pattern, further research into possible protective factors and effective interventions in Congo that may influence this differential trend is required.

### Trends in substance use disorder

In contrast to anxiety and depression, the number of DALY and AAPC in substance use disorders attributable to childhood maltreatment varied in these countries. Although the observed decline may result from effective prevention programs and broader public health initiatives, it could also reflect underreporting and limited access to diagnostic and therapeutic services indicating the need for improved medical and information systems [40]. This inconsistent pattern also suggests that there are other variables at play in the association between substance use disorders and childhood maltreatment, such as physiological and environmental factors, local laws, cultural perspectives, and accessibility to support systems [41, 42]. Noteworthy in Uganda are some fluctuations and dramatic shifts between 1999 to 2007, and 2007 to 2017. This dramatic change could be indicative of changes in policies or societal and cultural shifts that influenced substance use patterns in the country. The disparities emphasize the need for tailored mental health interventions that address each nation’s unique socio-cultural and economic contexts.

### Age, period, and cohort effects

Considering the age, period, and cohort effects on anxiety, depression, and substance use disorder attributable to childhood maltreatment, the DALY rate increased with age, indicating that age is a risk factor for these disorders. The 13–18 age range has been identified as a period of heightened vulnerability for adolescents since it marks the onset of internalizing psychopathology [43, 44]. Other studies have reported that during this age, the prevalence of mental disorders doubles, explaining the sharp increase in ages 15–19 [45]. Specifically for anxiety disorders, the highest peak was observed at ages 15–19, followed by a sharp decline in ages 20–24. The subsequent decline has been associated with the development of coping mechanisms or changes in life situations during the transition into adulthood [46].

Notably, males showed higher DALY rates compared to females for the age effects in all disorders. However, this is in contrast with other studies where females were twice as likely to be depressed compared to males [47]. This discrepancy may be because of the protective factor of masculinity for males [48]. It may also indicate that men are less likely to self-report mental disorders due to the social stigma associated with them [49]. Moreover, gender-related subtypes of mental disorders exist, and this is probably the strongest contribution to the gender gaps [50]. Future research should focus on examining gender differences in core psychological functions and the evolving socioeconomic and cultural trends that contribute to the gender gap.

The period effects on anxiety and depressive disorders attributable to childhood maltreatment depicted an increasing trend over time. More than half of the countries in Africa belong to the low and low-middle SDI category according to GBD [20]. A growing body of international research has shown that in low and middle-income countries, a negative relationship exists between mental illness and poverty [40] indicating that countries belonging to this group have high rates of mental illness because of their low to middle level of income. For the period effect on substance use disorder, a decreasing DALY rate ratio was observed. This downward trend may be due to advancements in preventive psychiatry and effective intervention programs [51]. Based on this, high-quality interventions in low- and middle-income countries must be embarked on. Mental health care in this region needs to be prioritized not only as a public health and human rights priority but also as an economic development priority [40].

The cohort effect revealed interesting gender-specific patterns. Females recorded a higher DALY rate ratio for anxiety and depression attributable to childhood maltreatment. Studies have shown that females are more likely to internalize stressful events, while males tend to externalize usually [52]. By internalizing it, females develop anxiety and depression, explaining the patterns observed in our study. Our results are consistent with results from other studies that reflect the effects of changing societal pressures, and environmental and technological factors on successive birth cohorts [53, 54]. These patterns highlight the significance of generation-specific approaches to substance use disorder attributable to childhood maltreatment prevention and interventions.

Our study has some limitations that need to be acknowledged. Firstly, the impact of the COVID-19 pandemic was not formally incorporated or quantified across risk factors or health outcomes in the 2021 GBD risk estimates [3], which may affect the interpretation of results. Secondly, data for childhood maltreatment (childhood sexual abuse and bullying victimization) in GBD data are sparse, which may result in underestimation [55]. Moreover, the intrinsic estimator approach used on the sparse data may produce biased estimates [29]. Consequently, our results should be interpreted with an understanding of these limitations. Thirdly, our study did not account for the effect of childhood maltreatment on other substance use disorders apart from alcohol use, since there is no available data for this on GBD. Lastly, we did not further analyze the subtypes of childhood maltreatment, which are childhood sexual abuse and bullying victimization. Detailed analysis of these sub classifications can help to better understand their effects on anxiety, depression, and substance use disorders.

## Conclusions

The global burden of the number and rates of DALYs of anxiety and depressive disorders attributable to childhood maltreatment continues to increase with marked variations by sex and geographical location in the past three decades, while substance use disorders have decreased. Effective strategies to eliminate childhood maltreatment in children and adolescents are needed to reduce the burden of anxiety and depressive disorder. Moreover, considering the variations in the burden by age, period, birth cohort, sex, and geographic location, future protective actions should be developed based on every region and country’s unique cultural contexts, developmental stages, and geographical features. Although decreasing trends were observed in substance use disorders attributable to childhood maltreatment, current prevention and intervention must be continued.

## Supplementary Information

Supplementary Material 1.

## Data Availability

The dataset supporting the conclusions of this article is available on the IHME website (https://vizhub.healthdata.org/gbd-results/) [49].

## Abbreviations

AAPC: Average annual percentage change
APC: Annual percentage change
DALY: Disability-adjusted life years
GBD: Global burden of disease
SDI: Socio-demographic index
