# Supplementary material for: The burden of anxiety, depression, and substance use disorders attributable to childhood maltreatment among adolescents in Africa: insights from the global burden of disease study 2021
Source: Ann Gen Psychiatry. 2025 Nov 18;24:68. doi: 10.1186/s12991-025-00611-8 (PMC12625208; doi:10.1186/s12991-025-00611-8)
Supplement: Supplementary file 1 — Supplementary Material 1. [file 12991_2025_611_MOESM1_ESM.pdf]

Table of Contents

Appendix A. Case definitions and cataloging codes for mental and substance use disorders in GBD 2

Appendix B. GBD methods for disability-adjusted life years (DALY) estimation 2

Appendix C. GBD 2021 methods for estimating risk factors 2

Table S1. Number of cases, DALYs rate, and AAPC of anxiety disorders attributable to childhood maltreatment between 1990 and 2021 in 52 African countries...5

Table S2. Number of cases, DALYs rate and AAPC of depressive disorders attributable to childhood maltreatment between 1990 and 2021 in 52 African countries...8

Table S3. Number of cases, DALYs rate and AAPC of substance use disorders attributable to childhood maltreatment between 1990 and 2021 in 52 African countries 11

Table S4. Wald χ^2 test of anxiety, depression and substance use disorder in age, period, and cohort models in 52 African countries 15

Figure S1. DALYs rate per 100, 000 population aged 10-24 years of anxiety (A), depression (B), and substance use disorder(C) attributable to childhood maltreatment in 2021 in 52 African countries. 61

Figure S2. AAPC in DALY rate of anxiety (SA1), depression (SB1), and substance use disorder (SC1) attributed to childhood maltreatment in 10–24-year population in 52 African countries...62

Figure S3. Joinpoint regression analysis of DALY rate of anxiety (A), depression (B), and substance use disorder (C) attributed to childhood maltreatment in the African countries with the top 5 highest recorded AAPC from 1990-2021. 63

Figure S4. Age effects on anxiety disorders attributable to childhood maltreatment in 52 African countries. 64

Figure S5. Age effects on depressive disorders attributable to childhood maltreatment in 52 African countries. 65

Figure S6. Age effects on substance use disorders attributable to childhood maltreatment in 52 African countries. ..66

placce

Figure S8. Period effects on depressive disorders attributable to childhood maltreatment in 52 African countries. ...68

Figure S9. Period effects on substance use disorders attributable to childhood maltreatment in 52 African countries. 69

Figure S10 Cohort effects on anxiety disorders attributable to childhood maltreatment in 52 African countries. 70

Figure S11 Cohort effects on depressive disorders attributable to childhood maltreatment in 52 African countries. .71

Figure S12 Cohort effects on substance use disorders attributable to childhood maltreatment in 52 African countries. 72

Appendix A. Case definitions and cataloging codes for mental and substance use disorders in GBD

- Anxiety disorders: Anxiety disorders involve experiences of intense fear and distress, typically in combination with other physiological symptoms. In GBD, anxiety disorders are modeled as a single cause for "any" anxiety disorder to avoid the double-counting of individuals meeting criteria for more than one anxiety disorder (DSM-IV-TR: 300.0-300.3, 208.3, 309.21, 309.81; ICD-10: F40-42, F43.0, F43.1, F93.0-93.2, F93.8)
- Depressive Disorders: In GBD, depressive disorders are classified into two groups- major depressive disorder (MDD) and dysthymia. MDD is an episodic mood disorder, incorporating disability from the experience of depressed mood or loss of interest/pleasure almost all day, every day, for at least two weeks (DSM-IV-TR: 296.21–24, 296.31–34 ICD-10: F32.0–9, F33.0–9). Dysthymia involves the experience of a chronically depressed mood for most of the day, more days than not, for at least two years (or at least one year in children and adolescents) (DSM-IV-TR: 300.4 ICD-10: F34.1).
- Substance use disorder: This includes deaths assigned to alcohol use disorders or accidental poisoning by alcohol codes and cases of alcohol dependence, a substance-related disorder involving a dysfunctional pattern of alcohol use, and fetal alcohol syndrome (ICD 9: 291-291.9, 303-303.9, 305.0, 357.5, 790.3, E860 ICD-10: E24.4, F10-F10.9, G31.2, G62.1, G72.1, P04.3, Q86.0, R78.0, X45-X45.9, X65- X65.9, Y15- Y15.9).

Appendix B. GBD methods for disability-adjusted life years (DALY) estimation

DALYs are calculated as the sum of years of life lost (YLLs) and years lived with disability (YLDs) accounting for both mortality and morbidity. YLLs measure the difference between an individual's age at death and their life expectancy at the time of their death. YLDs include health loss by considering both the severity and duration of a nonfatal condition.

To generate YLLs by age, sex, year, and location, GBD uses the cause of death (COD) data and the cause of death ensemble model (CODEm). CODEm is the process of modeling each cause using a variety of statistical models and combining them to produce the best estimate. CodCorrect and shocks/fatal discontinuities are used to further adjust for total mortality in each country.

For YLDs, GBD uses non-fatal health outcome data. Disease modeling (DisMod), a Bayesian approach, generates non-fatal health outcomes for locations without data. DisMod takes all available information for a disease, such as incidence, prevalence, remission, and duration, and comes up with an estimate that is consistent with the trends in the various pieces of information that inform the model. GBD then adjusts for the severity of the disease condition and the number of conditions individuals suffer from. Finally, YLDs by age, sex, year, and location are generated.

Appendix C. GBD 2021 methods for estimating risk factors

To estimate risk factors, GBD considers 5 essential components: exposure estimate, relative risk estimate, risk-outcome pairs, theoretical minimum risk exposure level, and total deaths or DALYs.

- Exposure estimate: This is the determination of a risk factor's prevalence
- Relative risk estimate: This is the determination of a risk factor's prevalence
- Risk-outcome pairs: This label is applied when sufficient evidence connects a risk to an outcome
- Theoretical minimum risk exposure level (TMREL): The ideal level of exposure that a population could have to a particular risk factor
- Total deaths/DALYs: This label is applied to all diseases included in risk-outcome pairs

2021 GBD data sources for risk estimation

- Relative risk data estimation as a function of exposure for risk–outcome pair

GBD uses data from primary randomized controlled trials and cohorts, pooled cohorts, or case-control studies that report RRs of mortality or morbidity from a given health outcome as a function of risk exposure, in addition to meta-analyses summarizing relative risks (RRs) to generate RR estimates for risk outcome pairs. In 2021, GBD estimated relationships between 88 risk factors for 631 risk-outcome pairs and did not quantify the impact of COVID-19. In the analytical process, a new method was introduced to complement RR estimates: burden of proof risk function (BPRF)

analyses that account for unexplained between-study heterogeneity in RR input data and yield an additional, conservative interpretation of the risk–outcome association and its underlying input evidence. This was used to synthesize data identified and extracted through systematic reviews based on the Preferred Reporting Items for Systematic Reviews and Meta-Analyses (PRISMA) framework.

- Estimation of the exposure data for each risk by age-sex-location-year

For each risk factor, GBD systematically searched for published studies, household surveys, censuses, administrative data, ground monitor data, or remote sensing data that could inform risk exposure estimates. To estimate mean levels of exposure by age-sex-location-year, specific methods varied across risk factors. For many risk factors, exposure data were modeled using spatiotemporal Gaussian process regression and disease model meta-regression (DisMod-MR 2.1), Bayesian statistical models developed over the past 12 years for GBD analyses. The analysis pools heterogeneous data and controls and adjusts for bias.

Determining theoretical minimum risk exposure levels (TMRELs)

The TMREL was determined as the low point of the risk function. When the bottom of the risk function was flat or poorly determined, the TMREL uncertainty interval (UI) captured the range over which risks are indistinguishable. For protective risks with monotonically declining risk functions with exposure, namely, risk factors where exposure lowers the risk of an outcome, the challenge is selecting the level of exposure with the lowest level of risk strongly supported by the available data. For these cases, GBD determined the exposure level at the 85th percentile of exposure in the cohorts or trials used in the risk meta-regression. The TMREL was then generated by weighing each risk-outcome pair by the relative global magnitude of each outcome.

Estimation of the population-attributable fraction and attributable burden for combinations of risk factors

For each risk factor j, the population attributable fraction (PAF) was computed by age-sex-location-year using the general formula for a continuous risk: $PAF_{joasgt} = \int x{=}l\ RR_{joasg}(x)P_{jasgt}(x)dx - RR_{joasg}\ (TMREL_{jas}) / \int^{u}_{x=l} RR_{joasg}(x)P_{jasgt}(x)dx$.

PAF_{joasgt} is the PAF for cause o, for age group a, sex s, location g, and year t; $RR_{joasg}(x)$ is the relative risk as a function of exposure level x for risk factor j, for cause o controlled for confounding, age group a, sex s, and location g with the lowest level of observed exposure as l and the highest as u; $P_{jasgt}(x)$ is the distribution of exposure at x for age group a, sex s, location g, and year t; and $TMREL_{jas}$ is the TMREL for risk factor j, age group a, and sex s. Where risk exposure is dichotomous or polytomous, this formula simplifies the discrete form of the equation. Estimation of the PAF takes into account the risk function and the distribution of exposure across individuals in each age-sex-location-year. For the estimation of each specific risk factor, the counterfactual distribution of exposure is the TMREL for that specific risk with no change in other risk factors. This implies that the sum of risk-specific estimates of attributable burden can exceed 100%. To estimate the combined effects of risk factors, GBD considers the mediation relationships among risk factors. The mediation matrix was used to correct the overestimation of the PAF and attributable burden for combinations of risks if independence without any mediation between risk factors is assumed and to analyze the burden attributable to combinations of risk factors.

Estimating the summary exposure value (SEV)

The SEV compares the distribution of excess risk times exposure level to a population where everyone is at maximum risk. The SEV is effectively excess risk-weighted prevalence, which allows for comparisons across different types of exposures. Maximum risk in the denominator of the SEV is determined by the relative risk at the 99th percentile of the global distribution of exposure. The SEV is on a 0–100 scale where 100 means the entire population is at maximum risk and 0 means everyone in the population is at minimum risk. Finally, estimates of the proportion of disease burden attributable to the risk factor/attributable burden are calculated based on the product of PAF and DALYs or deaths associated with the outcome for age groups, sex, location, and year.

Accounting for biases and errors

GBD 2021 improved methods for estimating risk exposure and risk-attributable burden by focusing on standardization of RR estimation and application of new burden of proof risk factors (BPRF) methods to generate conservative assessments of risk–outcome relationships. The BPRF approach incorporates differences in exposure ranges for different comparison groups by integrating across the RR function, tests and adjusts for systematic biases to account for identified heterogeneity across input study designs and characteristics, and trims potentially distorting outliers in the input data. Furthermore, GBD improved the specification of the mediation matrix to correct for overestimation of the PAF and the attributable burden for combinations of risks; and re-evaluation of TMRELs with meta-regression or other methods to incorporate new data.

Method Figure S1. Flowchart for DALYs attributable to risk factor estimation.

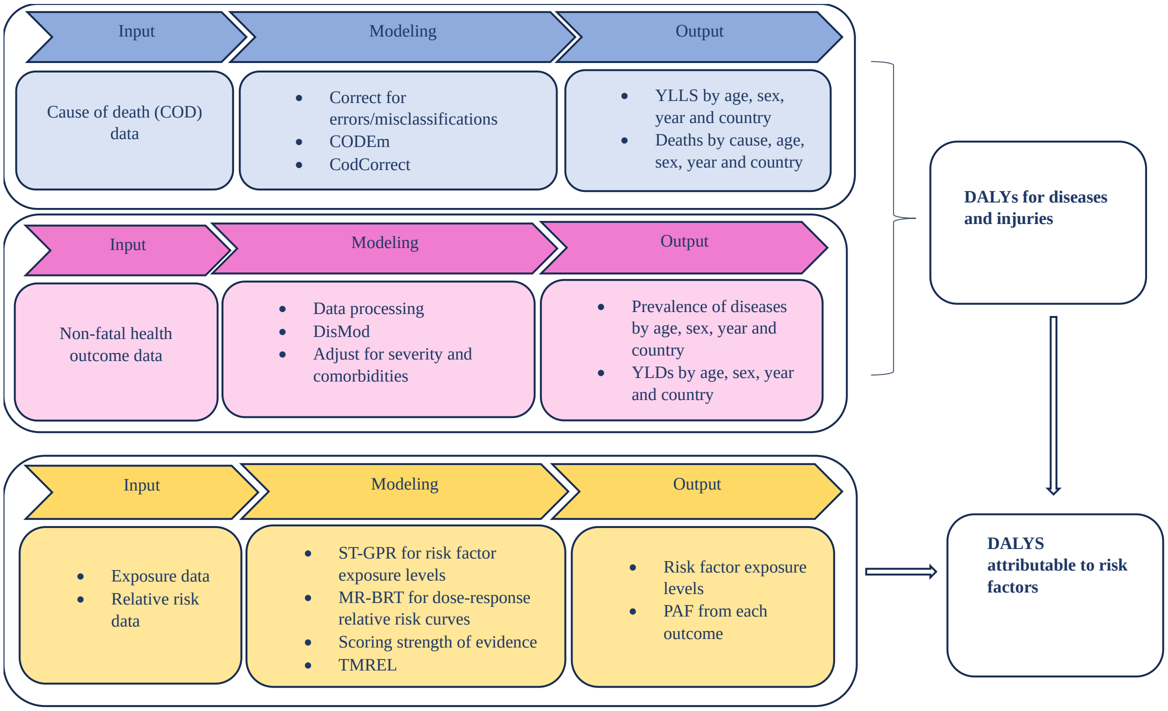

References

1. Global burden of 87 risk factors in 204 countries and territories, 1990-2019: a systematic analysis for the Global Burden of Disease Study 2019. Lancet, 2020. 396(10258): p. 1223-1249.
2. Global prevalence and burden of depressive and anxiety disorders in 204 countries and territories in 2020 due to the COVID-19 pandemic. Lancet, 2021. 398(10312): p. 1700-1712.
3. Kieling, C., et al., Worldwide Prevalence and Disability From Mental Disorders Across Childhood and Adolescence: Evidence From the Global Burden of Disease Study. JAMA Psychiatry, 2024. 81(4): p. 347-356.
4. Global burden and strength of evidence for 88 risk factors in 204 countries and 811 subnational locations, 1990-2021: a systematic analysis for the Global Burden of Disease Study 2021. Lancet, 2024. 403(10440): p. 2162-2203.

Table S1. Number of cases, DALYs rate, and AAPC of anxiety disorders attributable to childhood maltreatment between 1990 and 2021 in 52 African countries

Location	Case(n) 1990	DALYs rate 1990 (per 100,000)	Case(n) 2021	DALYs rate 2021(per 100,000)	AAPC_95%CI	P. Value	Order
Algeria	10536<br>(3598 to 23061)	123.76<br>(42.26 to 270.89)	17309<br>(6608 to 34655)	170.28<br>(65 to 340.91)	1.07<br>(1.04 to 1.11)	< 0.00	30
Angola	2195<br>(754 to 4579)	68.5<br>(23.54 to 142.89)	10035<br>(3579 to 20634)	95.04<br>(33.9 to 195.41)	1.07<br>(1.04 to 1.1)	< 0.00	31
Benin	586<br>(197 to 1298)	40.65<br>(13.67 to 89.93)	2820<br>(1003 to 5871)	64.4<br>(22.91 to 134.08)	1.49<br>(1.41 to 1.56)	< 0.00	18
Botswana	433<br>(162 to 863)	95.93<br>(35.87 to 191.26)	832<br>(316 to 1555)	127.31<br>(48.37 to 237.87)	0.93<br>(0.86 to 0.99)	< 0.00	35
Burkina Faso	752<br>(239 to 1725)	25.66<br>(8.16 to 58.84)	4714<br>(1652 to 9941)	64.85<br>(22.72 to 136.75)	3.06<br>(2.98 to 3.14)	< 0.00	2
Burundi	616<br>(195 to 1370)	36.74<br>(11.63 to 81.68)	3323<br>(1180 to 6692)	76.97<br>(27.34 to 155.01)	2.45<br>(2.39 to 2.5)	< 0.00	3
Cabo Verde	89<br>(32 to 186)	78.1<br>(27.85 to 162.48)	141<br>(49 to 288)	93.72<br>(32.87 to 191.97)	0.59<br>(0.55 to 0.63)	< 0.00	45
Cameroon	2304<br>(796 to 4784)	71.46<br>(24.69 to 148.38)	9173<br>(3273 to 18772)	88.32<br>(31.52 to 180.76)	0.69<br>(0.67 to 0.72)	< 0.00	41
Central African Republic	498<br>(167 to 1033)	59.13<br>(19.83 to 122.6)	1475<br>(489 to 3180)	81.3<br>(26.93 to 175.24)	1.02<br>(0.99 to 1.06)	< 0.00	34
Chad	698<br>(236 to 1547)	38.25<br>(12.91 to 84.74)	3993<br>(1335 to 8379)	68.44<br>(22.89 to 143.63)	1.89<br>(1.85 to 1.93)	< 0.00	11
Comoros	98<br>(33 to 205)	63.58<br>(21.65 to 132.94)	195<br>(66 to 405)	88.59<br>(30.16 to 183.62)	1.05<br>(0.99 to 1.11)	< 0.00	33
Congo	766<br>(267 to 1553)	93.98<br>(32.83 to 190.6)	1718<br>(629 to 3506)	103.07<br>(37.73 to 210.25)	0.29<br>(0.27 to 0.32)	< 0.00	52
Cote d'Ivoire	1998<br>(647 to 4205)	52.28<br>(16.92 to 110)	6482<br>(2277 to 13958)	75.34<br>(26.46 to 162.24)	1.18<br>(1.15 to 1.21)	< 0.00	25
Democratic Republic of the Congo	8425<br>(2912 to 17460)	70.15<br>(24.25 to 145.39)	29863<br>(10467 to 60485)	100.24<br>(35.13 to 203.03)	1.16<br>(1.09 to 1.22)	< 0.00	26
Djibouti	41<br>(13 to 94)	28.22<br>(8.86 to 63.8)	211<br>(74 to 435)	59.25<br>(20.88 to 122.11)	2.41<br>(2.32 to 2.51)	< 0.00	4

Egypt	31969<br>(12715 to 60713)	185.86<br>(73.92 to 352.96)	76537<br>(34457 to 137090)	257.27<br>(115.82 to 460.81)	1.07<br>(1.03 to 1.1)	< 0.00	32
Equatorial Guinea	66<br>(21 to 148)	50.44<br>(16.27 to 113.21)	504<br>(177 to 1056)	92.06<br>(32.36 to 192.94)	1.96<br>(1.92 to 1.99)	< 0.00	9
Eritrea	409<br>(132 to 927)	36.29<br>(11.68 to 82.23)	1250<br>(406 to 2679)	59.96<br>(19.47 to 128.49)	1.59<br>(1.51 to 1.67)	< 0.00	15
Eswatini	184<br>(64 to 387)	66.71<br>(23.29 to 140.29)	307<br>(110 to 604)	83.53<br>(29.9 to 164.1)	0.74<br>(0.68 to 0.79)	< 0.00	39
Ethiopia	5306<br>(1749 to 11439)	32.98<br>(10.87 to 71.11)	23273<br>(8102 to 49066)	61.7<br>(21.48 to 130.07)	1.97<br>(1.93 to 2.02)	< 0.00	7
Gabon	308<br>(110 to 636)	100.41<br>(35.72 to 207.15)	635<br>(225 to 1263)	112.58<br>(39.79 to 223.74)	0.36<br>(0.32 to 0.4)	< 0.00	49
Gambia	185<br>(65 to 388)	59.12<br>(20.91 to 124.09)	698<br>(246 to 1421)	85.69<br>(30.17 to 174.5)	1.2<br>(1.17 to 1.23)	< 0.00	24
Ghana	4158<br>(1433 to 8779)	88.31<br>(30.43 to 186.46)	13628<br>(5992 to 24889)	129.76<br>(57.06 to 236.99)	1.25<br>(1.21 to 1.29)	< 0.00	21
Guinea	606<br>(205 to 1346)	36.19<br>(12.23 to 80.32)	3206<br>(1103 to 6748)	74.02<br>(25.46 to 155.8)	2.33<br>(2.27 to 2.39)	< 0.00	5
Guinea-Bissau	146<br>(49 to 312)	45.27<br>(15.31 to 96.39)	508<br>(174 to 1057)	75.63<br>(25.96 to 157.33)	1.68<br>(1.64 to 1.72)	< 0.00	13
Kenya	5819<br>(2121 to 11859)	73.37<br>(26.74 to 149.54)	16516<br>(6151 to 32975)	95.72<br>(35.65 to 191.1)	0.83<br>(0.79 to 0.86)	< 0.00	37
Lesotho	442<br>(157 to 901)	92.58<br>(32.91 to 188.54)	640<br>(222 to 1271)	104.57<br>(36.31 to 207.71)	0.36<br>(0.31 to 0.41)	< 0.00	48
Liberia	387<br>(126 to 803)	53.18<br>(17.29 to 110.5)	1458<br>(513 to 2930)	81.09<br>(28.5 to 162.94)	1.39<br>(1.31 to 1.46)	< 0.00	20
Libya	1890<br>(683 to 4003)	128.43<br>(46.42 to 272.04)	2549<br>(986 to 5227)	146.21<br>(56.56 to 299.81)	0.44<br>(0.4 to 0.48)	< 0.00	47
Madagascar	2355<br>(784 to 5038)	61.5<br>(20.47 to 131.54)	8666<br>(3120 to 17949)	90.19<br>(32.47 to 186.8)	1.23<br>(1.17 to 1.3)	< 0.00	22
Malawi	2298<br>(782 to 4727)	72.73<br>(24.73 to 149.6)	6741<br>(2481 to 13539)	94.75<br>(34.88 to 190.3)	0.86<br>(0.8 to 0.91)	< 0.00	36

Mali	865<br>(269 to 2010)	33.89<br>(10.56 to 78.79)	4907<br>(1778 to 10147)	61.39<br>(22.24 to 126.95)	1.96<br>(1.91 to 2.01)	< 0.00	8
Mauritania	247<br>(79 to 533)	38.73<br>(12.35 to 83.67)	1002<br>(389 to 2002)	69.73<br>(27.07 to 139.38)	1.93<br>(1.87 to 1.99)	< 0.00	10
Morocco	4013<br>(1256 to 9055)	49.57<br>(15.51 to 111.84)	7525<br>(2679 to 15781)	80.54<br>(28.67 to 168.9)	1.59<br>(1.55 to 1.64)	< 0.00	14
Mozambique	2026<br>(662 to 4362)	49.03<br>(16.02 to 105.56)	8781<br>(3010 to 18096)	82.98<br>(28.44 to 171)	1.69<br>(1.62 to 1.76)	< 0.00	12
Namibia	545<br>(201 to 1061)	114.89<br>(42.28 to 223.58)	986<br>(389 to 1878)	132.2<br>(52.24 to 251.95)	0.46<br>(0.41 to 0.51)	< 0.00	46
Niger	486<br>(147 to 1137)	19.84<br>(5.99 to 46.46)	4395<br>(1500 to 9288)	52.18<br>(17.81 to 110.25)	3.17<br>(3.08 to 3.27)	< 0.00	1
Nigeria	13275<br>(4622 to 27295)	47.03<br>(16.38 to 96.7)	51917<br>(18719 to 106582)	66.61<br>(24.02 to 136.75)	1.14<br>(1.11 to 1.17)	< 0.00	27
Rwanda	1455<br>(511 to 3083)	64.26<br>(22.58 to 136.14)	3897<br>(1393 to 8171)	90.27<br>(32.27 to 189.3)	1.08<br>(1.05 to 1.12)	< 0.00	29
Sao Tome and Principe	32<br>(12 to 65)	79.46<br>(28.45 to 161.83)	69<br>(26 to 133)	97.59<br>(36.66 to 188.5)	0.67<br>(0.63 to 0.71)	< 0.00	42
Senegal	1062<br>(351 to 2324)	43.84<br>(14.5 to 95.94)	3711<br>(1304 to 7784)	70.94<br>(24.92 to 148.78)	1.57<br>(1.55 to 1.6)	< 0.00	17
Sierra Leone	744<br>(247 to 1567)	61.13<br>(20.32 to 128.81)	2595<br>(906 to 5335)	89.19<br>(31.13 to 183.36)	1.23<br>(1.17 to 1.29)	< 0.00	23
Somalia	757<br>(254 to 1642)	29.71<br>(9.96 to 64.48)	4171<br>(1412 to 9027)	57.34<br>(19.41 to 124.1)	2.1<br>(2.05 to 2.16)	< 0.00	6
South Africa	10500<br>(3785 to 21698)	88.88<br>(32.04 to 183.68)	13959<br>(5027 to 28158)	97.4<br>(35.07 to 196.47)	0.3<br>(0.27 to 0.33)	< 0.00	51
South Sudan	976<br>(311 to 2214)	49.16<br>(15.65 to 111.54)	2094<br>(712 to 4516)	61.56<br>(20.93 to 132.78)	0.73<br>(0.69 to 0.77)	< 0.00	40
Sudan	6310<br>(2044 to 13325)	97.69<br>(31.65 to 206.28)	17167<br>(6208 to 35349)	119.6<br>(43.25 to 246.26)	0.65<br>(0.62 to 0.69)	< 0.00	44
Togo	826<br>(293 to 1711)	69.16<br>(24.55 to 143.23)	2564<br>(876 to 5074)	97.92<br>(33.44 to 193.77)	1.13<br>(1.09 to 1.16)	< 0.00	28

Tunisia	2835<br><br>(991 to 6072)	106.02<br><br>(37.06 to 227.07)	3257<br><br>(1190 to 6789)	129.74<br><br>(47.38 to 270.43)	0.66<br><br>(0.63 to 0.69)	< 0.00	43
Uganda	3074<br><br>(1013 to 6660)	54.41<br><br>(17.94 to 117.88)	13276<br><br>(4638 to 26740)	87.51<br><br>(30.57 to 176.26)	1.58<br><br>(1.52 to 1.64)	< 0.00	16
United Republic of Tanzania	2819<br><br>(878 to 6128)	33.07<br><br>(10.3 to 71.89)	9994<br><br>(3395 to 20605)	51.82<br><br>(17.61 to 106.84)	1.47<br><br>(1.4 to 1.53)	< 0.00	19
Zambia	3175<br><br>(1175 to 6223)	114.48<br><br>(42.37 to 224.35)	9667<br><br>(3741 to 17905)	146.43<br><br>(56.67 to 271.21)	0.81<br><br>(0.77 to 0.85)	< 0.00	38
Zimbabwe	3694<br><br>(1326 to 7458)	102.86<br><br>(36.92 to 207.64)	5730<br><br>(2274 to 10508)	112.28<br><br>(44.56 to 205.91)	0.3<br><br>(0.27 to 0.33)	< 0.00	50

Note: Order shows ranking from the highest to lowest AAPC of DALYs in anxiety disorders attributable to childhood maltreatment among the 10–24-year population in 52 African countries. The first 5 in the highest ranking are highlighted in red. DALYs: disability-adjusted life years, AAPC: average annual percentage change.

Table S2. Number of cases, DALYs rate and AAPC of depressive disorders attributable to childhood maltreatment between 1990 and 2021 in 52 African countries

**Location**	**Case(n) 1990**	**DALYs rate 1990 (per 100,000)**	**Case(n) 2021**	**DALYs rate 2021(per 100,000)**	**AAPC_95%UI**	**P-Value**	**Order**
Algeria	10407<br><br>(4078 to 20073)	122.25<br><br>(47.9 to 235.79)	16359<br><br>(7126 to 31261)	160.93<br><br>(70.1 to 307.53)	0.92<br><br>(0.87 to 0.96)	< 0.00	27
Angola	4775<br><br>(2117 to 9258)	149<br><br>(66.07 to 288.92)	18680<br><br>(8309 to 34248)	176.91<br><br>(78.69 to 324.35)	0.62<br><br>(0.6 to 0.64)	< 0.00	38
Benin	793<br><br>(339 to 1543)	54.94<br><br>(23.48 to 106.96)	3469<br><br>(1610 to 6288)	79.21<br><br>(36.77 to 143.6)	1.1<br><br>(1.04 to 1.16)	< 0.00	16
Botswana	472<br><br>(209 to 875)	104.65<br><br>(46.37 to 193.95)	959<br><br>(448 to 1691)	146.65<br><br>(68.48 to 258.64)	0.93<br><br>(0.85 to 1.02)	< 0.00	25
Burkina Faso	1217<br><br>(513 to 2306)	41.53<br><br>(17.5 to 78.68)	5734<br><br>(2433 to 10817)	78.87<br><br>(33.47 to 148.8)	1.96<br><br>(1.85 to 2.08)	< 0.00	1
Burundi	1148<br><br>(514 to 2218)	68.44<br><br>(30.65 to 132.21)	4387<br><br>(1946 to 8135)	101.63<br><br>(45.07 to 188.43)	1.29<br><br>(1.21 to 1.36)	< 0.00	11
Cabo Verde	122<br><br>(52 to 222)	106.63<br><br>(45.3 to 194.28)	206<br><br>(88 to 376)	137.37<br><br>(58.77 to 250.35)	0.66<br><br>(0.58 to 0.74)	< 0.00	33
Cameroon	2795<br><br>(1178 to 5072)	86.7<br><br>(36.55 to 157.33)	11069<br><br>(4649 to 20463)	106.59<br><br>(44.76 to 197.03)	0.65<br><br>(0.6 to 0.7)	< 0.00	34

Central African Republic	1087 (475 to 2075)	129.04 (56.44 to 246.35)	2846 (1229 to 5563)	156.87 (67.73 to 306.61)	0.64 (0.61 to 0.66)	< 0.00	36
Chad	1180 (518 to 2269)	64.65 (28.38 to 124.26)	5741 (2385 to 11066)	98.41 (40.88 to 189.69)	1.38 (1.31 to 1.45)	< 0.00	8
Comoros	122 (52 to 230)	79.4 (33.57 to 149.23)	222 (94 to 412)	100.56 (42.69 to 186.93)	0.64 (0.61 to 0.67)	< 0.00	37
Congo	1573 (693 to 3001)	193.08 (85.04 to 368.37)	3187 (1391 to 5945)	191.16 (83.4 to 356.55)	-0.06 (-0.11 to -0.01)	< 0.00	52
CÃ´te d'Ivoire	2104 (872 to 4035)	55.03 (22.82 to 105.57)	6140 (2564 to 11773)	71.36 (29.8 to 136.84)	0.85 (0.79 to 0.9)	< 0.00	28
Democratic Republic of the Congo	17032 (7447 to 31404)	141.82 (62.02 to 261.5)	54838 (23727 to 102686)	184.07 (79.64 to 344.68)	0.83 (0.79 to 0.86)	< 0.00	30
Djibouti	72 (30 to 135)	49.08 (20.15 to 91.89)	300 (125 to 561)	84.38 (35.27 to 157.64)	1.76 (1.68 to 1.84)	< 0.00	4
Egypt	28017 (12198 to 52679)	162.88 (70.92 to 306.26)	67948 (34947 to 118505)	228.4 (117.47 to 398.34)	1.09 (1.06 to 1.12)	< 0.00	17
Equatorial Guinea	153 (64 to 309)	117.2 (49.04 to 237.15)	1008 (445 to 1887)	184.01 (81.27 to 344.71)	1.33 (1.28 to 1.38)	< 0.00	10
Eritrea	690 (288 to 1312)	61.2 (25.57 to 116.4)	1796 (763 to 3316)	86.16 (36.6 to 159.08)	0.99 (0.94 to 1.03)	< 0.00	20
Eswatini	223 (96 to 433)	81.02 (34.78 to 157.32)	397 (177 to 718)	107.97 (47.99 to 195.24)	0.77 (0.69 to 0.85)	< 0.00	31
Ethiopia	9607 (4346 to 17596)	59.72 (27.01 to 109.37)	32256 (14909 to 58658)	85.51 (39.53 to 155.5)	1 (0.91 to 1.09)	< 0.00	19
Gabon	610 (271 to 1157)	198.71 (88.2 to 376.71)	1185 (536 to 2152)	209.99 (95.06 to 381.34)	0.13 (0.1 to 0.16)	< 0.00	51
Gambia	298 (129 to 561)	95.09 (41.28 to 179.33)	1056 (451 to 1952)	129.6 (55.34 to 239.6)	0.97 (0.93 to 1.01)	< 0.00	22
Ghana	5077 (2161 to 9586)	107.83 (45.89 to 203.6)	15621 (7708 to 27380)	148.74 (73.39 to 260.7)	0.96 (0.9 to 1.02)	< 0.00	23
Guinea	801 (352 to 1502)	47.83 (21.02 to 89.67)	3611 (1563 to 6863)	83.37 (36.08 to 158.46)	1.77 (1.72 to 1.83)	< 0.00	3

Guinea-Bissau	187<br>(77 to 360)	57.71<br>(23.91 to 111.21)	602<br>(256 to 1155)	89.57<br>(38.12 to 171.77)	1.38<br>(1.32 to 1.44)	< 0.00	7
Kenya	8220<br>(3884 to 15092)	103.64<br>(48.98 to 190.3)	21645<br>(10324 to 39227)	125.44<br>(59.83 to 227.33)	0.52<br>(0.47 to 0.56)	< 0.00	41
Lesotho	742<br>(323 to 1406)	155.4<br>(67.65 to 294.26)	1087<br>(472 to 1989)	177.66<br>(77.13 to 324.99)	0.18<br>(0.13 to 0.23)	< 0.00	50
Liberia	503<br>(222 to 930)	69.14<br>(30.51 to 127.9)	1688<br>(730 to 3172)	93.87<br>(40.57 to 176.38)	0.93<br>(0.73 to 1.12)	< 0.00	26
Libya	1844<br>(745 to 3532)	125.32<br>(50.63 to 240.05)	2600<br>(1182 to 4917)	149.14<br>(67.8 to 282.01)	0.42<br>(0.38 to 0.47)	< 0.00	45
Madagascar	3329<br>(1378 to 6271)	86.91<br>(35.98 to 163.74)	10971<br>(4752 to 19961)	114.18<br>(49.45 to 207.74)	0.84<br>(0.81 to 0.88)	< 0.00	29
Malawi	2778<br>(1191 to 5131)	87.91<br>(37.7 to 162.37)	7513<br>(3413 to 13419)	105.6<br>(47.98 to 188.62)	0.59<br>(0.54 to 0.64)	< 0.00	39
Mali	1022<br>(426 to 1908)	40.05<br>(16.71 to 74.77)	4897<br>(2133 to 9103)	61.26<br>(26.69 to 113.88)	1.35<br>(1.29 to 1.42)	< 0.00	9
Mauritania	308<br>(126 to 613)	48.39<br>(19.72 to 96.28)	1030<br>(461 to 1884)	71.73<br>(32.09 to 131.19)	1.28<br>(1.17 to 1.38)	< 0.00	12
Morocco	5491<br>(2144 to 10980)	67.83<br>(26.48 to 135.62)	9361<br>(4017 to 17678)	100.19<br>(42.99 to 189.2)	1.2<br>(1.14 to 1.26)	< 0.00	13
Mozambique	2547<br>(1007 to 5104)	61.63<br>(24.37 to 123.51)	10614<br>(4264 to 20234)	100.31<br>(40.29 to 191.21)	1.51<br>(1.44 to 1.59)	< 0.00	6
Namibia	511<br>(226 to 941)	107.78<br>(47.7 to 198.36)	894<br>(408 to 1611)	119.88<br>(54.7 to 216.03)	0.29<br>(0.18 to 0.39)	< 0.00	49
Niger	848<br>(365 to 1580)	34.65<br>(14.9 to 64.54)	5308<br>(2249 to 10014)	63.01<br>(26.7 to 118.87)	1.9<br>(1.85 to 1.96)	< 0.00	2
Nigeria	20827<br>(10175 to 36583)	73.79<br>(36.05 to 129.61)	63126<br>(29938 to 111338)	80.99<br>(38.41 to 142.85)	0.35<br>(0.25 to 0.44)	< 0.00	48
Rwanda	2291<br>(976 to 4390)	101.19<br>(43.11 to 193.88)	5252<br>(2257 to 9985)	121.68<br>(52.29 to 231.32)	0.48<br>(0.4 to 0.56)	< 0.00	43
Sao Tome and Principe	33<br>(14 to 62)	82.63<br>(34.96 to 152.59)	72<br>(31 to 138)	102.19<br>(44.41 to 195.14)	0.59<br>(0.54 to 0.64)	< 0.00	40

Senegal	1296 (574 to 2367)	53.51 (23.69 to 97.71)	4102 (1676 to 7777)	78.41 (32.03 to 148.65)	1.19 (1.11 to 1.28)	< 0.00	14
Sierra Leone	859 (358 to 1645)	70.65 (29.4 to 135.24)	2883 (1221 to 5530)	99.08 (41.95 to 190.04)	1.08 (0.99 to 1.16)	< 0.00	18
Somalia	1285 (567 to 2439)	50.47 (22.27 to 95.76)	6283 (2662 to 12027)	86.37 (36.59 to 165.33)	1.58 (1.51 to 1.64)	< 0.00	5
South Africa	12123 (5627 to 22938)	102.62 (47.64 to 194.17)	16674 (7732 to 30815)	116.34 (53.95 to 215.01)	0.4 (0.35 to 0.45)	< 0.00	46
South Sudan	1302 (515 to 2602)	65.62 (25.97 to 131.08)	2553 (1086 to 4901)	75.07 (31.92 to 144.1)	0.51 (0.46 to 0.57)	< 0.00	42
Sudan	7064 (2905 to 13547)	109.36 (44.98 to 209.73)	18423 (7617 to 36197)	128.35 (53.07 to 252.17)	0.47 (0.41 to 0.52)	< 0.00	44
Togo	934 (388 to 1691)	78.16 (32.5 to 141.58)	2784 (1206 to 5169)	106.31 (46.03 to 197.4)	0.94 (0.89 to 1)	< 0.00	24
Tunisia	3550 (1472 to 6986)	132.75 (55.04 to 261.26)	4157 (1756 to 7698)	165.59 (69.96 to 306.64)	0.7 (0.67 to 0.73)	< 0.00	32
Uganda	6761 (2991 to 12937)	119.66 (52.94 to 228.99)	25877 (11511 to 47290)	170.57 (75.88 to 311.71)	1.16 (0.99 to 1.32)	< 0.00	15
United Republic of Tanzania	5012 (2146 to 9337)	58.8 (25.18 to 109.53)	15496 (6924 to 27889)	80.35 (35.9 to 144.61)	0.97 (0.92 to 1.02)	< 0.00	21
Zambia	3279 (1475 to 6053)	118.23 (53.16 to 218.23)	9519 (4524 to 16679)	144.19 (68.53 to 252.65)	0.64 (0.56 to 0.72)	< 0.00	35
Zimbabwe	2691 (1171 to 4924)	74.92 (32.59 to 137.1)	4366 (2008 to 8128)	85.56 (39.34 to 159.27)	0.38 (0.3 to 0.46)	< 0.00	47

Note: Order shows ranking from the highest to lowest AAPC of DALYs in depressive disorders attributable to childhood maltreatment among the 10–24-year population in 52 African countries. The first 5 in the highest ranking are highlighted in red. DALYs: disability-adjusted life years, AAPC: average annual percentage change.

Table S3. Number of cases, DALYs rate and AAPC of substance use disorders attributable to childhood maltreatment between 1990 and 2021 in 52 African countries

Location	Case(n) 1990	DALYs rate 1990 (per 100,000)	Case(n) 2021	DALYs rate 2021(per 100,000)	AAPC_95%UI	P-Value	Order

Algeria	95<br>(13 to 281)	1.12<br>(0.15 to 3.3)	105<br>(13 to 298)	1.04<br>(0.13 to 2.93)	-0.27<br>(-0.41 to -0.14)	< 0.00	44
Angola	123<br>(18 to 329)	3.83<br>(0.55 to 10.25)	374<br>(52 to 1102)	3.54<br>(0.49 to 10.43)	-0.1<br>(-0.25 to 0.05)	< 0.00	29
Benin	34<br>(5 to 97)	2.38<br>(0.31 to 6.75)	110<br>(16 to 311)	2.5<br>(0.37 to 7.11)	0.04<br>(-0.07 to 0.15)	< 0.00	13
Botswana	31<br>(4 to 91)	6.89<br>(0.99 to 20.15)	46<br>(7 to 128)	7.01<br>(1.01 to 19.51)	-0.31<br>(-0.4 to -0.22)	< 0.00	46
Burkina Faso	69<br>(10 to 197)	2.37<br>(0.34 to 6.71)	190<br>(30 to 505)	2.62<br>(0.42 to 6.95)	0.13<br>(0.04 to 0.21)	< 0.00	7
Burundi	96<br>(14 to 295)	5.75<br>(0.82 to 17.58)	220<br>(31 to 616)	5.11<br>(0.71 to 14.26)	-0.36<br>(-0.49 to -0.24)	< 0.00	48
Cabo Verde	3<br>(0 to 8)	2.48<br>(0.3 to 7.42)	4<br>(1 to 11)	2.66<br>(0.42 to 7.25)	-0.09<br>(-0.19 to 0.02)	< 0.00	26
Cameroon	82<br>(10 to 228)	2.53<br>(0.31 to 7.06)	267<br>(41 to 704)	2.57<br>(0.39 to 6.78)	0.01<br>(-0.08 to 0.1)	< 0.00	16
Central African Republic	35<br>(5 to 96)	4.12<br>(0.61 to 11.36)	69<br>(10 to 188)	3.8<br>(0.58 to 10.37)	-0.2<br>(-0.3 to -0.11)	< 0.00	37
Chad	43<br>(7 to 128)	2.38<br>(0.39 to 7.04)	141<br>(22 to 376)	2.42<br>(0.38 to 6.44)	0.16<br>(0.06 to 0.26)	< 0.00	5
Comoros	8<br>(1 to 22)	5.09<br>(0.57 to 14.56)	12<br>(2 to 36)	5.52<br>(0.82 to 16.49)	-0.07<br>(-0.19 to 0.04)	< 0.00	23
Congo	32<br>(5 to 88)	3.94<br>(0.62 to 10.86)	63<br>(10 to 179)	3.78<br>(0.57 to 10.72)	-0.13<br>(-0.27 to 0.02)	< 0.00	31
Cote d'Ivoire	78<br>(12 to 224)	2.04<br>(0.31 to 5.85)	174<br>(19 to 490)	2.02<br>(0.22 to 5.69)	0.01<br>(-0.04 to 0.06)	< 0.00	17
Democratic Republic of the Congo	451<br>(68 to 1271)	3.76<br>(0.57 to 10.58)	1040<br>(164 to 3159)	3.49<br>(0.55 to 10.6)	-0.23<br>(-0.31 to -0.15)	< 0.00	40
Djibouti	9<br>(1 to 26)	5.79<br>(0.78 to 17.54)	20<br>(2 to 62)	5.75<br>(0.66 to 17.36)	-0.15<br>(-0.35 to 0.05)	< 0.00	32
Egypt	173<br>(21 to 515)	1<br>(0.12 to 2.99)	285<br>(43 to 809)	0.96<br>(0.14 to 2.72)	-0.24<br>(-0.34 to -0.13)	< 0.00	42

Equatorial Guinea	5<br>(1 to 14)	3.84<br>(0.54 to 10.87)	21<br>(3 to 68)	3.83<br>(0.54 to 12.42)	-0.21<br>(-0.33 to -0.09)	< 0.00	39
Eritrea	59<br>(7 to 180)	5.24<br>(0.62 to 15.98)	118<br>(16 to 343)	5.65<br>(0.75 to 16.46)	-0.02<br>(-0.11 to 0.07)	< 0.00	20
Eswatini	8<br>(1 to 21)	2.78<br>(0.42 to 7.63)	8<br>(1 to 22)	2.08<br>(0.29 to 5.93)	-1.23<br>(-1.35 to -1.11)	0.97	52
Ethiopia	1120<br>(142 to 3229)	6.96<br>(0.88 to 20.07)	2887<br>(378 to 8273)	7.65<br>(1 to 21.93)	0.22<br>(0.12 to 0.31)	< 0.00	3
Gabon	13<br>(2 to 36)	4.13<br>(0.59 to 11.88)	22<br>(3 to 61)	3.85<br>(0.47 to 10.73)	-0.3<br>(-0.49 to -0.12)	< 0.00	45
Gambia	8<br>(1 to 24)	2.5<br>(0.43 to 7.68)	22<br>(3 to 61)	2.65<br>(0.4 to 7.44)	0.14<br>(0 to 0.28)	< 0.00	6
Ghana	124<br>(19 to 334)	2.64<br>(0.4 to 7.1)	276<br>(45 to 782)	2.63<br>(0.42 to 7.45)	-0.2<br>(-0.3 to -0.1)	< 0.00	36
Guinea	40<br>(5 to 110)	2.38<br>(0.31 to 6.55)	106<br>(15 to 288)	2.45<br>(0.35 to 6.66)	0.07<br>(-0.08 to 0.22)	< 0.00	10
Guinea-Bissau	8<br>(1 to 24)	2.55<br>(0.43 to 7.45)	18<br>(3 to 51)	2.68<br>(0.43 to 7.59)	0.04<br>(-0.09 to 0.18)	< 0.00	12
Kenya	310<br>(55 to 811)	3.91<br>(0.69 to 10.23)	605<br>(119 to 1546)	3.5<br>(0.69 to 8.96)	-0.57<br>(-0.6 to -0.55)	< 0.00	50
Lesotho	18<br>(2 to 55)	3.79<br>(0.43 to 11.43)	30<br>(4 to 88)	4.83<br>(0.66 to 14.44)	0.22<br>(0.16 to 0.28)	< 0.00	2
Liberia	18<br>(3 to 51)	2.49<br>(0.41 to 7)	46<br>(7 to 127)	2.58<br>(0.36 to 7.05)	0.11<br>(0.02 to 0.2)	< 0.00	8
Libya	15<br>(2 to 44)	1.02<br>(0.11 to 2.99)	20<br>(3 to 62)	1.17<br>(0.15 to 3.54)	-0.01<br>(-0.21 to 0.19)	< 0.00	19
Madagascar	206<br>(25 to 608)	5.38<br>(0.66 to 15.86)	499<br>(60 to 1379)	5.19<br>(0.62 to 14.35)	-0.23<br>(-0.36 to -0.11)	< 0.00	41
Malawi	220<br>(33 to 631)	6.96<br>(1.03 to 19.97)	469<br>(61 to 1291)	6.6<br>(0.86 to 18.15)	-0.11<br>(-0.2 to -0.01)	< 0.00	30
Mali	60<br>(8 to 169)	2.37<br>(0.33 to 6.64)	188<br>(25 to 524)	2.35<br>(0.31 to 6.55)	-0.09<br>(-0.2 to 0.02)	< 0.00	27

Mauritania	15<br>(2 to 40)	2.32<br>(0.34 to 6.3)	32<br>(5 to 89)	2.2<br>(0.35 to 6.2)	-0.16<br>(-0.27 to -0.06)	< 0.00	34
Morocco	91<br>(10 to 254)	1.12<br>(0.12 to 3.14)	102<br>(13 to 312)	1.09<br>(0.14 to 3.34)	-0.24<br>(-0.32 to -0.15)	< 0.00	43
Mozambique	103<br>(14 to 349)	2.5<br>(0.34 to 8.44)	286<br>(32 to 986)	2.7<br>(0.3 to 9.32)	0.05<br>(-0.03 to 0.13)	< 0.00	11
Namibia	14<br>(2 to 45)	2.86<br>(0.33 to 9.48)	22<br>(3 to 77)	3.01<br>(0.36 to 10.27)	0.03<br>(-0.08 to 0.13)	< 0.00	15
Niger	57<br>(9 to 161)	2.32<br>(0.37 to 6.56)	193<br>(29 to 540)	2.29<br>(0.34 to 6.42)	-0.04<br>(-0.17 to 0.08)	< 0.00	22
Nigeria	1386<br>(253 to 3346)	4.91<br>(0.9 to 11.85)	3330<br>(580 to 7607)	4.27<br>(0.74 to 9.76)	-0.35<br>(-0.39 to -0.31)	< 0.00	47
Rwanda	143<br>(16 to 432)	6.3<br>(0.71 to 19.07)	277<br>(31 to 860)	6.41<br>(0.72 to 19.93)	-0.18<br>(-0.51 to 0.15)	< 0.00	35
Sao Tome and Principe	1<br>(0 to 3)	2.26<br>(0.35 to 7.11)	2<br>(0 to 5)	2.51<br>(0.35 to 7.03)	0.07<br>(-0.03 to 0.17)	< 0.00	9
Senegal	57<br>(9 to 153)	2.36<br>(0.36 to 6.32)	127<br>(17 to 372)	2.43<br>(0.32 to 7.12)	-0.08<br>(-0.27 to 0.11)	< 0.00	24
Sierra Leone	32<br>(4 to 90)	2.63<br>(0.34 to 7.42)	78<br>(12 to 205)	2.68<br>(0.43 to 7.04)	0<br>(-0.1 to 0.1)	< 0.00	18
Somalia	120<br>(16 to 382)	4.72<br>(0.64 to 15.01)	366<br>(45 to 1106)	5.03<br>(0.61 to 15.2)	-0.16<br>(-0.23 to -0.09)	< 0.00	33
South Africa	835<br>(119 to 2294)	7.07<br>(1.01 to 19.42)	795<br>(106 to 2083)	5.55<br>(0.74 to 14.54)	-0.91<br>(-1.2 to -0.62)	< 0.33	51
South Sudan	108<br>(13 to 331)	5.45<br>(0.67 to 16.66)	162<br>(21 to 515)	4.75<br>(0.61 to 15.13)	-0.21<br>(-0.29 to -0.12)	< 0.00	38
Sudan	74<br>(11 to 229)	1.14<br>(0.17 to 3.55)	151<br>(20 to 459)	1.05<br>(0.14 to 3.2)	-0.4<br>(-0.46 to -0.34)	< 0.00	49
Togo	30<br>(4 to 84)	2.47<br>(0.35 to 7)	66<br>(8 to 183)	2.53<br>(0.31 to 7)	-0.04<br>(-0.18 to 0.11)	< 0.00	21
Tunisia	30<br>(4 to 91)	1.11<br>(0.14 to 3.4)	27<br>(4 to 82)	1.09<br>(0.16 to 3.28)	-0.1<br>(-0.23 to 0.03)	< 0.00	28

Uganda	180 (21 to 499)	3.19 (0.37 to 8.83)	583 (86 to 1656)	3.84 (0.57 to 10.92)	0.65 (0.59 to 0.72)	< 0.00	1
United Republic of Tanzania	515 (71 to 1423)	6.04 (0.83 to 16.69)	1215 (180 to 3347)	6.3 (0.93 to 17.36)	0.04 (-0.05 to 0.12)	< 0.00	14
Zambia	149 (21 to 426)	5.36 (0.76 to 15.38)	383 (52 to 1143)	5.8 (0.79 to 17.31)	0.17 (0.1 to 0.23)	< 0.00	4
Zimbabwe	130 (16 to 376)	3.61 (0.44 to 10.48)	188 (27 to 565)	3.69 (0.54 to 11.07)	-0.08 (-0.15 to -0.01)	< 0.00	25

Note: Order shows ranking from the highest to lowest AAPC of DALYs in substance use disorder attributable to childhood maltreatment among the 10–24-year population in 52 African countries. The first 5 in the highest ranking are highlighted in red. DALYs: disability-adjusted life years, AAPC: average annual percentage change.

Table S4. Wald χ^2 test of anxiety, depression and substance use disorder in age, period, and cohort models in 52 African countries

		Anxiety				Depression				Substance use			
**Location**	**Sex**	**Wald Tests**	**X^2**	**df**	**P-value**	**Wald Tests**	**X^2**	**df**	**P-value**	**Wald Tests**	**X^2**	**df**	**P-value**
Algeria	Both	Net Drift = 0	755.2395	1	< 0.00	Net Drift = 0	481.9483	1	< 0.00	Net Drift = 0	0.068362	1	0.79
		All Age Deviations = 0	685.5033	1	< 0.00	All Age Deviations = 0	2312.929	1	< 0.00	All Age Deviations = 0	31.72659	1	< 0.00
		All Period Deviations = 0	1.838253	4	0.77	All Period Deviations = 0	8.937416	4	< 0.06	All Period Deviations = 0	0.026946	4	1.00
		All Cohort Deviations = 0	75.8138	6	< 0.00	All Cohort Deviations = 0	22.93829	6	< 0.00	All Cohort Deviations = 0	0.053063	6	1.00
		All Period RR = 1	760.5726	5	< 0.00	All Period RR = 1	521.0214	5	< 0.00	All Period RR = 1	0.127564	5	1.00
		All Cohort RR = 1	855.1034	7	< 0.00	All Cohort RR = 1	609.815	7	< 0.00	All Cohort RR = 1	0.539256	7	1.00
		All Local Drifts = Net Drift	69.60402	3	< 0.00	All Local Drifts = Net Drift	18.74656	3	< 0.00	All Local Drifts = Net Drift	0.044151	3	1.00
	Male	Net Drift = 0	102.9403	1	< 0.00	Net Drift = 0	51.23239	1	< 0.00	Net Drift = 0	0.460278	1	0.50
		All Age Deviations = 0	326.4563	1	< 0.00	All Age Deviations = 0	1008.736	1	< 0.00	All Age Deviations = 0	12.97277	1	< 0.00
		All Period Deviations = 0	1.574932	4	0.81	All Period Deviations = 0	4.583272	4	0.33	All Period Deviations = 0	0.057781	4	1.00
		All Cohort Deviations = 0	7.514647	6	0.28	All Cohort Deviations = 0	1.081144	6	0.98	All Cohort Deviations = 0	0.180791	6	1.00
		All Period RR = 1	105.9074	5	< 0.00	All Period RR = 1	58.75536	5	< 0.00	All Period RR = 1	0.492821	5	0.99

		All Cohort RR = 1	113.5795	7	< 0.00	All Cohort RR = 1	60.5397	7	< 0.00	All Cohort RR = 1	0.895635	7	1.00
		All Local Drifts = Net Drift	7.230315	3	< 0.06	All Local Drifts = Net Drift	0.729314	3	0.87	All Local Drifts = Net Drift	0.175137	3	0.98
	Female	Net Drift = 0	810.1762	1	< 0.00	Net Drift = 0	557.1295	1	< 0.00	Net Drift = 0	0.011361	1	0.92
		All Age Deviations = 0	369.3829	1	< 0.00	All Age Deviations = 0	1311.282	1	< 0.00	All Age Deviations = 0	17.34619	1	< 0.00
		All Period Deviations = 0	0.482761	4	0.98	All Period Deviations = 0	5.736896	4	0.22	All Period Deviations = 0	0.089499	4	1.00
		All Cohort Deviations = 0	81.79016	6	< 0.00	All Cohort Deviations = 0	39.67106	6	< 0.00	All Cohort Deviations = 0	0.224854	6	1.00
		All Period RR = 1	815.1577	5	< 0.00	All Period RR = 1	601.0033	5	< 0.00	All Period RR = 1	0.089702	5	1.00
		All Cohort RR = 1	931.5721	7	< 0.00	All Cohort RR = 1	719.1934	7	< 0.00	All Cohort RR = 1	0.270783	7	1.00
		All Local Drifts = Net Drift	73.14533	3	< 0.00	All Local Drifts = Net Drift	32.45535	3	< 0.00	All Local Drifts = Net Drift	0.207649	3	0.98
Angola	Both	Net Drift = 0	241.0863	1	< 0.00	Net Drift = 0	167.2359	1	< 0.00	Net Drift = 0	0.022168	1	0.88
		All Age Deviations = 0	175.3246	1	< 0.00	All Age Deviations = 0	2129.077	1	< 0.00	All Age Deviations = 0	62.16007	1	< 0.00
		All Period Deviations = 0	0.066254	4	1.00	All Period Deviations = 0	1.615123	4	0.81	All Period Deviations = 0	0.062635	4	1.00
		All Cohort Deviations = 0	5.644124	6	0.46	All Cohort Deviations = 0	24.11828	6	< 0.00	All Cohort Deviations = 0	0.052613	6	1.00
		All Period RR = 1	250.6869	5	< 0.00	All Period RR = 1	204.4745	5	< 0.00	All Period RR = 1	0.094508	5	1.00
		All Cohort RR = 1	267.9994	7	< 0.00	All Cohort RR = 1	170.5286	7	< 0.00	All Cohort RR = 1	0.061972	7	1.00
		All Local Drifts = Net Drift	4.96985	3	0.17	All Local Drifts = Net Drift	23.75127	3	< 0.00	All Local Drifts = Net Drift	0.025546	3	1.00
	Male	Net Drift = 0	98.28348	1	< 0.00	Net Drift = 0	53.11199	1	< 0.00	Net Drift = 0	0.113464	1	0.74
		All Age Deviations = 0	87.58266	1	< 0.00	All Age Deviations = 0	1054.266	1	< 0.00	All Age Deviations = 0	27.8065	1	< 0.00
		All Period Deviations = 0	0.079583	4	1.00	All Period Deviations = 0	1.065031	4	0.90	All Period Deviations = 0	0.230052	4	0.99
		All Cohort Deviations = 0	1.365897	6	0.97	All Cohort Deviations = 0	5.706575	6	0.46	All Cohort Deviations = 0	0.286124	6	1.00
		All Period RR = 1	101.7049	5	< 0.00	All Period RR = 1	68.35579	5	< 0.00	All Period RR = 1	0.272042	5	1.00
		All Cohort RR = 1	105.9215	7	< 0.00	All Cohort RR = 1	54.7653	7	< 0.00	All Cohort RR = 1	0.286129	7	1.00
		All Local Drifts = Net Drift	1.302867	3	0.73	All Local Drifts = Net Drift	5.458943	3	0.14	All Local Drifts = Net Drift	0.276678	3	0.96
	Female	Net Drift = 0	155.0621	1	< 0.00	Net Drift = 0	125.569	1	< 0.00	Net Drift = 0	0.022008	1	0.88

		All Age Deviations = 0	89.25758	1	< 0.00	All Age Deviations = 0	1075.831	1	< 0.00	All Age Deviations = 0	34.56383	1	< 0.00
		All Period Deviations = 0	0.18248	4	1.00	All Period Deviations = 0	1.142908	4	0.89	All Period Deviations = 0	0.059849	4	1.00
		All Cohort Deviations = 0	5.224952	6	0.52	All Cohort Deviations = 0	19.21835	6	< 0.00	All Cohort Deviations = 0	0.00645	6	1.00
		All Period RR = 1	161.7655	5	< 0.00	All Period RR = 1	148.8224	5	< 0.00	All Period RR = 1	0.124966	5	1.00
		All Cohort RR = 1	179.2788	7	< 0.00	All Cohort RR = 1	128.1383	7	< 0.00	All Cohort RR = 1	0.062529	7	1.00
		All Local Drifts = Net Drift	4.529437	3	0.21	All Local Drifts = Net Drift	18.65331	3	< 0.00	All Local Drifts = Net Drift	0.00076	3	1.00
Benin	Both	Net Drift = 0	138.8639	1	< 0.00	Net Drift = 0	102.5036	1	< 0.00	Net Drift = 0	0.002059	1	0.96
		All Age Deviations = 0	68.10607	1	0.00	All Age Deviations = 0	355.4018	1	< 0.00	All Age Deviations = 0	15.52679	1	< 0.00
		All Period Deviations = 0	0.096589	4	1.00	All Period Deviations = 0	0.246726	4	0.99	All Period Deviations = 0	0.012629	4	1.00
		All Cohort Deviations = 0	6.983488	6	0.32	All Cohort Deviations = 0	2.945308	6	0.82	All Cohort Deviations = 0	0.026662	6	1.00
		All Period RR = 1	140.8743	5	< 0.00	All Period RR = 1	116.0713	5	< 0.00	All Period RR = 1	0.023737	5	1.00
		All Cohort RR = 1	160.6012	7	< 0.00	All Cohort RR = 1	119.7987	7	< 0.00	All Cohort RR = 1	0.032528	7	1.00
		All Local Drifts = Net Drift	6.178344	3	0.10	All Local Drifts = Net Drift	1.49781	3	0.68	All Local Drifts = Net Drift	0.019066	3	1.00
	Male	Net Drift = 0	35.2867	1	< 0.00	Net Drift = 0	28.95303	1	< 0.00	Net Drift = 0	0.204255	1	0.65
		All Age Deviations = 0	32.74417	1	< 0.00	All Age Deviations = 0	148.5281	1	< 0.00	All Age Deviations = 0	6.168436	1	0.01
		All Period Deviations = 0	0.080348	4	1.00	All Period Deviations = 0	0.136268	4	1.00	All Period Deviations = 0	0.026718	4	1.00
		All Cohort Deviations = 0	1.598368	6	0.95	All Cohort Deviations = 0	1.567064	6	0.95	All Cohort Deviations = 0	0.206686	6	1.00
		All Period RR = 1	35.61643	5	< 0.00	All Period RR = 1	33.2702	5	< 0.00	All Period RR = 1	0.228166	5	1.00
		All Cohort RR = 1	40.46616	7	< 0.00	All Cohort RR = 1	35.10524	7	< 0.00	All Cohort RR = 1	0.269381	7	1.00
		All Local Drifts = Net Drift	1.014921	3	0.80	All Local Drifts = Net Drift	0.452914	3	0.93	All Local Drifts = Net Drift	0.184586	3	0.98
	Female	Net Drift = 0	115.1487	1	< 0.00	Net Drift = 0	77.61728	1	< 0.00	Net Drift = 0	0.01274	1	0.91
		All Age Deviations = 0	35.2203	1	< 0.00	All Age Deviations = 0	206.6186	1	< 0.00	All Age Deviations = 0	7.459257	1	< 0.01
		All Period Deviations = 0	0.240021	4	0.99	All Period Deviations = 0	0.469293	4	0.98	All Period Deviations = 0	0.094026	4	1.00
		All Cohort Deviations = 0	6.346118	6	0.39	All Cohort Deviations = 0	2.251011	6	0.90	All Cohort Deviations = 0	0.126987	6	1.00

		All Period RR = 1	116.9119	5	< 0.00	All Period RR = 1	87.21694	5	< 0.00	All Period RR = 1	0.103139	5	1.00
		All Cohort RR = 1	135.8242	7	< 0.00	All Cohort RR = 1	89.52725	7	< 0.00	All Cohort RR = 1	0.194494	7	1.00
		All Local Drifts = Net Drift	6.145753	3	0.10	All Local Drifts = Net Drift	1.835141	3	0.61	All Local Drifts = Net Drift	0.080172	3	0.99
Botswana	Both	Net Drift = 0	16.40143	1	< 0.00	Net Drift = 0	8.996906	1	< 0.00	Net Drift = 0	0.057977	1	0.81
		All Age Deviations = 0	29.57381	1	< 0.00	All Age Deviations = 0	162.5905	1	< 0.00	All Age Deviations = 0	10.13563	1	< 0.00
		All Period Deviations = 0	0.038178	4	1.00	All Period Deviations = 0	0.52835	4	0.97	All Period Deviations = 0	0.031211	4	1.00
		All Cohort Deviations = 0	6.179225	6	0.40	All Cohort Deviations = 0	3.642481	6	0.72	All Cohort Deviations = 0	0.084634	6	1.00
		All Period RR = 1	16.48154	5	< 0.01	All Period RR = 1	9.462441	5	0.09	All Period RR = 1	0.128514	5	1.00
		All Cohort RR = 1	20.86474	7	< 0.00	All Cohort RR = 1	16.95004	7	0.02	All Cohort RR = 1	0.537053	7	1.00
		All Local Drifts = Net Drift	6.120528	3	0.11	All Local Drifts = Net Drift	3.619019	3	0.31	All Local Drifts = Net Drift	0.047936	3	1.00
	Male	Net Drift = 0	8.846574	1	< 0.00	Net Drift = 0	6.547037	1	0.01	Net Drift = 0	0.074304	1	0.79
		All Age Deviations = 0	12.89799	1	< 0.00	All Age Deviations = 0	76.81125	1	< 0.00	All Age Deviations = 0	6.212373	1	0.01
		All Period Deviations = 0	0.025874	4	1.00	All Period Deviations = 0	0.181533	4	1.00	All Period Deviations = 0	0.040802	4	1.00
		All Cohort Deviations = 0	3.734121	6	0.71	All Cohort Deviations = 0	2.516312	6	0.87	All Cohort Deviations = 0	0.09792	6	1.00
		All Period RR = 1	8.871457	5	0.11	All Period RR = 1	7.013539	5	0.22	All Period RR = 1	0.182861	5	1.00
		All Cohort RR = 1	11.8456	7	0.11	All Cohort RR = 1	12.84081	7	0.08	All Cohort RR = 1	0.807021	7	1.00
		All Local Drifts = Net Drift	3.673029	3	0.30	All Local Drifts = Net Drift	2.488976	3	0.48	All Local Drifts = Net Drift	0.046362	3	1.00
	Female	Net Drift = 0	6.71465	1	0.01	Net Drift = 0	2.741289	1	0.10	Net Drift = 0	0.01289	1	0.91
		All Age Deviations = 0	17.2349	1	< 0.00	All Age Deviations = 0	85.74114	1	< 0.00	All Age Deviations = 0	2.767349	1	0.10
		All Period Deviations = 0	0.08223	4	1.00	All Period Deviations = 0	0.431784	4	0.98	All Period Deviations = 0	0.048189	4	1.00
		All Cohort Deviations = 0	2.038503	6	0.92	All Cohort Deviations = 0	1.168399	6	0.98	All Cohort Deviations = 0	0.0404	6	1.00
		All Period RR = 1	6.785288	5	0.24	All Period RR = 1	2.968041	5	0.70	All Period RR = 1	0.06033	5	1.00
		All Cohort RR = 1	7.975648	7	0.33	All Cohort RR = 1	4.92229	7	0.67	All Cohort RR = 1	0.180875	7	1.00
		All Local Drifts = Net Drift	2.02937	3	0.57	All Local Drifts = Net Drift	1.149957	3	0.77	All Local Drifts = Net Drift	0.005753	3	1.00

Burkina Faso	Both	Net Drift = 0	637.5059	1	< 0.00	Net Drift = 0	663.1965	1	< 0.00	Net Drift = 0	0.007079	1	0.93
		All Age Deviations = 0	86.10417	1	< 0.00	All Age Deviations = 0	479.2074	1	< 0.00	All Age Deviations = 0	27.57838	1	< 0.00
		All Period Deviations = 0	0.31155	4	0.99	All Period Deviations = 0	9.392759	4	0.05	All Period Deviations = 0	0.040153	4	1.00
		All Cohort Deviations = 0	19.57051	6	< 0.00	All Cohort Deviations = 0	7.482863	6	0.28	All Cohort Deviations = 0	0.349262	6	1.00
		All Period RR = 1	652.7406	5	< 0.00	All Period RR = 1	784.742	5	< 0.00	All Period RR = 1	0.040162	5	1.00
		All Cohort RR = 1	818.5964	7	< 0.00	All Cohort RR = 1	718.7496	7	< 0.00	All Cohort RR = 1	0.454119	7	1.00
		All Local Drifts = Net Drift	6.721615	3	0.08	All Local Drifts = Net Drift	4.337059	3	0.23	All Local Drifts = Net Drift	0.341555	3	0.95
	Male	Net Drift = 0	271.4005	1	< 0.00	Net Drift = 0	306.2021	1	< 0.00	Net Drift = 0	0.200861	1	0.65
		All Age Deviations = 0	40.56466	1	< 0.00	All Age Deviations = 0	199.2906	1	< 0.00	All Age Deviations = 0	15.27624	1	< 0.00
		All Period Deviations = 0	0.353479	4	0.99	All Period Deviations = 0	5.65591	4	0.23	All Period Deviations = 0	0.036915	4	1.00
		All Cohort Deviations = 0	7.10285	6	0.31	All Cohort Deviations = 0	3.365812	6	0.76	All Cohort Deviations = 0	0.077963	6	1.00
		All Period RR = 1	278.6209	5	< 0.00	All Period RR = 1	357.8636	5	< 0.00	All Period RR = 1	0.283537	5	1.00
		All Cohort RR = 1	336.4805	7	< 0.00	All Cohort RR = 1	334.3866	7	< 0.00	All Cohort RR = 1	0.397185	7	1.00
		All Local Drifts = Net Drift	2.413154	3	0.49	All Local Drifts = Net Drift	1.766413	3	0.62	All Local Drifts = Net Drift	0.043532	3	1.00
	Female	Net Drift = 0	375.616	1	< 0.00	Net Drift = 0	361.1933	1	< 0.00	Net Drift = 0	0.034984	1	0.85
		All Age Deviations = 0	43.74676	1	< 0.00	All Age Deviations = 0	282.2648	1	< 0.00	All Age Deviations = 0	12.91937	1	< 0.00
		All Period Deviations = 0	0.177237	4	1.00	All Period Deviations = 0	3.772981	4	0.44	All Period Deviations = 0	0.02845	4	1.00
		All Cohort Deviations = 0	14.10722	6	0.03	All Cohort Deviations = 0	5.513267	6	0.48	All Cohort Deviations = 0	0.339721	6	1.00
		All Period RR = 1	384.603	5	< 0.00	All Period RR = 1	432.5137	5	< 0.00	All Period RR = 1	0.10369	5	1.00
		All Cohort RR = 1	501.9952	7	< 0.00	All Cohort RR = 1	388.2485	7	< 0.00	All Cohort RR = 1	0.836467	7	1.00
		All Local Drifts = Net Drift	5.487271	3	0.14	All Local Drifts = Net Drift	4.034156	3	0.26	All Local Drifts = Net Drift	0.289828	3	0.96
Burundi	Both	Net Drift = 0	316.0823	1	< 0.00	Net Drift = 0	110.3318	1	< 0.00	Net Drift = 0	0.195269	1	0.66
		All Age Deviations = 0	56.97802	1	< 0.00	All Age Deviations = 0	467.0914	1	< 0.00	All Age Deviations = 0	31.66225	1	< 0.00
		All Period Deviations = 0	0.075903	4	1.00	All Period Deviations = 0	1.679796	4	0.79	All Period Deviations = 0	0.053045	4	1.00

		All Cohort Deviations = 0	5.996705	6	0.42	All Cohort Deviations = 0	2.454536	6	0.87	All Cohort Deviations = 0	0.049207	6	1.00
		All Period RR = 1	327.5274	5	< 0.00	All Period RR = 1	125.8048	5	< 0.00	All Period RR = 1	0.339269	5	1.00
		All Cohort RR = 1	342.4421	7	< 0.00	All Cohort RR = 1	118.1843	7	< 0.00	All Cohort RR = 1	0.813631	7	1.00
		All Local Drifts = Net Drift	5.360026	3	0.15	All Local Drifts = Net Drift	2.0617	3	0.56	All Local Drifts = Net Drift	0.040449	3	1.00
	Male	Net Drift = 0	130.101	1	< 0.00	Net Drift = 0	32.67578	1	< 0.00	Net Drift = 0	0.271022	1	0.60
		All Age Deviations = 0	30.43263	1	< 0.00	All Age Deviations = 0	219.0484	1	< 0.00	All Age Deviations = 0	20.14356	1	< 0.00
		All Period Deviations = 0	0.028556	4	1.00	All Period Deviations = 0	1.025022	4	0.91	All Period Deviations = 0	0.037943	4	1.00
		All Cohort Deviations = 0	2.53342	6	0.86	All Cohort Deviations = 0	0.460599	6	1.00	All Cohort Deviations = 0	0.00884	6	1.00
		All Period RR = 1	134.6534	5	< 0.00	All Period RR = 1	40.45849	5	< 0.00	All Period RR = 1	0.331567	5	1.00
		All Cohort RR = 1	140.0826	7	< 0.00	All Cohort RR = 1	36.7874	7	< 0.00	All Cohort RR = 1	0.8792	7	1.00
		All Local Drifts = Net Drift	2.370638	3	0.50	All Local Drifts = Net Drift	0.38322	3	0.94	All Local Drifts = Net Drift	0.001124	3	1.00
	Female	Net Drift = 0	189.5543	1	< 0.00	Net Drift = 0	82.81494	1	< 0.00	Net Drift = 0	0.0897	1	0.76
		All Age Deviations = 0	27.29348	1	< 0.00	All Age Deviations = 0	248.9783	1	< 0.00	All Age Deviations = 0	12.27751	1	< 0.00
		All Period Deviations = 0	0.039534	4	1.00	All Period Deviations = 0	1.773952	4	0.78	All Period Deviations = 0	0.122925	4	1.00
		All Cohort Deviations = 0	3.920684	6	0.69	All Cohort Deviations = 0	3.336084	6	0.77	All Cohort Deviations = 0	0.09855	6	1.00
		All Period RR = 1	196.5372	5	< 0.00	All Period RR = 1	90.41075	5	< 0.00	All Period RR = 1	0.340671	5	1.00
		All Cohort RR = 1	208.5802	7	< 0.00	All Cohort RR = 1	87.09758	7	< 0.00	All Cohort RR = 1	0.454896	7	1.00
		All Local Drifts = Net Drift	3.327239	3	0.34	All Local Drifts = Net Drift	2.90864	3	0.41	All Local Drifts = Net Drift	0.021964	3	1.00
Cabo Verde	Both	Net Drift = 0	1.948811	1	0.16	Net Drift = 0	1.956724	1	0.16	Net Drift = 0	0.002573	1	0.96
		All Age Deviations = 0	4.958552	1	0.03	All Age Deviations = 0	33.71464	1	< 0.00	All Age Deviations = 0	0.543973	1	0.46
		All Period Deviations = 0	0.009336	4	1.00	All Period Deviations = 0	0.244793	4	0.99	All Period Deviations = 0	0.074112	4	1.00
		All Cohort Deviations = 0	0.437441	6	1.00	All Cohort Deviations = 0	0.407701	6	1.00	All Cohort Deviations = 0	0.077741	6	1.00
		All Period RR = 1	1.965351	5	0.85	All Period RR = 1	2.009802	5	0.85	All Period RR = 1	0.074387	5	1.00
		All Cohort RR = 1	2.264219	7	0.94	All Cohort RR = 1	2.911798	7	0.89	All Cohort RR = 1	0.102834	7	1.00

		All Local Drifts = Net Drift	0.363569	3	0.95	All Local Drifts = Net Drift	0.359139	3	0.95	All Local Drifts = Net Drift	0.026972	3	1.00
	Male	Net Drift = 0	0.571007	1	0.45	Net Drift = 0	0.742472	1	0.39	Net Drift = 0	0.027228	1	0.87
		All Age Deviations = 0	2.660917	1	0.10	All Age Deviations = 0	17.79658	1	< 0.00	All Age Deviations = 0	0.721076	1	0.40
		All Period Deviations = 0	0.014145	4	1.00	All Period Deviations = 0	0.286299	4	0.99	All Period Deviations = 0	0.339697	4	0.99
		All Cohort Deviations = 0	0.196779	6	1.00	All Cohort Deviations = 0	0.399573	6	1.00	All Cohort Deviations = 0	0.547171	6	1.00
		All Period RR = 1	0.593651	5	0.99	All Period RR = 1	0.845633	5	0.97	All Period RR = 1	0.521346	5	0.99
		All Cohort RR = 1	0.775143	7	1.00	All Cohort RR = 1	1.316378	7	0.99	All Cohort RR = 1	0.709681	7	1.00
		All Local Drifts = Net Drift	0.133904	3	0.99	All Local Drifts = Net Drift	0.384162	3	0.94	All Local Drifts = Net Drift	0.271378	3	0.97
	Female	Net Drift = 0	1.200005	1	0.27	Net Drift = 0	1.030966	1	0.31	Net Drift = 0	0.001485	1	0.97
		All Age Deviations = 0	3.45478	1	0.06	All Age Deviations = 0	17.64719	1	< 0.00	All Age Deviations = 0	0.998538	1	0.32
		All Period Deviations = 0	0.020586	4	1.00	All Period Deviations = 0	0.028766	4	1.00	All Period Deviations = 0	0.171608	4	1.00
		All Cohort Deviations = 0	0.269029	6	1.00	All Cohort Deviations = 0	0.160269	6	1.00	All Cohort Deviations = 0	0.204375	6	1.00
		All Period RR = 1	1.227208	5	0.94	All Period RR = 1	1.170865	5	0.95	All Period RR = 1	0.179026	5	1.00
		All Cohort RR = 1	1.474235	7	0.98	All Cohort RR = 1	1.608881	7	0.98	All Cohort RR = 1	0.212433	7	1.00
		All Local Drifts = Net Drift	0.146051	3	0.99	All Local Drifts = Net Drift	0.136873	3	0.99	All Local Drifts = Net Drift	0.133348	3	0.99
Cameroon	Both	Net Drift = 0	80.26209	1	< 0.00	Net Drift = 0	64.88878	1	< 0.00	Net Drift = 0	0.012182	1	0.91
		All Age Deviations = 0	208.8595	1	< 0.00	All Age Deviations = 0	1127.814	1	< 0.00	All Age Deviations = 0	34.21313	1	< 0.00
		All Period Deviations = 0	0.054042	4	1.00	All Period Deviations = 0	0.719589	4	0.95	All Period Deviations = 0	0.112354	4	1.00
		All Cohort Deviations = 0	4.938281	6	0.55	All Cohort Deviations = 0	5.945201	6	0.43	All Cohort Deviations = 0	0.042756	6	1.00
		All Period RR = 1	81.35391	5	< 0.00	All Period RR = 1	72.44319	5	< 0.00	All Period RR = 1	0.114074	5	1.00
		All Cohort RR = 1	83.0947	7	< 0.00	All Cohort RR = 1	76.27854	7	< 0.00	All Cohort RR = 1	0.105179	7	1.00
		All Local Drifts = Net Drift	3.714698	3	0.29	All Local Drifts = Net Drift	4.346165	3	0.23	All Local Drifts = Net Drift	0.010706	3	1.00
	Male	Net Drift = 0	23.30876	1	< 0.00	Net Drift = 0	24.23308	1	< 0.00	Net Drift = 0	0.102029	1	0.75
		All Age Deviations = 0	99.19362	1	< 0.00	All Age Deviations = 0	467.6984	1	< 0.00	All Age Deviations = 0	16.69593	1	< 0.00

		All Period Deviations = 0	0.046927	4	1.00	All Period Deviations = 0	0.336328	4	0.99	All Period Deviations = 0	0.02105	4	1.00
		All Cohort Deviations = 0	2.40455	6	0.88	All Cohort Deviations = 0	3.427643	6	0.75	All Cohort Deviations = 0	0.032882	6	1.00
		All Period RR = 1	23.77452	5	< 0.00	All Period RR = 1	27.04896	5	< 0.00	All Period RR = 1	0.193886	5	1.00
		All Cohort RR = 1	24.26294	7	< 0.00	All Cohort RR = 1	30.55998	7	< 0.00	All Cohort RR = 1	0.199769	7	1.00
		All Local Drifts = Net Drift	1.703808	3	0.64	All Local Drifts = Net Drift	2.637077	3	0.45	All Local Drifts = Net Drift	0.014333	3	1.00
	Female	Net Drift = 0	58.8645	1	< 0.00	Net Drift = 0	41.70188	1	< 0.00	Net Drift = 0	0.099569	1	0.75
		All Age Deviations = 0	113.2574	1	< 0.00	All Age Deviations = 0	662.9001	1	< 0.00	All Age Deviations = 0	18.66532	1	< 0.00
		All Period Deviations = 0	0.32906	4	0.99	All Period Deviations = 0	0.566651	4	0.97	All Period Deviations = 0	0.104535	4	1.00
		All Cohort Deviations = 0	2.304	6	0.89	All Cohort Deviations = 0	2.558607	6	0.86	All Cohort Deviations = 0	0.223765	6	1.00
		All Period RR = 1	59.43088	5	< 0.00	All Period RR = 1	46.47789	5	< 0.00	All Period RR = 1	0.22613	5	1.00
		All Cohort RR = 1	61.77622	7	< 0.00	All Cohort RR = 1	46.52817	7	< 0.00	All Cohort RR = 1	0.573379	7	1.00
		All Local Drifts = Net Drift	1.788629	3	0.62	All Local Drifts = Net Drift	1.783809	3	< 0.62	All Local Drifts = Net Drift	0.187814	3	0.98
Central African Republic	Both	Net Drift = 0	39.8809	1	< 0.00	Net Drift = 0	27.82956	1	< 0.00	Net Drift = 0	0.004966	1	0.94
		All Age Deviations = 0	25.41516	1	< 0.00	All Age Deviations = 0	389.5946	1	< 0.00	All Age Deviations = 0	14.53969	1	< 0.00
		All Period Deviations = 0	0.035274	4	1.00	All Period Deviations = 0	0.223009	4	0.99	All Period Deviations = 0	0.03355	4	1.00
		All Cohort Deviations = 0	2.262923	6	0.89	All Cohort Deviations = 0	6.701182	6	0.35	All Cohort Deviations = 0	0.116558	6	1.00
		All Period RR = 1	40.54186	5	< 0.00	All Period RR = 1	31.06692	5	< 0.00	All Period RR = 1	0.039869	5	1.00
		All Cohort RR = 1	44.486	7	< 0.00	All Cohort RR = 1	28.20681	7	< 0.00	All Cohort RR = 1	0.348305	7	1.00
		All Local Drifts = Net Drift	2.247725	3	0.52	All Local Drifts = Net Drift	6.584831	3	0.09	All Local Drifts = Net Drift	0.111601	3	0.99
	Male	Net Drift = 0	11.95984	1	< 0.00	Net Drift = 0	6.881127	1	0.01	Net Drift = 0	0.064171	1	0.80
		All Age Deviations = 0	14.88324	1	< 0.00	All Age Deviations = 0	213.5318	1	< 0.00	All Age Deviations = 0	4.98356	1	0.03
		All Period Deviations = 0	0.052318	4	1.00	All Period Deviations = 0	0.081285	4	1.00	All Period Deviations = 0	0.002838	4	1.00
		All Cohort Deviations = 0	1.004324	6	0.99	All Cohort Deviations = 0	3.25907	6	0.78	All Cohort Deviations = 0	0.054703	6	1.00
		All Period RR = 1	12.24836	5	0.03	All Period RR = 1	7.833509	5	0.17	All Period RR = 1	0.10081	5	1.00

		All Cohort RR = 1	13.60916	7	0.06	All Cohort RR = 1	7.382448	7	0.39	All Cohort RR = 1	0.1016	7	1.00
		All Local Drifts = Net Drift	0.99583	3	0.80	All Local Drifts = Net Drift	3.198552	3	0.36	All Local Drifts = Net Drift	0.037593	3	1.00
	Female	Net Drift = 0	35.61085	1	< 0.00	Net Drift = 0	26.06871	1	< 0.00	Net Drift = 0	0.078882	1	0.78
		All Age Deviations = 0	10.40427	1	< 0.00	All Age Deviations = 0	176.671	1	< 0.00	All Age Deviations = 0	5.676005	1	0.02
		All Period Deviations = 0	0.039328	4	1.00	All Period Deviations = 0	0.167945	4	1.00	All Period Deviations = 0	0.085704	4	1.00
		All Cohort Deviations = 0	0.62621	6	1.00	All Cohort Deviations = 0	2.943585	6	0.82	All Cohort Deviations = 0	0.164058	6	1.00
		All Period RR = 1	36.34441	5	< 0.00	All Period RR = 1	29.21955	5	< 0.00	All Period RR = 1	0.158975	5	1.00
		All Cohort RR = 1	38.74485	7	< 0.00	All Cohort RR = 1	26.66389	7	< 0.00	All Cohort RR = 1	0.240622	7	1.00
		All Local Drifts = Net Drift	0.615759	3	0.89	All Local Drifts = Net Drift	2.907779	3	0.41	All Local Drifts = Net Drift	0.087744	3	0.99
Chad	Both	Net Drift = 0	238.9728	1	< 0.00	Net Drift = 0	205.8222	1	< 0.00	Net Drift = 0	0.006817	1	0.93
		All Age Deviations = 0	72.98967	1	< 0.00	All Age Deviations = 0	577.0928	1	< 0.00	All Age Deviations = 0	19.71527	1	< 0.00
		All Period Deviations = 0	1.14483	4	0.89	All Period Deviations = 0	3.228522	4	0.52	All Period Deviations = 0	0.036605	4	1.00
		All Cohort Deviations = 0	7.022392	6	0.32	All Cohort Deviations = 0	1.112	6	0.98	All Cohort Deviations = 0	0.113595	6	1.00
		All Period RR = 1	250.5872	5	< 0.00	All Period RR = 1	223.9769	5	< 0.00	All Period RR = 1	0.04339	5	1.00
		All Cohort RR = 1	281.1369	7	< 0.00	All Cohort RR = 1	217.0572	7	< 0.00	All Cohort RR = 1	0.193836	7	1.00
		All Local Drifts = Net Drift	6.033644	3	0.11	All Local Drifts = Net Drift	0.975695	3	0.81	All Local Drifts = Net Drift	0.10831	3	0.99
	Male	Net Drift = 0	70.6954	1	< 0.00	Net Drift = 0	62.12278	1	< 0.00	Net Drift = 0	0.028397	1	0.87
		All Age Deviations = 0	41.25217	1	< 0.00	All Age Deviations = 0	264.4368	1	< 0.00	All Age Deviations = 0	9.364306	1	< 0.00
		All Period Deviations = 0	1.007354	4	0.91	All Period Deviations = 0	3.116992	4	0.54	All Period Deviations = 0	0.007414	4	1.00
		All Cohort Deviations = 0	2.417097	6	0.88	All Cohort Deviations = 0	0.645292	6	1.00	All Cohort Deviations = 0	0.043322	6	1.00
		All Period RR = 1	75.87675	5	< 0.00	All Period RR = 1	64.57263	5	< 0.00	All Period RR = 1	0.028891	5	1.00
		All Cohort RR = 1	79.44368	7	< 0.00	All Cohort RR = 1	65.44303	7	< 0.00	All Cohort RR = 1	0.060641	7	1.00
		All Local Drifts = Net Drift	2.339415	3	0.51	All Local Drifts = Net Drift	0.539427	3	0.91	All Local Drifts = Net Drift	0.015689	3	1.00
	Female	Net Drift = 0	187.2373	1	< 0.00	Net Drift = 0	153.8408	1	< 0.00	Net Drift = 0	0.079504	1	0.78

		All Age Deviations = 0	33.20626	1	< 0.00	All Age Deviations = 0	313.1338	1	< 0.00	All Age Deviations = 0	10.48085	1	< 0.00
		All Period Deviations = 0	0.449367	4	0.98	All Period Deviations = 0	0.845351	4	0.93	All Period Deviations = 0	0.069127	4	1.00
		All Cohort Deviations = 0	6.961071	6	0.32	All Cohort Deviations = 0	3.386123	6	0.76	All Cohort Deviations = 0	0.073167	6	1.00
		All Period RR = 1	194.142	5	< 0.00	All Period RR = 1	174.9225	5	< 0.00	All Period RR = 1	0.157406	5	1.00
		All Cohort RR = 1	235.4554	7	< 0.00	All Cohort RR = 1	164.5035	7	< 0.00	All Cohort RR = 1	0.354109	7	1.00
		All Local Drifts = Net Drift	5.356691	3	0.15	All Local Drifts = Net Drift	3.037762	3	0.39	All Local Drifts = Net Drift	0.066082	3	1.00
Comoros	Both	Net Drift = 0	7.781409	1	0.01	Net Drift = 0	2.988157	1	0.08	Net Drift = 0	0.005298	1	0.94
		All Age Deviations = 0	4.436786	1	0.04	All Age Deviations = 0	30.2702	1	< 0.00	All Age Deviations = 0	2.657056	1	0.10
		All Period Deviations = 0	0.020442	4	1.00	All Period Deviations = 0	0.05431	4	1.00	All Period Deviations = 0	0.078254	4	1.00
		All Cohort Deviations = 0	0.024344	6	1.00	All Cohort Deviations = 0	0.235127	6	1.00	All Cohort Deviations = 0	0.183302	6	1.00
		All Period RR = 1	8.028427	5	0.15	All Period RR = 1	3.454312	5	0.63	All Period RR = 1	0.085465	5	1.00
		All Cohort RR = 1	8.184554	7	0.32	All Cohort RR = 1	3.297361	7	0.86	All Cohort RR = 1	0.193936	7	1.00
		All Local Drifts = Net Drift	0.021063	3	1.00	All Local Drifts = Net Drift	0.229246	3	0.97	All Local Drifts = Net Drift	0.018946	3	1.00
	Male	Net Drift = 0	2.731479	1	0.10	Net Drift = 0	0.547306	1	0.46	Net Drift = 0	0.040286	1	0.84
		All Age Deviations = 0	2.278	1	0.13	All Age Deviations = 0	14.37831	1	< 0.00	All Age Deviations = 0	1.835905	1	0.18
		All Period Deviations = 0	0.007533	4	1.00	All Period Deviations = 0	0.039989	4	1.00	All Period Deviations = 0	0.083938	4	1.00
		All Cohort Deviations = 0	0.064589	6	1.00	All Cohort Deviations = 0	0.107305	6	1.00	All Cohort Deviations = 0	0.11836	6	1.00
		All Period RR = 1	2.764268	5	0.74	All Period RR = 1	0.716307	5	0.98	All Period RR = 1	0.102414	5	1.00
		All Cohort RR = 1	2.81388	7	0.90	All Cohort RR = 1	0.598318	7	1.00	All Cohort RR = 1	0.153409	7	1.00
		All Local Drifts = Net Drift	0.033278	3	1.00	All Local Drifts = Net Drift	0.100029	3	0.99	All Local Drifts = Net Drift	0.090843	3	0.99
	Female	Net Drift = 0	5.533056	1	0.02	Net Drift = 0	2.995534	1	0.08	Net Drift = 0	0.052117	1	0.82
		All Age Deviations = 0	2.599066	1	0.11	All Age Deviations = 0	16.16677	1	< 0.00	All Age Deviations = 0	0.926582	1	0.34
		All Period Deviations = 0	0.028972	4	1.00	All Period Deviations = 0	0.02841	4	1.00	All Period Deviations = 0	0.000975	4	1.00
		All Cohort Deviations = 0	0.064713	6	1.00	All Cohort Deviations = 0	0.124469	6	1.00	All Cohort Deviations = 0	0.004539	6	1.00

		All Period RR = 1	5.761359	5	0.33	All Period RR = 1	3.351887	5	0.65	All Period RR = 1	0.067044	5	1.00
		All Cohort RR = 1	5.95761	7	0.54	All Cohort RR = 1	3.42701	7	0.84	All Cohort RR = 1	0.240029	7	1.00
		All Local Drifts = Net Drift	0.054059	3	1.00	All Local Drifts = Net Drift	0.113621	3	0.99	All Local Drifts = Net Drift	0.004397	3	1.00
Congo	Both	Net Drift = 0	4.76537	1	0.03	Net Drift = 0	3.121571	1	0.08	Net Drift = 0	0.192156	1	0.66
		All Age Deviations = 0	42.01046	1	< 0.00	All Age Deviations = 0	516.4712	1	< 0.00	All Age Deviations = 0	10.84541	1	< 0.00
		All Period Deviations = 0	0.336382	4	0.99	All Period Deviations = 0	11.74538	4	0.02	All Period Deviations = 0	0.014692	4	1.00
		All Cohort Deviations = 0	1.26976	6	0.97	All Cohort Deviations = 0	8.798389	6	0.19	All Cohort Deviations = 0	0.132349	6	1.00
		All Period RR = 1	5.491749	5	0.36	All Period RR = 1	19.45176	5	< 0.00	All Period RR = 1	0.197844	5	1.00
		All Cohort RR = 1	6.031069	7	0.54	All Cohort RR = 1	21.6966	7	< 0.00	All Cohort RR = 1	0.241043	7	1.00
		All Local Drifts = Net Drift	1.226507	3	0.75	All Local Drifts = Net Drift	8.763169	3	0.03	All Local Drifts = Net Drift	0.116328	3	0.99
	Male	Net Drift = 0	0.731273	1	0.39	Net Drift = 0	5.672836	1	0.02	Net Drift = 0	0.039791	1	0.84
		All Age Deviations = 0	21.23057	1	0.00	All Age Deviations = 0	249.0797	1	< 0.00	All Age Deviations = 0	3.533358	1	0.06
		All Period Deviations = 0	0.155404	4	1.00	All Period Deviations = 0	3.273251	4	0.51	All Period Deviations = 0	0.08822	4	1.00
		All Cohort Deviations = 0	0.668147	6	1.00	All Cohort Deviations = 0	6.179449	6	0.40	All Cohort Deviations = 0	0.239622	6	1.00
		All Period RR = 1	0.986905	5	0.96	All Period RR = 1	11.91587	5	0.04	All Period RR = 1	0.141304	5	1.00
		All Cohort RR = 1	1.321065	7	0.99	All Cohort RR = 1	23.5149	7	< 0.00	All Cohort RR = 1	0.320445	7	1.00
		All Local Drifts = Net Drift	0.644074	3	0.89	All Local Drifts = Net Drift	6.128354	3	0.11	All Local Drifts = Net Drift	0.208287	3	0.98
	Female	Net Drift = 0	5.842332	1	0.02	Net Drift = 0	0.001705	1	0.97	Net Drift = 0	0.094222	1	0.76
		All Age Deviations = 0	21.79392	1	< 0.00	All Age Deviations = 0	268.6267	1	< 0.00	All Age Deviations = 0	4.066335	1	0.04
		All Period Deviations = 0	0.165508	4	1.00	All Period Deviations = 0	9.554252	4	0.05	All Period Deviations = 0	0.134527	4	1.00
		All Cohort Deviations = 0	0.492333	6	1.00	All Cohort Deviations = 0	3.07538	6	0.80	All Cohort Deviations = 0	0.111726	6	1.00
		All Period RR = 1	6.32128	5	0.28	All Period RR = 1	10.23862	5	0.07	All Period RR = 1	0.150918	5	1.00
		All Cohort RR = 1	6.493327	7	0.48	All Cohort RR = 1	3.758291	7	0.81	All Cohort RR = 1	0.152435	7	1.00
		All Local Drifts = Net Drift	0.432661	3	0.93	All Local Drifts = Net Drift	3.069966	3	0.38	All Local Drifts = Net Drift	0.085611	3	0.99

Cote d'Ivoire	Both	Net Drift = 0	201.9875	1	< 0.00	Net Drift = 0	102.3389	1	< 0.00	Net Drift = 0	0.00466	1	0.95
		All Age Deviations = 0	145.875	1	< 0.00	All Age Deviations = 0	693.2121	1	< 0.00	All Age Deviations = 0	27.07726	1	< 0.00
		All Period Deviations = 0	0.54562	4	0.97	All Period Deviations = 0	1.720997	4	0.79	All Period Deviations = 0	0.03799	4	1.00
		All Cohort Deviations = 0	1.237711	6	0.97	All Cohort Deviations = 0	5.21955	6	0.52	All Cohort Deviations = 0	0.148652	6	1.00
		All Period RR = 1	206.1205	5	< 0.00	All Period RR = 1	112.1964	5	< 0.00	All Period RR = 1	0.038805	5	1.00
		All Cohort RR = 1	219.5884	7	< 0.00	All Cohort RR = 1	103.4874	7	< 0.00	All Cohort RR = 1	0.149321	7	1.00
		All Local Drifts = Net Drift	1.093561	3	0.78	All Local Drifts = Net Drift	5.199566	3	0.16	All Local Drifts = Net Drift	0.134001	3	0.99
	Male	Net Drift = 0	75.40107	1	< 0.00	Net Drift = 0	32.95782	1	< 0.00	Net Drift = 0	0.033498	1	0.85
		All Age Deviations = 0	78.77066	1	< 0.00	All Age Deviations = 0	299.8285	1	< 0.00	All Age Deviations = 0	12.54461	1	< 0.00
		All Period Deviations = 0	0.399557	4	0.98	All Period Deviations = 0	0.687024	4	0.95	All Period Deviations = 0	0.026352	4	1.00
		All Cohort Deviations = 0	0.099533	6	1.00	All Cohort Deviations = 0	0.577819	6	1.00	All Cohort Deviations = 0	0.123485	6	1.00
		All Period RR = 1	78.04004	5	< 0.00	All Period RR = 1	39.12213	5	< 0.00	All Period RR = 1	0.05942	5	1.00
		All Cohort RR = 1	79.46006	7	< 0.00	All Cohort RR = 1	34.48239	7	< 0.00	All Cohort RR = 1	0.521877	7	1.00
		All Local Drifts = Net Drift	0.087305	3	0.99	All Local Drifts = Net Drift	0.49254	3	0.92	All Local Drifts = Net Drift	0.119171	3	0.99
	Female	Net Drift = 0	131.1237	1	< 0.00	Net Drift = 0	73.70554	1	< 0.00	Net Drift = 0	0.025211	1	0.87
		All Age Deviations = 0	74.35716	1	< 0.00	All Age Deviations = 0	398.8177	1	< 0.00	All Age Deviations = 0	14.51887	1	< 0.00
		All Period Deviations = 0	0.223512	4	0.99	All Period Deviations = 0	2.837305	4	0.59	All Period Deviations = 0	0.05217	4	1.00
		All Cohort Deviations = 0	1.503911	6	0.96	All Cohort Deviations = 0	6.622747	6	0.36	All Cohort Deviations = 0	0.194726	6	1.00
		All Period RR = 1	132.5937	5	< 0.00	All Period RR = 1	78.28969	5	< 0.00	All Period RR = 1	0.110098	5	1.00
		All Cohort RR = 1	150.2818	7	< 0.00	All Cohort RR = 1	74.12695	7	< 0.00	All Cohort RR = 1	0.549263	7	1.00
		All Local Drifts = Net Drift	1.437699	3	0.70	All Local Drifts = Net Drift	6.494501	3	0.09	All Local Drifts = Net Drift	0.185962	3	0.98
Democratic Republic of the Congo	Both	Net Drift = 0	1228.71	1	< 0.00	Net Drift = 0	1042.528	1	< 0.00	Net Drift = 0	0.434469	1	0.51
		All Age Deviations = 0	704.7417	1	< 0.00	All Age Deviations = 0	6995.956	1	< 0.00	All Age Deviations = 0	188.8933	1	< 0.00
		All Period Deviations = 0	4.087241	4	0.39	All Period Deviations = 0	2.307974	4	0.68	All Period Deviations = 0	0.094478	4	1.00

		All Cohort Deviations = 0	14.17364	6	0.03	All Cohort Deviations = 0	45.11686	6	< 0.00	All Cohort Deviations = 0	0.069079	6	1.00
		All Period RR = 1	1274.738	5	< 0.00	All Period RR = 1	1272.34	5	< 0.00	All Period RR = 1	0.584332	5	0.99
		All Cohort RR = 1	1340.77	7	< 0.00	All Cohort RR = 1	1137.903	7	< 0.00	All Cohort RR = 1	1.144022	7	0.99
		All Local Drifts = Net Drift	13.35272	3	< 0.00	All Local Drifts = Net Drift	44.59552	3	< 0.00	All Local Drifts = Net Drift	0.05821	3	1.00
	Male	Net Drift = 0	361.5213	1	< 0.00	Net Drift = 0	265.7144	1	< 0.00	Net Drift = 0	0.214097	1	0.64
		All Age Deviations = 0	372.5401	1	< 0.00	All Age Deviations = 0	3484.237	1	< 0.00	All Age Deviations = 0	94.54619	1	< 0.00
		All Period Deviations = 0	2.845013	4	0.58	All Period Deviations = 0	4.655102	4	0.32	All Period Deviations = 0	0.174183	4	1.00
		All Cohort Deviations = 0	3.198397	6	0.78	All Cohort Deviations = 0	13.84787	6	0.03	All Cohort Deviations = 0	0.08818	6	1.00
		All Period RR = 1	378.1919	5	< 0.00	All Period RR = 1	354.7721	5	< 0.00	All Period RR = 1	0.300138	5	1.00
		All Cohort RR = 1	385.5992	7	< 0.00	All Cohort RR = 1	287.0634	7	< 0.00	All Cohort RR = 1	0.528549	7	1.00
		All Local Drifts = Net Drift	3.12814	3	0.37	All Local Drifts = Net Drift	13.47264	3	< 0.00	All Local Drifts = Net Drift	0.036758	3	1.00
	Female	Net Drift = 0	983.2464	1	< 0.00	Net Drift = 0	885.2681	1	< 0.00	Net Drift = 0	0.125639	1	0.72
		All Age Deviations = 0	347.6635	1	< 0.00	All Age Deviations = 0	3544.705	1	< 0.00	All Age Deviations = 0	95.41541	1	< 0.00
		All Period Deviations = 0	2.670505	4	0.61	All Period Deviations = 0	1.718569	4	0.79	All Period Deviations = 0	0.056722	4	1.00
		All Cohort Deviations = 0	13.33295	6	0.04	All Cohort Deviations = 0	29.99454	6	< 0.00	All Cohort Deviations = 0	0.087173	6	1.00
		All Period RR = 1	1021.027	5	< 0.00	All Period RR = 1	1037.59	5	< 0.00	All Period RR = 1	0.300743	5	1.00
		All Cohort RR = 1	1104.788	7	< 0.00	All Cohort RR = 1	973.6938	7	< 0.00	All Cohort RR = 1	0.794642	7	1.00
		All Local Drifts = Net Drift	11.68629	3	0.01	All Local Drifts = Net Drift	29.7231	3	< 0.00	All Local Drifts = Net Drift	0.081356	3	0.99
Djibouti	Both	Net Drift = 0	30.4418	1	< 0.00	Net Drift = 0	22.31276	1	< 0.00	Net Drift = 0	0.046669	1	0.83
		All Age Deviations = 0	3.895001	1	0.05	All Age Deviations = 0	30.58172	1	< 0.00	All Age Deviations = 0	2.929617	1	0.09
		All Period Deviations = 0	0.036726	4	1.00	All Period Deviations = 0	0.029147	4	1.00	All Period Deviations = 0	0.108648	4	1.00
		All Cohort Deviations = 0	1.110938	6	0.98	All Cohort Deviations = 0	0.663539	6	1.00	All Cohort Deviations = 0	0.111869	6	1.00
		All Period RR = 1	32.46567	5	< 0.00	All Period RR = 1	26.66637	5	< 0.00	All Period RR = 1	0.129416	5	1.00
		All Cohort RR = 1	33.95621	7	< 0.00	All Cohort RR = 1	26.11086	7	< 0.00	All Cohort RR = 1	0.137195	7	1.00

		All Local Drifts = Net Drift	0.972799	3	0.81	All Local Drifts = Net Drift	0.647035	3	0.89	All Local Drifts = Net Drift	0.054259	3	1.00
	Male	Net Drift = 0	14.62391	1	< 0.00	Net Drift = 0	10.68714	1	< 0.00	Net Drift = 0	0.022931	1	0.88
		All Age Deviations = 0	2.820986	1	0.09	All Age Deviations = 0	15.95776	1	< 0.00	All Age Deviations = 0	2.038697	1	0.15
		All Period Deviations = 0	0.037909	4	1.00	All Period Deviations = 0	0.010626	4	1.00	All Period Deviations = 0	0.095864	4	1.00
		All Cohort Deviations = 0	0.565197	6	1.00	All Cohort Deviations = 0	0.198033	6	1.00	All Cohort Deviations = 0	0.096697	6	1.00
		All Period RR = 1	15.45531	5	0.01	All Period RR = 1	12.99701	5	0.02	All Period RR = 1	0.110933	5	1.00
		All Cohort RR = 1	16.25778	7	0.02	All Cohort RR = 1	12.5968	7	0.08	All Cohort RR = 1	0.09971	7	1.00
		All Local Drifts = Net Drift	0.555985	3	0.91	All Local Drifts = Net Drift	0.186762	3	0.98	All Local Drifts = Net Drift	0.021974	3	1.00
	Female	Net Drift = 0	15.73936	1	< 0.00	Net Drift = 0	11.06536	1	< 0.00	Net Drift = 0	0.14888	1	0.70
		All Age Deviations = 0	1.686828	1	0.19	All Age Deviations = 0	14.88664	1	< 0.00	All Age Deviations = 0	0.619946	1	0.43
		All Period Deviations = 0	0.009582	4	1.00	All Period Deviations = 0	0.009022	4	1.00	All Period Deviations = 0	0.069331	4	1.00
		All Cohort Deviations = 0	0.718114	6	0.99	All Cohort Deviations = 0	0.473639	6	1.00	All Cohort Deviations = 0	0.076465	6	1.00
		All Period RR = 1	16.80445	5	< 0.00	All Period RR = 1	12.97856	5	0.02	All Period RR = 1	0.197498	5	1.00
		All Cohort RR = 1	17.82185	7	0.01	All Cohort RR = 1	12.52804	7	0.08	All Cohort RR = 1	0.323286	7	1.00
		All Local Drifts = Net Drift	0.597107	3	0.90	All Local Drifts = Net Drift	0.431463	3	0.93	All Local Drifts = Net Drift	0.058205	3	1.00
Egypt	Both	Net Drift = 0	371.9595	1	< 0.00	Net Drift = 0	458.9158	1	< 0.00	Net Drift = 0	0.194876	1	0.66
		All Age Deviations = 0	218.5403	1	< 0.00	All Age Deviations = 0	1705.036	1	< 0.00	All Age Deviations = 0	66.50392	1	< 0.00
		All Period Deviations = 0	39.03473	4	< 0.00	All Period Deviations = 0	11.59959	4	0.02	All Period Deviations = 0	0.099954	4	1.00
		All Cohort Deviations = 0	42.31339	6	< 0.00	All Cohort Deviations = 0	51.36506	6	< 0.00	All Cohort Deviations = 0	0.05956	6	1.00
		All Period RR = 1	415.6321	5	< 0.00	All Period RR = 1	520.8648	5	< 0.00	All Period RR = 1	0.279052	5	1.00
		All Cohort RR = 1	440.7856	7	< 0.00	All Cohort RR = 1	619.2812	7	< 0.00	All Cohort RR = 1	0.978094	7	1.00
		All Local Drifts = Net Drift	35.62575	3	< 0.00	All Local Drifts = Net Drift	45.31387	3	< 0.00	All Local Drifts = Net Drift	0.054413	3	1.00
	Male	Net Drift = 0	134.6929	1	< 0.00	Net Drift = 0	96.9058	1	< 0.00	Net Drift = 0	0.222106	1	0.64
		All Age Deviations = 0	167.0036	1	< 0.00	All Age Deviations = 0	1043.823	1	< 0.00	All Age Deviations = 0	32.27641	1	< 0.00

		All Period Deviations = 0	39.49124	4	< 0.00	All Period Deviations = 0	15.85868	4	< 0.00	All Period Deviations = 0	0.019929	4	1.00
		All Cohort Deviations = 0	21.51067	6	< 0.00	All Cohort Deviations = 0	33.14912	6	< 0.00	All Cohort Deviations = 0	0.063961	6	1.00
		All Period RR = 1	169.9782	5	< 0.00	All Period RR = 1	136.086	5	< 0.00	All Period RR = 1	0.239442	5	1.00
		All Cohort RR = 1	167.0109	7	< 0.00	All Cohort RR = 1	169.5662	7	< 0.00	All Cohort RR = 1	0.936053	7	1.00
		All Local Drifts = Net Drift	15.993	3	< 0.00	All Local Drifts = Net Drift	28.59551	3	< 0.00	All Local Drifts = Net Drift	0.05866	3	1.00
	Female	Net Drift = 0	830.5886	1	< 0.00	Net Drift = 0	1196.581	1	< 0.00	Net Drift = 0	0.093064	1	0.76
		All Age Deviations = 0	288.0592	1	< 0.00	All Age Deviations = 0	2518.13	1	< 0.00	All Age Deviations = 0	34.29563	1	< 0.00
		All Period Deviations = 0	28.73117	4	< 0.00	All Period Deviations = 0	16.32162	4	< 0.00	All Period Deviations = 0	0.182538	4	1.00
		All Cohort Deviations = 0	73.93589	6	< 0.00	All Cohort Deviations = 0	73.43339	6	< 0.00	All Cohort Deviations = 0	0.09039	6	1.00
		All Period RR = 1	887.8964	5	< 0.00	All Period RR = 1	1292.722	5	< 0.00	All Period RR = 1	0.241719	5	1.00
		All Cohort RR = 1	967.4895	7	< 0.00	All Cohort RR = 1	1483.673	7	3.0208451240494e-316	All Cohort RR = 1	0.289341	7	1.00
		All Local Drifts = Net Drift	65.12487	3	< 0.00	All Local Drifts = Net Drift	65.43114	3	< 0.00	All Local Drifts = Net Drift	0.078566	3	0.99
Equatorial Guinea	Both	Net Drift = 0	24.24445	1	< 0.00	Net Drift = 0	21.5901	1	< 0.00	Net Drift = 0	0.07652	1	0.78
		All Age Deviations = 0	10.39372	1	< 0.00	All Age Deviations = 0	114.407	1	0.00	All Age Deviations = 0	3.303785	1	0.07
		All Period Deviations = 0	0.012008	4	1.00	All Period Deviations = 0	0.094727	4	1.00	All Period Deviations = 0	0.086657	4	1.00
		All Cohort Deviations = 0	0.786828	6	0.99	All Cohort Deviations = 0	0.473308	6	1.00	All Cohort Deviations = 0	0.137982	6	1.00
		All Period RR = 1	25.75338	5	< 0.00	All Period RR = 1	28.64134	5	< 0.00	All Period RR = 1	0.146274	5	1.00
		All Cohort RR = 1	25.51954	7	< 0.00	All Cohort RR = 1	26.15634	7	< 0.00	All Cohort RR = 1	0.218291	7	1.00
		All Local Drifts = Net Drift	0.650602	3	0.88	All Local Drifts = Net Drift	0.167187	3	0.98	All Local Drifts = Net Drift	0.112102	3	0.99
	Male	Net Drift = 0	7.967336	1	< 0.00	Net Drift = 0	6.034227	1	0.01	Net Drift = 0	0.071223	1	0.79
		All Age Deviations = 0	5.504188	1	0.02	All Age Deviations = 0	61.92881	1	< 0.00	All Age Deviations = 0	1.437829	1	0.23
		All Period Deviations = 0	0.026705	4	1.00	All Period Deviations = 0	0.078366	4	1.00	All Period Deviations = 0	0.15076	4	1.00
		All Cohort Deviations = 0	0.193209	6	1.00	All Cohort Deviations = 0	0.302194	6	1.00	All Cohort Deviations = 0	0.122337	6	1.00
		All Period RR = 1	8.415774	5	0.13	All Period RR = 1	8.533975	5	0.13	All Period RR = 1	0.262235	5	1.00

		All Cohort RR = 1	8.35064	7	0.30	All Cohort RR = 1	7.176186	7	0.41	All Cohort RR = 1	0.220459	7	1.00
		All Local Drifts = Net Drift	0.099149	3	0.99	All Local Drifts = Net Drift	0.112533	3	0.99	All Local Drifts = Net Drift	0.066426	3	1.00
	Female	Net Drift = 0	14.62036	1	< 0.00	Net Drift = 0	15.21511	1	< 0.00	Net Drift = 0	0.035338	1	0.85
		All Age Deviations = 0	4.997953	1	0.03	All Age Deviations = 0	52.45623	1	< 0.00	All Age Deviations = 0	1.221227	1	0.27
		All Period Deviations = 0	0.048319	4	1.00	All Period Deviations = 0	0.083435	4	1.00	All Period Deviations = 0	0.257413	4	0.99
		All Cohort Deviations = 0	0.43036	6	1.00	All Cohort Deviations = 0	0.286377	6	1.00	All Cohort Deviations = 0	0.135111	6	1.00
		All Period RR = 1	15.29506	5	0.01	All Period RR = 1	19.94177	5	< 0.00	All Period RR = 1	0.30263	5	1.00
		All Cohort RR = 1	15.40727	7	0.03	All Cohort RR = 1	18.87056	7	0.01	All Cohort RR = 1	0.150061	7	1.00
		All Local Drifts = Net Drift	0.393343	3	0.94	All Local Drifts = Net Drift	0.110339	3	0.99	All Local Drifts = Net Drift	0.024567	3	1.00
Eritrea	Both	Net Drift = 0	81.98844	1	< 0.00	Net Drift = 0	46.81994	1	< 0.00	Net Drift = 0	0.039961	1	0.84
		All Age Deviations = 0	37.19897	1	< 0.00	All Age Deviations = 0	249.3284	1	< 0.00	All Age Deviations = 0	17.91557	1	< 0.00
		All Period Deviations = 0	0.560879	4	0.97	All Period Deviations = 0	0.206752	4	1.00	All Period Deviations = 0	0.051443	4	1.00
		All Cohort Deviations = 0	3.079297	6	0.80	All Cohort Deviations = 0	1.915245	6	0.93	All Cohort Deviations = 0	0.047417	6	1.00
		All Period RR = 1	83.31723	5	< 0.00	All Period RR = 1	52.30751	5	< 0.00	All Period RR = 1	0.120848	5	1.00
		All Cohort RR = 1	91.43674	7	< 0.00	All Cohort RR = 1	57.33671	7	< 0.00	All Cohort RR = 1	0.062401	7	1.00
		All Local Drifts = Net Drift	2.356058	3	0.50	All Local Drifts = Net Drift	1.274071	3	0.74	All Local Drifts = Net Drift	0.043426	3	1.00
	Male	Net Drift = 0	43.98163	1	< 0.00	Net Drift = 0	22.84595	1	< 0.00	Net Drift = 0	0.103662	1	0.75
		All Age Deviations = 0	21.09554	1	< 0.00	All Age Deviations = 0	128.6753	1	< 0.00	All Age Deviations = 0	12.94497	1	< 0.00
		All Period Deviations = 0	0.374469	4	0.98	All Period Deviations = 0	0.027282	4	1.00	All Period Deviations = 0	0.022109	4	1.00
		All Cohort Deviations = 0	2.395331	6	0.88	All Cohort Deviations = 0	1.445014	6	0.96	All Cohort Deviations = 0	0.05536	6	1.00
		All Period RR = 1	44.60267	5	< 0.00	All Period RR = 1	25.91158	5	< 0.00	All Period RR = 1	0.126572	5	1.00
		All Cohort RR = 1	50.52388	7	< 0.00	All Cohort RR = 1	30.50721	7	< 0.00	All Cohort RR = 1	0.122836	7	1.00
		All Local Drifts = Net Drift	1.979573	3	0.58	All Local Drifts = Net Drift	1.033648	3	0.79	All Local Drifts = Net Drift	0.05349	3	1.00
	Female	Net Drift = 0	37.80566	1	< 0.00	Net Drift = 0	23.95844	1	< 0.00	Net Drift = 0	0.030662	1	0.86

		All Age Deviations = 0	16.0929	1	< 0.00	All Age Deviations = 0	121.6799	1	< 0.00	All Age Deviations = 0	4.088001	1	0.04
		All Period Deviations = 0	0.242444	4	0.99	All Period Deviations = 0	0.317962	4	0.99	All Period Deviations = 0	0.035488	4	1.00
		All Cohort Deviations = 0	0.989651	6	0.99	All Cohort Deviations = 0	0.996447	6	0.99	All Cohort Deviations = 0	0.133458	6	1.00
		All Period RR = 1	38.63756	5	< 0.00	All Period RR = 1	26.47398	5	< 0.00	All Period RR = 1	0.050079	5	1.00
		All Cohort RR = 1	41.39983	7	< 0.00	All Cohort RR = 1	27.41837	7	< 0.00	All Cohort RR = 1	0.135952	7	1.00
		All Local Drifts = Net Drift	0.627747	3	0.89	All Local Drifts = Net Drift	0.734981	3	0.86	All Local Drifts = Net Drift	0.126984	3	0.99
Eswatini	Both	Net Drift = 0	3.780434	1	0.05	Net Drift = 0	4.223164	1	0.04	Net Drift = 0	0.071918	1	0.79
		All Age Deviations = 0	14.54551	1	< 0.00	All Age Deviations = 0	78.77908	1	< 0.00	All Age Deviations = 0	2.877407	1	0.09
		All Period Deviations = 0	0.014131	4	1.00	All Period Deviations = 0	0.724295	4	0.95	All Period Deviations = 0	0.192867	4	1.00
		All Cohort Deviations = 0	1.554915	6	0.96	All Cohort Deviations = 0	1.81294	6	0.94	All Cohort Deviations = 0	0.084377	6	1.00
		All Period RR = 1	3.815743	5	0.58	All Period RR = 1	5.914479	5	0.31	All Period RR = 1	0.331913	5	1.00
		All Cohort RR = 1	4.984605	7	0.66	All Cohort RR = 1	8.914483	7	0.26	All Cohort RR = 1	1.094616	7	0.99
		All Local Drifts = Net Drift	1.358859	3	0.72	All Local Drifts = Net Drift	1.512778	3	0.68	All Local Drifts = Net Drift	0.018921	3	1.00
	Male	Net Drift = 0	0.696822	1	0.40	Net Drift = 0	2.422714	1	0.12	Net Drift = 0	2.55E-05	1	1.00
		All Age Deviations = 0	7.081665	1	0.01	All Age Deviations = 0	34.53865	1	< 0.00	All Age Deviations = 0	0.857582	1	0.35
		All Period Deviations = 0	0.020582	4	1.00	All Period Deviations = 0	0.273512	4	0.99	All Period Deviations = 0	0.001643	4	1.00
		All Cohort Deviations = 0	1.35838	6	0.97	All Cohort Deviations = 0	1.959886	6	0.92	All Cohort Deviations = 0	0.162989	6	1.00
		All Period RR = 1	0.718711	5	0.98	All Period RR = 1	3.208729	5	0.67	All Period RR = 1	0.001677	5	1.00
		All Cohort RR = 1	1.906829	7	0.96	All Cohort RR = 1	6.326651	7	0.50	All Cohort RR = 1	0.163496	7	1.00
		All Local Drifts = Net Drift	1.146851	3	0.77	All Local Drifts = Net Drift	1.725307	3	0.63	All Local Drifts = Net Drift	0.052023	3	1.00
	Female	Net Drift = 0	3.644613	1	0.06	Net Drift = 0	1.788696	1	0.18	Net Drift = 0	0.044415	1	0.83
		All Age Deviations = 0	7.476811	1	0.01	All Age Deviations = 0	46.7884	1	< 0.00	All Age Deviations = 0	2.96883	1	0.08
		All Period Deviations = 0	0.055036	4	1.00	All Period Deviations = 0	0.420189	4	0.98	All Period Deviations = 0	0.275689	4	0.99
		All Cohort Deviations = 0	0.380865	6	1.00	All Cohort Deviations = 0	0.340447	6	1.00	All Cohort Deviations = 0	0.061963	6	1.00

		All Period RR = 1	3.691042	5	0.59	All Period RR = 1	2.627201	5	0.76	All Period RR = 1	0.343231	5	1.00
		All Cohort RR = 1	3.924842	7	0.79	All Cohort RR = 1	2.993249	7	0.89	All Cohort RR = 1	0.51016	7	1.00
		All Local Drifts = Net Drift	0.327138	3	0.95	All Local Drifts = Net Drift	0.165407	3	0.98	All Local Drifts = Net Drift	0.021813	3	1.00
Ethiopia	Both	Net Drift = 0	1917.326	1	< 0.00	Net Drift = 0	318.4595	1	< 0.00	Net Drift = 0	34.46346	1	< 0.00
		All Age Deviations = 0	385.1167	1	< 0.00	All Age Deviations = 0	1235.336	1	< 0.00	All Age Deviations = 0	450.6323	1	< 0.00
		All Period Deviations = 0	8.759602	4	< 0.07	All Period Deviations = 0	15.57555	4	< 0.00	All Period Deviations = 0	31.94424	4	< 0.00
		All Cohort Deviations = 0	104.4545	6	< 0.00	All Cohort Deviations = 0	30.49148	6	< 0.00	All Cohort Deviations = 0	4.02087	6	0.67
		All Period RR = 1	1973.656	5	< 0.00	All Period RR = 1	432.5255	5	< 0.00	All Period RR = 1	71.7089	5	< 0.00
		All Cohort RR = 1	2208.409	7	< 0.00	All Cohort RR = 1	375.701	7	< 0.00	All Cohort RR = 1	224.623	7	< 0.00
		All Local Drifts = Net Drift	89.14739	3	< 0.00	All Local Drifts = Net Drift	28.29843	3	< 0.00	All Local Drifts = Net Drift	3.343746	3	0.34
	Male	Net Drift = 0	1007.089	1	< 0.00	Net Drift = 0	432.0334	1	< 0.00	Net Drift = 0	50.25074	1	< 0.00
		All Age Deviations = 0	284.8325	1	< 0.00	All Age Deviations = 0	2173.325	1	< 0.00	All Age Deviations = 0	338.0639	1	< 0.00
		All Period Deviations = 0	11.73542	4	0.02	All Period Deviations = 0	10.24054	4	0.04	All Period Deviations = 0	40.6403	4	< 0.00
		All Cohort Deviations = 0	47.0057	6	< 0.00	All Cohort Deviations = 0	36.63295	6	< 0.00	All Cohort Deviations = 0	1.445524	6	0.96
		All Period RR = 1	1020.574	5	< 0.00	All Period RR = 1	549.2212	5	< 0.00	All Period RR = 1	93.31684	5	< 0.00
		All Cohort RR = 1	1147.476	7	< 0.00	All Cohort RR = 1	510.9091	7	< 0.00	All Cohort RR = 1	291.2888	7	< 0.00
		All Local Drifts = Net Drift	42.65961	3	< 0.00	All Local Drifts = Net Drift	34.99496	3	< 0.00	All Local Drifts = Net Drift	1.200379	3	0.75
	Female	Net Drift = 0	1771.044	1	< 0.00	Net Drift = 0	231.1152	1	< 0.00	Net Drift = 0	2.945774	1	0.09
		All Age Deviations = 0	254.5496	1	< 0.00	All Age Deviations = 0	699.3554	1	< 0.00	All Age Deviations = 0	114.7815	1	< 0.00
		All Period Deviations = 0	3.889933	4	0.42	All Period Deviations = 0	18.80581	4	< 0.00	All Period Deviations = 0	0.292131	4	0.99
		All Cohort Deviations = 0	117.005	6	< 0.00	All Cohort Deviations = 0	27.26416	6	< 0.00	All Cohort Deviations = 0	0.215711	6	1.00
		All Period RR = 1	1857.348	5	< 0.00	All Period RR = 1	335.114	5	< 0.00	All Period RR = 1	4.430231	5	0.49
		All Cohort RR = 1	2067.952	7	< 0.00	All Cohort RR = 1	273.6625	7	< 0.00	All Cohort RR = 1	8.031052	7	0.33
		All Local Drifts = Net Drift	93.85732	3	< 0.00	All Local Drifts = Net Drift	24.19812	3	< 0.00	All Local Drifts = Net Drift	0.209447	3	0.98

Gabon	Both	Net Drift = 0	3.170897	1	0.07	Net Drift = 0	0.415742	1	0.52	Net Drift = 0	0.027922	1	0.87
		All Age Deviations = 0	15.78659	1	< 0.00	All Age Deviations = 0	182.8718	1	< 0.00	All Age Deviations = 0	3.984582	1	0.05
		All Period Deviations = 0	0.027321	4	1.00	All Period Deviations = 0	0.303979	4	0.99	All Period Deviations = 0	0.105156	4	1.00
		All Cohort Deviations = 0	0.057757	6	1.00	All Cohort Deviations = 0	0.515824	6	1.00	All Cohort Deviations = 0	0.072595	6	1.00
		All Period RR = 1	3.234451	5	0.66	All Period RR = 1	0.584053	5	0.99	All Period RR = 1	0.11886	5	1.00
		All Cohort RR = 1	3.298482	7	0.86	All Cohort RR = 1	0.640293	7	1.00	All Cohort RR = 1	0.116707	7	1.00
		All Local Drifts = Net Drift	0.055931	3	1.00	All Local Drifts = Net Drift	0.505839	3	0.92	All Local Drifts = Net Drift	0.031266	3	1.00
	Male	Net Drift = 0	0.937256	1	0.33	Net Drift = 0	0.185171	1	0.67	Net Drift = 0	0.000522	1	0.98
		All Age Deviations = 0	7.127048	1	0.01	All Age Deviations = 0	92.00042	1	< 0.00	All Age Deviations = 0	2.084475	1	0.15
		All Period Deviations = 0	0.021807	4	1.00	All Period Deviations = 0	0.009918	4	1.00	All Period Deviations = 0	0.145945	4	1.00
		All Cohort Deviations = 0	0.023106	6	1.00	All Cohort Deviations = 0	0.288768	6	1.00	All Cohort Deviations = 0	0.106532	6	1.00
		All Period RR = 1	0.960745	5	0.97	All Period RR = 1	0.196758	5	1.00	All Period RR = 1	0.155039	5	1.00
		All Cohort RR = 1	0.98366	7	1.00	All Cohort RR = 1	0.335479	7	1.00	All Cohort RR = 1	0.112076	7	1.00
		All Local Drifts = Net Drift	0.014219	3	1.00	All Local Drifts = Net Drift	0.253822	3	0.97	All Local Drifts = Net Drift	0.041253	3	1.00
	Female	Net Drift = 0	2.917622	1	0.09	Net Drift = 0	0.419865	1	0.52	Net Drift = 0	0.00585	1	0.94
		All Age Deviations = 0	8.801426	1	< 0.00	All Age Deviations = 0	92.62072	1	< 0.00	All Age Deviations = 0	2.371331	1	0.12
		All Period Deviations = 0	0.021603	4	1.00	All Period Deviations = 0	0.524109	4	0.97	All Period Deviations = 0	0.133488	4	1.00
		All Cohort Deviations = 0	0.092913	6	1.00	All Cohort Deviations = 0	0.286809	6	1.00	All Cohort Deviations = 0	0.087017	6	1.00
		All Period RR = 1	3.001398	5	0.70	All Period RR = 1	0.779549	5	0.98	All Period RR = 1	0.151196	5	1.00
		All Cohort RR = 1	3.087057	7	0.88	All Cohort RR = 1	0.504531	7	1.00	All Cohort RR = 1	0.104371	7	1.00
		All Local Drifts = Net Drift	0.092656	3	0.99	All Local Drifts = Net Drift	0.283438	3	0.96	All Local Drifts = Net Drift	0.021783	3	1.00
Gambia	Both	Net Drift = 0	18.82923	1	< 0.00	Net Drift = 0	20.79149	1	< 0.00	Net Drift = 0	0.680406	1	0.41
		All Age Deviations = 0	17.37496	1	< 0.00	All Age Deviations = 0	119.6228	1	< 0.00	All Age Deviations = 0	3.79049	1	0.05
		All Period Deviations = 0	0.073568	4	1.00	All Period Deviations = 0	0.072708	4	1.00	All Period Deviations = 0	0.147343	4	1.00

		All Cohort Deviations = 0	0.352473	6	1.00	All Cohort Deviations = 0	0.077183	6	1.00	All Cohort Deviations = 0	1.19945	6	0.98
		All Period RR = 1	19.44349	5	< 0.00	All Period RR = 1	23.63184	5	< 0.00	All Period RR = 1	0.980477	5	0.96
		All Cohort RR = 1	20.11414	7	0.01	All Cohort RR = 1	22.90084	7	< 0.00	All Cohort RR = 1	1.230608	7	0.99
		All Local Drifts = Net Drift	0.325861	3	0.96	All Local Drifts = Net Drift	0.032355	3	1.00	All Local Drifts = Net Drift	1.142152	3	0.77
	Male	Net Drift = 0	4.081772	1	0.04	Net Drift = 0	3.60613	1	0.06	Net Drift = 0	0.000166	1	0.99
		All Age Deviations = 0	8.617522	1	< 0.00	All Age Deviations = 0	30.2453	1	< 0.00	All Age Deviations = 0	1.550639	1	0.21
		All Period Deviations = 0	0.039609	4	1.00	All Period Deviations = 0	0.124125	4	1.00	All Period Deviations = 0	0.322872	4	0.99
		All Cohort Deviations = 0	0.089358	6	1.00	All Cohort Deviations = 0	0.062655	6	1.00	All Cohort Deviations = 0	0.1478	6	1.00
		All Period RR = 1	4.306895	5	0.51	All Period RR = 1	4.584745	5	0.47	All Period RR = 1	0.325974	5	1.00
		All Cohort RR = 1	4.303252	7	0.74	All Cohort RR = 1	4.129067	7	0.76	All Cohort RR = 1	0.148096	7	1.00
		All Local Drifts = Net Drift	0.044201	3	1.00	All Local Drifts = Net Drift	0.024744	3	1.00	All Local Drifts = Net Drift	0.136362	3	0.99
	Female	Net Drift = 0	18.02785	1	< 0.00	Net Drift = 0	17.77924	1	< 0.00	Net Drift = 0	0.029215	1	0.86
		All Age Deviations = 0	9.580923	1	< 0.00	All Age Deviations = 0	88.84231	1	< 0.00	All Age Deviations = 0	2.338882	1	0.13
		All Period Deviations = 0	0.011739	4	1.00	All Period Deviations = 0	0.138438	4	1.00	All Period Deviations = 0	0.158753	4	1.00
		All Cohort Deviations = 0	0.424536	6	1.00	All Cohort Deviations = 0	0.168151	6	1.00	All Cohort Deviations = 0	0.38571	6	1.00
		All Period RR = 1	18.33879	5	< 0.00	All Period RR = 1	19.56148	5	< 0.00	All Period RR = 1	0.173972	5	1.00
		All Cohort RR = 1	19.84938	7	0.01	All Cohort RR = 1	19.32466	7	0.01	All Cohort RR = 1	0.408157	7	1.00
		All Local Drifts = Net Drift	0.388632	3	0.94	All Local Drifts = Net Drift	0.152887	3	0.98	All Local Drifts = Net Drift	0.302082	3	0.96
Ghana	Both	Net Drift = 0	585.5822	1	< 0.00	Net Drift = 0	399.8757	1	< 0.00	Net Drift = 0	0.027625	1	0.87
		All Age Deviations = 0	399.3898	1	< 0.00	All Age Deviations = 0	1601.776	1	< 0.00	All Age Deviations = 0	39.55015	1	< 0.00
		All Period Deviations = 0	1.634831	4	0.80	All Period Deviations = 0	27.96444	4	< 0.00	All Period Deviations = 0	0.096421	4	1.00
		All Cohort Deviations = 0	12.98442	6	0.04	All Cohort Deviations = 0	14.74084	6	< 0.02	All Cohort Deviations = 0	0.074136	6	1.00
		All Period RR = 1	601.7483	5	< 0.00	All Period RR = 1	550.4351	5	< 0.00	All Period RR = 1	0.205478	5	1.00
		All Cohort RR = 1	640.5734	7	< 0.00	All Cohort RR = 1	490.3956	7	< 0.00	All Cohort RR = 1	0.478032	7	1.00

		All Local Drifts = Net Drift	10.13091	3	0.02	All Local Drifts = Net Drift	12.24022	3	0.01	All Local Drifts = Net Drift	0.0729	3	0.99
	Male	Net Drift = 0	208.4875	1	< 0.00	Net Drift = 0	145.4076	1	< 0.00	Net Drift = 0	0.046993	1	0.83
		All Age Deviations = 0	161.7009	1	< 0.00	All Age Deviations = 0	600.3645	1	< 0.00	All Age Deviations = 0	18.93402	1	< 0.00
		All Period Deviations = 0	0.722625	4	0.95	All Period Deviations = 0	31.56026	4	< 0.00	All Period Deviations = 0	0.021205	4	1.00
		All Cohort Deviations = 0	5.829025	6	0.44	All Cohort Deviations = 0	9.687788	6	0.14	All Cohort Deviations = 0	0.01703	6	1.00
		All Period RR = 1	214.7009	5	< 0.00	All Period RR = 1	238.2686	5	< 0.00	All Period RR = 1	0.068663	5	1.00
		All Cohort RR = 1	228.1991	7	< 0.00	All Cohort RR = 1	185.4214	7	< 0.00	All Cohort RR = 1	0.100883	7	1.00
		All Local Drifts = Net Drift	4.206001	3	0.24	All Local Drifts = Net Drift	8.01365	3	0.05	All Local Drifts = Net Drift	0.013838	3	1.00
	Female	Net Drift = 0	391.5727	1	< 0.00	Net Drift = 0	257.3412	1	< 0.00	Net Drift = 0	0.022673	1	0.88
		All Age Deviations = 0	241.9882	1	< 0.00	All Age Deviations = 0	1019.066	1	< 0.00	All Age Deviations = 0	20.54516	1	0.00
		All Period Deviations = 0	1.379417	4	0.85	All Period Deviations = 0	5.071483	4	0.28	All Period Deviations = 0	0.11637	4	1.00
		All Cohort Deviations = 0	8.175866	6	0.23	All Cohort Deviations = 0	5.636122	6	0.47	All Cohort Deviations = 0	0.154188	6	1.00
		All Period RR = 1	402.4427	5	< 0.00	All Period RR = 1	322.4021	5	< 0.00	All Period RR = 1	0.189385	5	1.00
		All Cohort RR = 1	430.8527	7	< 0.00	All Cohort RR = 1	308.4918	7	< 0.00	All Cohort RR = 1	0.539518	7	1.00
		All Local Drifts = Net Drift	6.883146	3	0.08	All Local Drifts = Net Drift	4.560081	3	0.21	All Local Drifts = Net Drift	0.108755	3	0.99
Guinea	Both	Net Drift = 0	343.7701	1	< 0.00	Net Drift = 0	273.2069	1	< 0.00	Net Drift = 0	0.051092	1	0.82
		All Age Deviations = 0	67.30913	1	< 0.00	All Age Deviations = 0	370.5466	1	< 0.00	All Age Deviations = 0	16.1677	1	< 0.00
		All Period Deviations = 0	0.167495	4	1.00	All Period Deviations = 0	0.18406	4	1.00	All Period Deviations = 0	0.043297	4	1.00
		All Cohort Deviations = 0	11.3612	6	0.08	All Cohort Deviations = 0	4.064415	6	0.67	All Cohort Deviations = 0	0.113807	6	1.00
		All Period RR = 1	348.6347	5	< 0.00	All Period RR = 1	307.4916	5	< 0.00	All Period RR = 1	0.115779	5	1.00
		All Cohort RR = 1	405.6808	7	< 0.00	All Cohort RR = 1	303.7224	7	< 0.00	All Cohort RR = 1	0.114574	7	1.00
		All Local Drifts = Net Drift	10.92248	3	< 0.01	All Local Drifts = Net Drift	3.856458	3	0.28	All Local Drifts = Net Drift	0.083337	3	0.99
	Male	Net Drift = 0	128.3245	1	< 0.00	Net Drift = 0	100.7296	1	< 0.00	Net Drift = 0	0.366195	1	0.55
		All Age Deviations = 0	37.69879	1	< 0.00	All Age Deviations = 0	148.4777	1	< 0.00	All Age Deviations = 0	8.384355	1	< 0.00

		All Period Deviations = 0	0.097617	4	1.00	All Period Deviations = 0	0.106117	4	1.00	All Period Deviations = 0	0.044002	4	1.00
		All Cohort Deviations = 0	3.49829	6	0.74	All Cohort Deviations = 0	1.631442	6	0.95	All Cohort Deviations = 0	0.15066	6	1.00
		All Period RR = 1	130.1302	5	< 0.00	All Period RR = 1	114.5246	5	< 0.00	All Period RR = 1	0.367618	5	1.00
		All Cohort RR = 1	145.8108	7	< 0.00	All Cohort RR = 1	114.2974	7	< 0.00	All Cohort RR = 1	0.422007	7	1.00
		All Local Drifts = Net Drift	3.472348	3	0.32	All Local Drifts = Net Drift	1.344326	3	0.72	All Local Drifts = Net Drift	0.146845	3	0.99
	Female	Net Drift = 0	234.8285	1	< 0.00	Net Drift = 0	175.9918	1	< 0.00	Net Drift = 0	0.016093	1	0.90
		All Age Deviations = 0	29.10211	1	< 0.00	All Age Deviations = 0	222.3315	1	< 0.00	All Age Deviations = 0	8.615613	1	< 0.00
		All Period Deviations = 0	0.194344	4	1.00	All Period Deviations = 0	0.765105	4	0.94	All Period Deviations = 0	0.166378	4	1.00
		All Cohort Deviations = 0	9.927651	6	0.13	All Cohort Deviations = 0	4.217263	6	0.65	All Cohort Deviations = 0	0.206437	6	1.00
		All Period RR = 1	237.9439	5	< 0.00	All Period RR = 1	197.0605	5	< 0.00	All Period RR = 1	0.170114	5	1.00
		All Cohort RR = 1	289.0395	7	< 0.00	All Cohort RR = 1	193.195	7	< 0.00	All Cohort RR = 1	0.287534	7	1.00
		All Local Drifts = Net Drift	9.26181	3	0.03	All Local Drifts = Net Drift	4.120328	3	0.25	All Local Drifts = Net Drift	0.048858	3	1.00
Guinea-Bissau	Both	Net Drift = 0	33.33087	1	< 0.00	Net Drift = 0	27.91019	1	< 0.00	Net Drift = 0	0.655448	1	0.42
		All Age Deviations = 0	13.75931	1	< 0.00	All Age Deviations = 0	68.95142	1	< 0.00	All Age Deviations = 0	3.196742	1	0.07
		All Period Deviations = 0	0.062928	4	1.00	All Period Deviations = 0	0.040555	4	1.00	All Period Deviations = 0	0.079878	4	1.00
		All Cohort Deviations = 0	0.286602	6	1.00	All Cohort Deviations = 0	0.039226	6	1.00	All Cohort Deviations = 0	1.34684	6	0.97
		All Period RR = 1	34.08244	5	< 0.00	All Period RR = 1	31.70055	5	< 0.00	All Period RR = 1	1.049595	5	0.96
		All Cohort RR = 1	37.1783	7	< 0.00	All Cohort RR = 1	30.93753	7	< 0.00	All Cohort RR = 1	1.37712	7	0.99
		All Local Drifts = Net Drift	0.247123	3	0.97	All Local Drifts = Net Drift	0.035956	3	1.00	All Local Drifts = Net Drift	1.266493	3	0.74
	Male	Net Drift = 0	8.49078	1	< 0.00	Net Drift = 0	6.332588	1	0.01	Net Drift = 0	0.036746	1	0.85
		All Age Deviations = 0	7.175929	1	0.01	All Age Deviations = 0	27.14986	1	< 0.00	All Age Deviations = 0	1.851975	1	0.17
		All Period Deviations = 0	0.046817	4	1.00	All Period Deviations = 0	0.054903	4	1.00	All Period Deviations = 0	0.115998	4	1.00
		All Cohort Deviations = 0	0.062683	6	1.00	All Cohort Deviations = 0	0.027781	6	1.00	All Cohort Deviations = 0	0.277351	6	1.00
		All Period RR = 1	8.7766	5	0.12	All Period RR = 1	7.390713	5	0.19	All Period RR = 1	0.159581	5	1.00

		All Cohort RR = 1	9.056224	7	0.25	All Cohort RR = 1	7.0693 66	7	0.42	All Cohort RR = 1	0.3222 38	7	1.00
		All Local Drifts = Net Drift	0.057752	3	1.00	All Local Drifts = Net Drift	0.0147 38	3	1.00	All Local Drifts = Net Drift	0.0283 28	3	1.00
	Female	Net Drift = 0	29.7325	1	< 0.00	Net Drift = 0	24.924 47	1	< 0.00	Net Drift = 0	0.0271 02	1	0.87
		All Age Deviations = 0	7.579764	1	0.01	All Age Deviations = 0	42.147 56	1	< 0.00	All Age Deviations = 0	1.7466 17	1	0.19
		All Period Deviations = 0	0.05164	4	1.00	All Period Deviations = 0	0.0095 47	4	1.00	All Period Deviations = 0	0.2811 19	4	0.99
		All Cohort Deviations = 0	0.661247	6	1.00	All Cohort Deviations = 0	0.0876 67	6	1.00	All Cohort Deviations = 0	0.4343 34	6	1.00
		All Period RR = 1	30.33684	5	< 0.00	All Period RR = 1	28.115 81	5	< 0.00	All Period RR = 1	0.2811 79	5	1.00
		All Cohort RR = 1	34.97132	7	< 0.00	All Cohort RR = 1	27.467 61	7	< 0.00	All Cohort RR = 1	0.4487 88	7	1.00
		All Local Drifts = Net Drift	0.641643	3	0.89	All Local Drifts = Net Drift	0.0862 9	3	0.99	All Local Drifts = Net Drift	0.3154 36	3	0.96
Kenya	Both	Net Drift = 0	293.126	1	< 0.00	Net Drift = 0	97.401 15	1	< 0.00	Net Drift = 0	0.1383 99	1	0.71
		All Age Deviations = 0	507.9079	1	< 0.00	All Age Deviations = 0	3032.1 98	1	< 0.00	All Age Deviations = 0	115.98 05	1	< 0.00
		All Period Deviations = 0	1.243707	4	0.87	All Period Deviations = 0	13.659 42	4	0.01	All Period Deviations = 0	10.566 24	4	0.03
		All Cohort Deviations = 0	22.43192	6	< 0.00	All Cohort Deviations = 0	7.1383 36	6	0.31	All Cohort Deviations = 0	15.446 61	6	0.02
		All Period RR = 1	300.3384	5	< 0.00	All Period RR = 1	107.04 47	5	< 0.00	All Period RR = 1	11.932 24	5	0.04
		All Cohort RR = 1	334.3299	7	< 0.00	All Cohort RR = 1	113.47 34	7	< 0.00	All Cohort RR = 1	24.067 65	7	< 0.00
		All Local Drifts = Net Drift	14.16869	3	< 0.00	All Local Drifts = Net Drift	1.5317 4	3	0.67	All Local Drifts = Net Drift	14.875 56	3	< 0.00
	Male	Net Drift = 0	122.9089	1	< 0.00	Net Drift = 0	29.474 32	1	< 0.00	Net Drift = 0	0.1318 66	1	0.72
		All Age Deviations = 0	242.457	1	< 0.00	All Age Deviations = 0	926.63 74	1	< 0.00	All Age Deviations = 0	56.721 53	1	< 0.00
		All Period Deviations = 0	1.160989	4	0.88	All Period Deviations = 0	2.9117 94	4	0.57	All Period Deviations = 0	18.999 6	4	< 0.00
		All Cohort Deviations = 0	13.73188	6	0.03	All Cohort Deviations = 0	3.4036 16	6	0.76	All Cohort Deviations = 0	29.437 22	6	< 0.00
		All Period RR = 1	124.6609	5	< 0.00	All Period RR = 1	30.730 59	5	< 0.00	All Period RR = 1	22.257 34	5	< 0.00
		All Cohort RR = 1	145.5813	7	< 0.00	All Cohort RR = 1	34.981 51	7	< 0.00	All Cohort RR = 1	54.871 34	7	< 0.00
		All Local Drifts = Net Drift	8.465579	3	0.04	All Local Drifts = Net Drift	1.3140 62	3	0.73	All Local Drifts = Net Drift	28.112 46	3	0.00
	Female	Net Drift = 0	171.4842	1	< 0.00	Net Drift = 0	54.689 07	1	< 0.00	Net Drift = 0	0.1657 22	1	0.68

		All Age Deviations = 0	259.6482	1	< 0.00	All Age Deviations = 0	1661.872	1	< 0.00	All Age Deviations = 0	59.57333	1	0.00
		All Period Deviations = 0	1.282695	4	0.86	All Period Deviations = 0	11.62057	4	0.02	All Period Deviations = 0	0.152109	4	1.00
		All Cohort Deviations = 0	8.943009	6	0.18	All Cohort Deviations = 0	3.343869	6	0.76	All Cohort Deviations = 0	0.075824	6	1.00
		All Period RR = 1	178.782	5	< 0.00	All Period RR = 1	64.70903	5	< 0.00	All Period RR = 1	0.264362	5	1.00
		All Cohort RR = 1	190.2067	7	< 0.00	All Cohort RR = 1	63.65979	7	< 0.00	All Cohort RR = 1	1.104992	7	0.99
		All Local Drifts = Net Drift	5.767013	3	0.12	All Local Drifts = Net Drift	0.78317	3	0.85	All Local Drifts = Net Drift	0.071763	3	0.99
Lesotho	Both	Net Drift = 0	1.913899	1	0.17	Net Drift = 0	7.236551	1	0.01	Net Drift = 0	0.078647	1	0.78
		All Age Deviations = 0	21.53737	1	< 0.00	All Age Deviations = 0	181.4582	1	< 0.00	All Age Deviations = 0	4.027327	1	0.04
		All Period Deviations = 0	0.024573	4	1.00	All Period Deviations = 0	0.250679	4	0.99	All Period Deviations = 0	0.26714	4	0.99
		All Cohort Deviations = 0	0.116098	6	1.00	All Cohort Deviations = 0	1.168298	6	0.98	All Cohort Deviations = 0	0.416264	6	1.00
		All Period RR = 1	1.964545	5	0.85	All Period RR = 1	8.379419	5	0.14	All Period RR = 1	0.442599	5	0.99
		All Cohort RR = 1	1.961908	7	0.96	All Cohort RR = 1	9.560562	7	0.21	All Cohort RR = 1	0.534898	7	1.00
		All Local Drifts = Net Drift	0.101579	3	0.99	All Local Drifts = Net Drift	1.083758	3	0.78	All Local Drifts = Net Drift	0.365894	3	0.95
	Male	Net Drift = 0	3.003094	1	0.08	Net Drift = 0	11.80914	1	< 0.00	Net Drift = 0	0.000567	1	0.98
		All Age Deviations = 0	9.277733	1	< 0.00	All Age Deviations = 0	73.51394	1	< 0.00	All Age Deviations = 0	3.787625	1	0.05
		All Period Deviations = 0	0.038763	4	1.00	All Period Deviations = 0	0.097528	4	1.00	All Period Deviations = 0	0.038191	4	1.00
		All Cohort Deviations = 0	0.133651	6	1.00	All Cohort Deviations = 0	1.115193	6	0.98	All Cohort Deviations = 0	0.071861	6	1.00
		All Period RR = 1	3.060906	5	0.69	All Period RR = 1	13.21295	5	0.02	All Period RR = 1	0.038287	5	1.00
		All Cohort RR = 1	3.107664	7	0.87	All Cohort RR = 1	16.55813	7	0.02	All Cohort RR = 1	0.08892	7	1.00
		All Local Drifts = Net Drift	0.112968	3	0.99	All Local Drifts = Net Drift	0.977912	3	0.81	All Local Drifts = Net Drift	0.022047	3	1.00
	Female	Net Drift = 0	0.00802	1	0.93	Net Drift = 0	0.473466	1	0.49	Net Drift = 0	0.016522	1	0.90
		All Age Deviations = 0	12.69522	1	< 0.00	All Age Deviations = 0	109.6004	1	< 0.00	All Age Deviations = 0	3.056088	1	0.08
		All Period Deviations = 0	0.041952	4	1.00	All Period Deviations = 0	0.079428	4	1.00	All Period Deviations = 0	0.059356	4	1.00
		All Cohort Deviations = 0	0.228131	6	1.00	All Cohort Deviations = 0	0.393098	6	1.00	All Cohort Deviations = 0	0.059763	6	1.00

		All Period RR = 1	0.051321	5	1.00	All Period RR = 1	0.627949	5	0.99	All Period RR = 1	0.068047	5	1.00
		All Cohort RR = 1	0.233044	7	1.00	All Cohort RR = 1	0.705949	7	1.00	All Cohort RR = 1	0.163318	7	1.00
		All Local Drifts = Net Drift	0.191412	3	0.98	All Local Drifts = Net Drift	0.377353	3	0.94	All Local Drifts = Net Drift	0.017744	3	1.00
Liberia	Both	Net Drift = 0	40.36098	1	< 0.00	Net Drift = 0	57.51481	1	< 0.00	Net Drift = 0	0.254951	1	0.61
		All Age Deviations = 0	68.39911	1	< 0.00	All Age Deviations = 0	298.3194	1	< 0.00	All Age Deviations = 0	5.000989	1	0.03
		All Period Deviations = 0	0.263023	4	0.99	All Period Deviations = 0	23.599	4	< 0.00	All Period Deviations = 0	0.124606	4	1.00
		All Cohort Deviations = 0	1.118825	6	0.98	All Cohort Deviations = 0	0.996701	6	0.99	All Cohort Deviations = 0	0.499184	6	1.00
		All Period RR = 1	42.53597	5	< 0.00	All Period RR = 1	67.35919	5	< 0.00	All Period RR = 1	0.288259	5	1.00
		All Cohort RR = 1	43.6732	7	< 0.00	All Cohort RR = 1	61.81926	7	< 0.00	All Cohort RR = 1	0.499225	7	1.00
		All Local Drifts = Net Drift	0.572782	3	0.90	All Local Drifts = Net Drift	0.56111	3	0.91	All Local Drifts = Net Drift	0.496754	3	0.92
	Male	Net Drift = 0	11.39383	1	< 0.00	Net Drift = 0	4.984468	1	0.03	Net Drift = 0	0.493358	1	0.48
		All Age Deviations = 0	32.94592	1	< 0.00	All Age Deviations = 0	69.72076	1	< 0.00	All Age Deviations = 0	3.225036	1	0.07
		All Period Deviations = 0	0.184405	4	1.00	All Period Deviations = 0	0.478492	4	0.98	All Period Deviations = 0	0.039385	4	1.00
		All Cohort Deviations = 0	0.554821	6	1.00	All Cohort Deviations = 0	0.182891	6	1.00	All Cohort Deviations = 0	1.101829	6	0.98
		All Period RR = 1	12.33469	5	0.03	All Period RR = 1	7.216527	5	0.21	All Period RR = 1	0.716274	5	0.98
		All Cohort RR = 1	12.32297	7	0.09	All Cohort RR = 1	5.330288	7	0.62	All Cohort RR = 1	1.132201	7	0.99
		All Local Drifts = Net Drift	0.272286	3	0.97	All Local Drifts = Net Drift	0.099632	3	0.99	All Local Drifts = Net Drift	1.002795	3	0.80
	Female	Net Drift = 0	30.2131	1	< 0.00	Net Drift = 0	66.82494	1	< 0.00	Net Drift = 0	0.001288	1	0.97
		All Age Deviations = 0	36.23068	1	< 0.00	All Age Deviations = 0	231.8349	1	< 0.00	All Age Deviations = 0	1.34853	1	0.25
		All Period Deviations = 0	0.10265	4	1.00	All Period Deviations = 0	40.40347	4	< 0.00	All Period Deviations = 0	0.027946	4	1.00
		All Cohort Deviations = 0	0.417214	6	1.00	All Cohort Deviations = 0	0.751409	6	0.99	All Cohort Deviations = 0	0.162169	6	1.00
		All Period RR = 1	31.3276	5	< 0.00	All Period RR = 1	86.42004	5	< 0.00	All Period RR = 1	0.028175	5	1.00
		All Cohort RR = 1	33.46957	7	< 0.00	All Cohort RR = 1	71.91444	7	< 0.00	All Cohort RR = 1	0.227385	7	1.00
		All Local Drifts = Net Drift	0.19014	3	0.98	All Local Drifts = Net Drift	0.415385	3	0.94	All Local Drifts = Net Drift	0.050404	3	1.00

Libya	Both	Net Drift = 0	25.89736	1	< 0.00	Net Drift = 0	24.2958	1	< 0.00	Net Drift = 0	0.001294	1	0.97
		All Age Deviations = 0	102.5681	1	< 0.00	All Age Deviations = 0	365.2921	1	< 0.00	All Age Deviations = 0	5.074354	1	0.02
		All Period Deviations = 0	0.157993	4	1.00	All Period Deviations = 0	0.046544	4	1.00	All Period Deviations = 0	0.036957	4	1.00
		All Cohort Deviations = 0	2.206386	6	0.90	All Cohort Deviations = 0	0.919989	6	0.99	All Cohort Deviations = 0	0.067184	6	1.00
		All Period RR = 1	26.32581	5	< 0.00	All Period RR = 1	25.28455	5	< 0.00	All Period RR = 1	0.03724	5	1.00
		All Cohort RR = 1	30.67942	7	< 0.00	All Cohort RR = 1	31.068	7	< 0.00	All Cohort RR = 1	0.068199	7	1.00
		All Local Drifts = Net Drift	1.766747	3	0.62	All Local Drifts = Net Drift	0.358763	3	0.95	All Local Drifts = Net Drift	0.024486	3	1.00
	Male	Net Drift = 0	3.157295	1	0.08	Net Drift = 0	6.867312	1	0.01	Net Drift = 0	0.00074	1	0.98
		All Age Deviations = 0	60.759	1	< 0.00	All Age Deviations = 0	195.7922	1	< 0.00	All Age Deviations = 0	2.500354	1	0.11
		All Period Deviations = 0	0.246106	4	0.99	All Period Deviations = 0	0.134291	4	1.00	All Period Deviations = 0	0.018019	4	1.00
		All Cohort Deviations = 0	0.221109	6	1.00	All Cohort Deviations = 0	0.56756	6	1.00	All Cohort Deviations = 0	0.068037	6	1.00
		All Period RR = 1	3.478277	5	0.63	All Period RR = 1	7.373161	5	0.19	All Period RR = 1	0.018529	5	1.00
		All Cohort RR = 1	3.69576	7	0.81	All Cohort RR = 1	8.80056	7	0.27	All Cohort RR = 1	0.0732	7	1.00
		All Local Drifts = Net Drift	0.160607	3	0.98	All Local Drifts = Net Drift	0.369847	3	0.95	All Local Drifts = Net Drift	0.011327	3	1.00
	Female	Net Drift = 0	29.56559	1	< 0.00	Net Drift = 0	18.29594	1	< 0.00	Net Drift = 0	2.69E-08	1	1.00
		All Age Deviations = 0	42.69186	1	< 0.00	All Age Deviations = 0	173.9241	1	< 0.00	All Age Deviations = 0	3.163612	1	0.08
		All Period Deviations = 0	0.088549	4	1.00	All Period Deviations = 0	0.166184	4	1.00	All Period Deviations = 0	0.039528	4	1.00
		All Cohort Deviations = 0	3.67682	6	0.72	All Cohort Deviations = 0	1.702938	6	0.94	All Cohort Deviations = 0	0.018265	6	1.00
		All Period RR = 1	29.8307	5	< 0.00	All Period RR = 1	19.70852	5	< 0.00	All Period RR = 1	0.039593	5	1.00
		All Cohort RR = 1	36.95251	7	< 0.00	All Cohort RR = 1	25.82652	7	< 0.00	All Cohort RR = 1	0.022328	7	1.00
		All Local Drifts = Net Drift	3.139444	3	0.37	All Local Drifts = Net Drift	1.289297	3	0.73	All Local Drifts = Net Drift	0.001394	3	1.00
Madagascar	Both	Net Drift = 0	340.2337	1	< 0.00	Net Drift = 0	190.0507	1	< 0.00	Net Drift = 0	0.225736	1	0.63
		All Age Deviations = 0	165.2005	1	< 0.00	All Age Deviations = 0	1155.264	1	< 0.00	All Age Deviations = 0	69.98905	1	< 0.00
		All Period Deviations = 0	0.936352	4	0.92	All Period Deviations = 0	0.976396	4	0.91	All Period Deviations = 0	0.097209	4	1.00

		All Cohort Deviations = 0	4.378705	6	0.63	All Cohort Deviations = 0	3.757362	6	0.71	All Cohort Deviations = 0	0.032707	6	1.00
		All Period RR = 1	360.1767	5	< 0.00	All Period RR = 1	225.8538	5	< 0.00	All Period RR = 1	0.470129	5	0.99
		All Cohort RR = 1	367.5304	7	< 0.00	All Cohort RR = 1	207.8908	7	< 0.00	All Cohort RR = 1	1.035915	7	0.99
		All Local Drifts = Net Drift	3.852925	3	0.28	All Local Drifts = Net Drift	3.508354	3	0.32	All Local Drifts = Net Drift	0.013655	3	1.00
	Male	Net Drift = 0	144.1831	1	< 0.00	Net Drift = 0	68.13919	1	< 0.00	Net Drift = 0	0.170304	1	0.68
		All Age Deviations = 0	81.1005	1	< 0.00	All Age Deviations = 0	542.607	1	< 0.00	All Age Deviations = 0	45.70864	1	< 0.00
		All Period Deviations = 0	0.277134	4	0.99	All Period Deviations = 0	0.643385	4	0.96	All Period Deviations = 0	0.105132	4	1.00
		All Cohort Deviations = 0	2.09526	6	0.91	All Cohort Deviations = 0	1.124817	6	0.98	All Cohort Deviations = 0	0.102095	6	1.00
		All Period RR = 1	150.8795	5	< 0.00	All Period RR = 1	82.91673	5	< 0.00	All Period RR = 1	0.409109	5	1.00
		All Cohort RR = 1	155.9434	7	< 0.00	All Cohort RR = 1	76.13431	7	< 0.00	All Cohort RR = 1	0.811346	7	1.00
		All Local Drifts = Net Drift	1.915653	3	0.59	All Local Drifts = Net Drift	1.056572	3	0.79	All Local Drifts = Net Drift	0.091864	3	0.99
	Female	Net Drift = 0	201.3117	1	< 0.00	Net Drift = 0	125.5751	1	< 0.00	Net Drift = 0	0.016971	1	0.90
		All Age Deviations = 0	84.42447	1	< 0.00	All Age Deviations = 0	614.5605	1	< 0.00	All Age Deviations = 0	24.24358	1	< 0.00
		All Period Deviations = 0	1.097976	4	0.89	All Period Deviations = 0	0.463827	4	0.98	All Period Deviations = 0	0.041808	4	1.00
		All Cohort Deviations = 0	2.852853	6	0.83	All Cohort Deviations = 0	2.852804	6	0.83	All Cohort Deviations = 0	0.071134	6	1.00
		All Period RR = 1	215.6778	5	< 0.00	All Period RR = 1	146.8212	5	< 0.00	All Period RR = 1	0.100052	5	1.00
		All Cohort RR = 1	217.9086	7	< 0.00	All Cohort RR = 1	136.0496	7	< 0.00	All Cohort RR = 1	0.247791	7	1.00
		All Local Drifts = Net Drift	2.469073	3	0.48	All Local Drifts = Net Drift	2.587012	3	0.46	All Local Drifts = Net Drift	0.041463	3	1.00
Malawi	Both	Net Drift = 0	162.096	1	< 0.00	Net Drift = 0	51.74806	1	< 0.00	Net Drift = 0	0.008065	1	0.93
		All Age Deviations = 0	123.5378	1	< 0.00	All Age Deviations = 0	790.8663	1	< 0.00	All Age Deviations = 0	85.83493	1	< 0.00
		All Period Deviations = 0	0.462869	4	0.98	All Period Deviations = 0	5.949299	4	0.20	All Period Deviations = 0	0.447186	4	0.98
		All Cohort Deviations = 0	7.523633	6	0.28	All Cohort Deviations = 0	2.711549	6	0.84	All Cohort Deviations = 0	0.711541	6	0.99
		All Period RR = 1	170.0227	5	< 0.00	All Period RR = 1	75.67526	5	< 0.00	All Period RR = 1	0.480983	5	0.99
		All Cohort RR = 1	177.9495	7	< 0.00	All Cohort RR = 1	63.1423	7	< 0.00	All Cohort RR = 1	0.914599	7	1.00

		All Local Drifts = Net Drift	5.865346	3	0.12	All Local Drifts = Net Drift	1.690789	3	0.64	All Local Drifts = Net Drift	0.704844	3	0.87
	Male	Net Drift = 0	75.00618	1	< 0.00	Net Drift = 0	16.74963	1	< 0.00	Net Drift = 0	0.04046	1	0.84
		All Age Deviations = 0	60.25402	1	< 0.00	All Age Deviations = 0	352.5424	1	< 0.00	All Age Deviations = 0	57.62886	1	< 0.00
		All Period Deviations = 0	0.311064	4	0.99	All Period Deviations = 0	0.48755	4	0.97	All Period Deviations = 0	0.479843	4	0.98
		All Cohort Deviations = 0	4.045169	6	0.67	All Cohort Deviations = 0	0.94799	6	0.99	All Cohort Deviations = 0	0.800573	6	0.99
		All Period RR = 1	77.6217	5	< 0.00	All Period RR = 1	20.47667	5	< 0.00	All Period RR = 1	0.778346	5	0.98
		All Cohort RR = 1	82.9511	7	< 0.00	All Cohort RR = 1	20.32249	7	< 0.00	All Cohort RR = 1	2.082489	7	0.96
		All Local Drifts = Net Drift	2.888361	3	0.41	All Local Drifts = Net Drift	0.134145	3	0.99	All Local Drifts = Net Drift	0.796592	3	0.85
	Female	Net Drift = 0	87.83027	1	< 0.00	Net Drift = 0	37.10618	1	< 0.00	Net Drift = 0	0.317713	1	0.57
		All Age Deviations = 0	62.98838	1	< 0.00	All Age Deviations = 0	438.4749	1	< 0.00	All Age Deviations = 0	27.81672	1	< 0.00
		All Period Deviations = 0	0.459307	4	0.98	All Period Deviations = 0	8.184467	4	0.09	All Period Deviations = 0	0.157467	4	1.00
		All Cohort Deviations = 0	3.431214	6	0.75	All Cohort Deviations = 0	3.02828	6	0.81	All Cohort Deviations = 0	0.095277	6	1.00
		All Period RR = 1	93.35242	5	< 0.00	All Period RR = 1	62.13614	5	< 0.00	All Period RR = 1	0.39088	5	1.00
		All Cohort RR = 1	95.34499	7	< 0.00	All Cohort RR = 1	46.71904	7	< 0.00	All Cohort RR = 1	1.054286	7	0.99
		All Local Drifts = Net Drift	2.867513	3	0.41	All Local Drifts = Net Drift	2.730015	3	0.44	All Local Drifts = Net Drift	0.041196	3	1.00
Mali	Both	Net Drift = 0	256.1603	1	< 0.00	Net Drift = 0	143.534	1	< 0.00	Net Drift = 0	0.073386	1	0.79
		All Age Deviations = 0	94.97973	1	< 0.00	All Age Deviations = 0	442.9263	1	< 0.00	All Age Deviations = 0	25.3812	1	< 0.00
		All Period Deviations = 0	0.162811	4	1.00	All Period Deviations = 0	4.237841	4	0.37	All Period Deviations = 0	0.089951	4	1.00
		All Cohort Deviations = 0	5.281877	6	0.51	All Cohort Deviations = 0	1.193528	6	0.98	All Cohort Deviations = 0	0.19793	6	1.00
		All Period RR = 1	263.9868	5	< 0.00	All Period RR = 1	186.0328	5	< 0.00	All Period RR = 1	0.148348	5	1.00
		All Cohort RR = 1	286.0237	7	< 0.00	All Cohort RR = 1	158.0899	7	< 0.00	All Cohort RR = 1	0.213363	7	1.00
		All Local Drifts = Net Drift	4.199306	3	0.24	All Local Drifts = Net Drift	0.996587	3	0.80	All Local Drifts = Net Drift	0.183992	3	0.98
	Male	Net Drift = 0	111.2471	1	< 0.00	Net Drift = 0	62.38689	1	< 0.00	Net Drift = 0	0.097625	1	0.75
		All Age Deviations = 0	48.68652	1	< 0.00	All Age Deviations = 0	187.6413	1	< 0.00	All Age Deviations = 0	11.54206	1	< 0.00

		All Period Deviations = 0	0.12019	4	1.00	All Period Deviations = 0	2.364682	4	0.67	All Period Deviations = 0	0.090175	4	1.00
		All Cohort Deviations = 0	3.917597	6	0.69	All Cohort Deviations = 0	1.643289	6	0.95	All Cohort Deviations = 0	0.056756	6	1.00
		All Period RR = 1	115.2623	5	< 0.00	All Period RR = 1	81.13502	5	< 0.00	All Period RR = 1	0.283846	5	1.00
		All Cohort RR = 1	120.572	7	< 0.00	All Cohort RR = 1	71.85042	7	< 0.00	All Cohort RR = 1	0.17505	7	1.00
		All Local Drifts = Net Drift	3.754577	3	0.29	All Local Drifts = Net Drift	1.567178	3	0.67	All Local Drifts = Net Drift	0.022118	3	1.00
	Female	Net Drift = 0	146.7686	1	< 0.00	Net Drift = 0	81.89942	1	< 0.00	Net Drift = 0	0.000341	1	0.99
		All Age Deviations = 0	47.9846	1	< 0.00	All Age Deviations = 0	257.7917	1	< 0.00	All Age Deviations = 0	12.83221	1	< 0.00
		All Period Deviations = 0	0.063009	4	1.00	All Period Deviations = 0	1.888705	4	0.76	All Period Deviations = 0	0.15135	4	1.00
		All Cohort Deviations = 0	2.099251	6	0.91	All Cohort Deviations = 0	1.520596	6	0.96	All Cohort Deviations = 0	0.304359	6	1.00
		All Period RR = 1	150.0061	5	< 0.00	All Period RR = 1	105.8804	5	< 0.00	All Period RR = 1	0.173034	5	1.00
		All Cohort RR = 1	172.8358	7	< 0.00	All Cohort RR = 1	87.8609	7	< 0.00	All Cohort RR = 1	0.372769	7	1.00
		All Local Drifts = Net Drift	1.137063	3	0.77	All Local Drifts = Net Drift	1.193345	3	0.75	All Local Drifts = Net Drift	0.303102	3	0.96
Mauritania	Both	Net Drift = 0	76.59031	1	< 0.00	Net Drift = 0	42.44335	1	< 0.00	Net Drift = 0	0.321255	1	0.57
		All Age Deviations = 0	21.78773	1	< 0.00	All Age Deviations = 0	103.6453	1	< 0.00	All Age Deviations = 0	4.000079	1	0.05
		All Period Deviations = 0	0.08986	4	1.00	All Period Deviations = 0	1.154653	4	0.89	All Period Deviations = 0	0.027364	4	1.00
		All Cohort Deviations = 0	1.70733	6	0.94	All Cohort Deviations = 0	1.152404	6	0.98	All Cohort Deviations = 0	0.205988	6	1.00
		All Period RR = 1	78.23911	5	< 0.00	All Period RR = 1	53.08286	5	< 0.00	All Period RR = 1	0.338616	5	1.00
		All Cohort RR = 1	84.34608	7	< 0.00	All Cohort RR = 1	47.61889	7	< 0.00	All Cohort RR = 1	0.386896	7	1.00
		All Local Drifts = Net Drift	1.225566	3	0.75	All Local Drifts = Net Drift	0.715585	3	0.87	All Local Drifts = Net Drift	0.167943	3	0.98
	Male	Net Drift = 0	33.98114	1	< 0.00	Net Drift = 0	19.75631	1	< 0.00	Net Drift = 0	0.008879	1	0.92
		All Age Deviations = 0	10.96926	1	< 0.00	All Age Deviations = 0	53.41207	1	< 0.00	All Age Deviations = 0	2.617082	1	0.11
		All Period Deviations = 0	0.061704	4	1.00	All Period Deviations = 0	0.343403	4	0.99	All Period Deviations = 0	0.151923	4	1.00
		All Cohort Deviations = 0	1.133944	6	0.98	All Cohort Deviations = 0	0.532205	6	1.00	All Cohort Deviations = 0	0.094945	6	1.00
		All Period RR = 1	34.8958	5	< 0.00	All Period RR = 1	23.88275	5	< 0.00	All Period RR = 1	0.163697	5	1.00

		All Cohort RR = 1	36.76864	7	< 0.00	All Cohort RR = 1	22.35816	7	< 0.00	All Cohort RR = 1	0.09495	7	1.00
		All Local Drifts = Net Drift	0.87692	3	0.83	All Local Drifts = Net Drift	0.27638	3	0.96	All Local Drifts = Net Drift	0.025434	3	1.00
	Female	Net Drift = 0	42.95292	1	< 0.00	Net Drift = 0	23.24172	1	< 0.00	Net Drift = 0	0.158188	1	0.69
		All Age Deviations = 0	11.34902	1	< 0.00	All Age Deviations = 0	50.62012	1	< 0.00	All Age Deviations = 0	2.068183	1	0.15
		All Period Deviations = 0	0.069328	4	1.00	All Period Deviations = 0	1.016232	4	0.91	All Period Deviations = 0	0.170811	4	1.00
		All Cohort Deviations = 0	0.827572	6	0.99	All Cohort Deviations = 0	0.76242	6	0.99	All Cohort Deviations = 0	0.270073	6	1.00
		All Period RR = 1	43.77254	5	< 0.00	All Period RR = 1	30.62054	5	< 0.00	All Period RR = 1	0.25325	5	1.00
		All Cohort RR = 1	49.45306	7	< 0.00	All Cohort RR = 1	26.19585	7	< 0.00	All Cohort RR = 1	0.286795	7	1.00
		All Local Drifts = Net Drift	0.619001	3	0.89	All Local Drifts = Net Drift	0.590579	3	0.90	All Local Drifts = Net Drift	0.197634	3	0.98
Morocco	Both	Net Drift = 0	682.4401	1	< 0.00	Net Drift = 0	509.8891	1	< 0.00	Net Drift = 0	0.07177	1	0.79
		All Age Deviations = 0	279.3648	1	< 0.00	All Age Deviations = 0	1342.498	1	< 0.00	All Age Deviations = 0	29.12629	1	< 0.00
		All Period Deviations = 0	0.849109	4	0.93	All Period Deviations = 0	0.822454	4	0.94	All Period Deviations = 0	0.058726	4	1.00
		All Cohort Deviations = 0	10.36164	6	0.11	All Cohort Deviations = 0	12.76548	6	0.05	All Cohort Deviations = 0	0.066145	6	1.00
		All Period RR = 1	688.4811	5	< 0.00	All Period RR = 1	533.2487	5	< 0.00	All Period RR = 1	0.165274	5	1.00
		All Cohort RR = 1	756.0098	7	< 0.00	All Cohort RR = 1	572.2163	7	< 0.00	All Cohort RR = 1	0.377354	7	1.00
		All Local Drifts = Net Drift	9.863302	3	0.02	All Local Drifts = Net Drift	11.89764	3	0.01	All Local Drifts = Net Drift	0.05209	3	1.00
	Male	Net Drift = 0	141.9199	1	< 0.00	Net Drift = 0	121.9065	1	< 0.00	Net Drift = 0	0.04087	1	0.84
		All Age Deviations = 0	140.9494	1	< 0.00	All Age Deviations = 0	531.1932	1	< 0.00	All Age Deviations = 0	12.8464	1	< 0.00
		All Period Deviations = 0	0.714522	4	0.95	All Period Deviations = 0	0.250448	4	0.99	All Period Deviations = 0	0.029293	4	1.00
		All Cohort Deviations = 0	1.064875	6	0.98	All Cohort Deviations = 0	1.397095	6	0.97	All Cohort Deviations = 0	0.069623	6	1.00
		All Period RR = 1	144.0167	5	< 0.00	All Period RR = 1	130.8082	5	< 0.00	All Period RR = 1	0.077309	5	1.00
		All Cohort RR = 1	158.7542	7	< 0.00	All Cohort RR = 1	135.5136	7	< 0.00	All Cohort RR = 1	0.285599	7	1.00
		All Local Drifts = Net Drift	0.97594	3	0.81	All Local Drifts = Net Drift	1.053341	3	0.79	All Local Drifts = Net Drift	0.048034	3	1.00
	Female	Net Drift = 0	619.0486	1	< 0.00	Net Drift = 0	427.1198	1	< 0.00	Net Drift = 0	0.015611	1	0.90

		All Age Deviations = 0	141.5084	1	< 0.00	All Age Deviations = 0	818.4765	1	< 0.00	All Age Deviations = 0	20.66648	1	< 0.00
		All Period Deviations = 0	0.09133	4	1.00	All Period Deviations = 0	1.667013	4	0.80	All Period Deviations = 0	0.038367	4	1.00
		All Cohort Deviations = 0	12.59214	6	0.05	All Cohort Deviations = 0	14.63745	6	0.02	All Cohort Deviations = 0	0.040383	6	1.00
		All Period RR = 1	623.9748	5	< 0.00	All Period RR = 1	443.2558	5	< 0.00	All Period RR = 1	0.064195	5	1.00
		All Cohort RR = 1	686.4372	7	< 0.00	All Cohort RR = 1	483.128	7	< 0.00	All Cohort RR = 1	0.116041	7	1.00
		All Local Drifts = Net Drift	12.19784	3	0.01	All Local Drifts = Net Drift	14.10403	3	< 0.00	All Local Drifts = Net Drift	0.033978	3	1.00
Mozambique	Both	Net Drift = 0	611.7923	1	< 0.00	Net Drift = 0	550.4993	1	< 0.00	Net Drift = 0	0.037338	1	0.85
		All Age Deviations = 0	151.1963	1	< 0.00	All Age Deviations = 0	1011.94	1	< 0.00	All Age Deviations = 0	42.3632	1	< 0.00
		All Period Deviations = 0	0.346349	4	0.99	All Period Deviations = 0	0.342141	4	0.99	All Period Deviations = 0	0.046815	4	1.00
		All Cohort Deviations = 0	19.37244	6	< 0.00	All Cohort Deviations = 0	10.48893	6	0.11	All Cohort Deviations = 0	0.172541	6	1.00
		All Period RR = 1	636.8773	5	< 0.00	All Period RR = 1	637.4178	5	< 0.00	All Period RR = 1	0.072447	5	1.00
		All Cohort RR = 1	685.3033	7	< 0.00	All Cohort RR = 1	632.9677	7	< 0.00	All Cohort RR = 1	0.189504	7	1.00
		All Local Drifts = Net Drift	17.84732	3	< 0.00	All Local Drifts = Net Drift	9.707348	3	0.02	All Local Drifts = Net Drift	0.166786	3	0.98
	Male	Net Drift = 0	214.5275	1	< 0.00	Net Drift = 0	191.9066	1	< 0.00	Net Drift = 0	0.031691	1	0.86
		All Age Deviations = 0	83.99215	1	< 0.00	All Age Deviations = 0	529.9536	1	< 0.00	All Age Deviations = 0	31.30348	1	< 0.00
		All Period Deviations = 0	0.522282	4	0.97	All Period Deviations = 0	0.812893	4	0.94	All Period Deviations = 0	0.007588	4	1.00
		All Cohort Deviations = 0	5.28329	6	0.51	All Cohort Deviations = 0	3.775492	6	0.71	All Cohort Deviations = 0	0.085608	6	1.00
		All Period RR = 1	219.8713	5	< 0.00	All Period RR = 1	226.2718	5	< 0.00	All Period RR = 1	0.034785	5	1.00
		All Cohort RR = 1	238.6056	7	< 0.00	All Cohort RR = 1	227.4235	7	< 0.00	All Cohort RR = 1	0.088452	7	1.00
		All Local Drifts = Net Drift	4.861584	3	0.18	All Local Drifts = Net Drift	3.170688	3	0.37	All Local Drifts = Net Drift	0.06463	3	1.00
	Female	Net Drift = 0	420.3343	1	< 0.00	Net Drift = 0	369.7373	1	< 0.00	Net Drift = 0	0.001262	1	0.97
		All Age Deviations = 0	65.07414	1	< 0.00	All Age Deviations = 0	479.504	1	< 0.00	All Age Deviations = 0	11.65009	1	< 0.00
		All Period Deviations = 0	0.095183	4	1.00	All Period Deviations = 0	0.1317	4	1.00	All Period Deviations = 0	0.053294	4	1.00
		All Cohort Deviations = 0	17.78074	6	0.01	All Cohort Deviations = 0	12.40495	6	0.05	All Cohort Deviations = 0	0.051704	6	1.00

		All Period RR = 1	444.0525	5	< 0.00	All Period RR = 1	423.4706	5	< 0.00	All Period RR = 1	0.053296	5	1.00
		All Cohort RR = 1	475.9109	7	< 0.00	All Cohort RR = 1	419.9244	7	< 0.00	All Cohort RR = 1	0.052203	7	1.00
		All Local Drifts = Net Drift	16.15987	3	< 0.00	All Local Drifts = Net Drift	11.47173	3	0.01	All Local Drifts = Net Drift	0.039431	3	1.00
Namibia	Both	Net Drift = 0	4.483956	1	0.03	Net Drift = 0	0.292916	1	0.59	Net Drift = 0	1.45E-06	1	1.00
		All Age Deviations = 0	29.2347	1	< 0.00	All Age Deviations = 0	135.3089	1	< 0.00	All Age Deviations = 0	4.235742	1	0.04
		All Period Deviations = 0	0.141917	4	1.00	All Period Deviations = 0	1.275277	4	0.87	All Period Deviations = 0	0.079012	4	1.00
		All Cohort Deviations = 0	3.025659	6	0.81	All Cohort Deviations = 0	1.917377	6	0.93	All Cohort Deviations = 0	0.05083	6	1.00
		All Period RR = 1	4.560205	5	0.47	All Period RR = 1	1.959119	5	0.85	All Period RR = 1	0.080621	5	1.00
		All Cohort RR = 1	7.332616	7	0.40	All Cohort RR = 1	3.084475	7	0.88	All Cohort RR = 1	0.050997	7	1.00
		All Local Drifts = Net Drift	2.626137	3	0.45	All Local Drifts = Net Drift	1.28137	3	0.73	All Local Drifts = Net Drift	0.027207	3	1.00
	Male	Net Drift = 0	1.846854	1	0.17	Net Drift = 0	0.60699	1	0.44	Net Drift = 0	0.000154	1	0.99
		All Age Deviations = 0	12.85469	1	< 0.00	All Age Deviations = 0	69.15306	1	< 0.00	All Age Deviations = 0	2.825146	1	0.09
		All Period Deviations = 0	0.05159	4	1.00	All Period Deviations = 0	0.091506	4	1.00	All Period Deviations = 0	0.038497	4	1.00
		All Cohort Deviations = 0	1.297614	6	0.97	All Cohort Deviations = 0	0.574463	6	1.00	All Cohort Deviations = 0	0.041512	6	1.00
		All Period RR = 1	1.865523	5	0.87	All Period RR = 1	0.833639	5	0.97	All Period RR = 1	0.038713	5	1.00
		All Cohort RR = 1	3.176224	7	0.87	All Cohort RR = 1	1.745292	7	0.97	All Cohort RR = 1	0.04736	7	1.00
		All Local Drifts = Net Drift	1.093463	3	0.78	All Local Drifts = Net Drift	0.31334	3	0.96	All Local Drifts = Net Drift	0.014505	3	1.00
	Female	Net Drift = 0	2.727612	1	0.10	Net Drift = 0	4.61E-05	1	0.99	Net Drift = 0	0.00067	1	0.98
		All Age Deviations = 0	17.55104	1	< 0.00	All Age Deviations = 0	69.72645	1	< 0.00	All Age Deviations = 0	1.978159	1	0.16
		All Period Deviations = 0	0.11267	4	1.00	All Period Deviations = 0	2.044878	4	0.73	All Period Deviations = 0	0.077588	4	1.00
		All Cohort Deviations = 0	1.764814	6	0.94	All Cohort Deviations = 0	1.765966	6	0.94	All Cohort Deviations = 0	0.230072	6	1.00
		All Period RR = 1	2.811602	5	0.73	All Period RR = 1	2.140615	5	0.83	All Period RR = 1	0.079221	5	1.00
		All Cohort RR = 1	4.230822	7	0.75	All Cohort RR = 1	1.977975	7	0.96	All Cohort RR = 1	0.230697	7	1.00
		All Local Drifts = Net Drift	1.524843	3	0.68	All Local Drifts = Net Drift	1.305386	3	0.73	All Local Drifts = Net Drift	0.099897	3	0.99

Niger	Both	Net Drift = 0	503.0225	1	< 0.00	Net Drift = 0	348.4525	1	< 0.00	Net Drift = 0	0.042997	1	0.84
		All Age Deviations = 0	46.06098	1	< 0.00	All Age Deviations = 0	417.4766	1	< 0.00	All Age Deviations = 0	26.58374	1	0.00
		All Period Deviations = 0	0.299018	4	0.99	All Period Deviations = 0	0.236889	4	0.99	All Period Deviations = 0	0.063557	4	1.00
		All Cohort Deviations = 0	16.04341	6	0.01	All Cohort Deviations = 0	15.31626	6	0.02	All Cohort Deviations = 0	0.180617	6	1.00
		All Period RR = 1	523.4361	5	< 0.00	All Period RR = 1	405.6126	5	< 0.00	All Period RR = 1	0.089799	5	1.00
		All Cohort RR = 1	633.392	7	< 0.00	All Cohort RR = 1	367.8111	7	< 0.00	All Cohort RR = 1	0.197262	7	1.00
		All Local Drifts = Net Drift	7.652574	3	0.05	All Local Drifts = Net Drift	12.39152	3	0.01	All Local Drifts = Net Drift	0.166444	3	0.98
	Male	Net Drift = 0	239.3132	1	< 0.00	Net Drift = 0	168.2557	1	< 0.00	Net Drift = 0	0.001978	1	0.96
		All Age Deviations = 0	27.02368	1	< 0.00	All Age Deviations = 0	156.84	1	< 0.00	All Age Deviations = 0	12.87351	1	< 0.00
		All Period Deviations = 0	0.203904	4	1.00	All Period Deviations = 0	0.159689	4	1.00	All Period Deviations = 0	0.059587	4	1.00
		All Cohort Deviations = 0	6.832041	6	0.34	All Cohort Deviations = 0	3.391472	6	0.76	All Cohort Deviations = 0	0.061123	6	1.00
		All Period RR = 1	249.8801	5	< 0.00	All Period RR = 1	200.2859	5	< 0.00	All Period RR = 1	0.059603	5	1.00
		All Cohort RR = 1	288.9644	7	< 0.00	All Cohort RR = 1	181.3643	7	< 0.00	All Cohort RR = 1	0.082561	7	1.00
		All Local Drifts = Net Drift	3.312498	3	0.35	All Local Drifts = Net Drift	2.218494	3	0.53	All Local Drifts = Net Drift	0.05208	3	1.00
	Female	Net Drift = 0	252.2737	1	< 0.00	Net Drift = 0	183.873	1	< 0.00	Net Drift = 0	0.050519	1	0.82
		All Age Deviations = 0	22.63364	1	< 0.00	All Age Deviations = 0	263.597	1	< 0.00	All Age Deviations = 0	12.95436	1	< 0.00
		All Period Deviations = 0	0.108237	4	1.00	All Period Deviations = 0	0.829462	4	0.93	All Period Deviations = 0	0.061496	4	1.00
		All Cohort Deviations = 0	11.01627	6	0.09	All Cohort Deviations = 0	15.2394	6	0.02	All Cohort Deviations = 0	0.244266	6	1.00
		All Period RR = 1	261.6185	5	< 0.00	All Period RR = 1	210.7174	5	< 0.00	All Period RR = 1	0.095501	5	1.00
		All Cohort RR = 1	343.1994	7	< 0.00	All Cohort RR = 1	192.1588	7	< 0.00	All Cohort RR = 1	0.248566	7	1.00
		All Local Drifts = Net Drift	5.299559	3	0.15	All Local Drifts = Net Drift	13.57093	3	< 0.00	All Local Drifts = Net Drift	0.214181	3	0.98
Nigeria	Both	Net Drift = 0	166.0801	1	< 0.00	Net Drift = 0	3.63289	1	0.06	Net Drift = 0	1.73461	1	0.19
		All Age Deviations = 0	243.3326	1	< 0.00	All Age Deviations = 0	1931.71	1	< 0.00	All Age Deviations = 0	326.4344	1	< 0.00
		All Period Deviations = 0	9.218355	4	0.06	All Period Deviations = 0	234.597	4	< 0.00	All Period Deviations = 0	2.843896	4	0.58

		All Cohort Deviations = 0	53.64606	6	< 0.00	All Cohort Deviations = 0	30.81644	6	< 0.00	All Cohort Deviations = 0	2.181285	6	0.90
		All Period RR = 1	178.9245	5	< 0.00	All Period RR = 1	243.3993	5	< 0.00	All Period RR = 1	8.609647	5	0.13
		All Cohort RR = 1	225.4356	7	< 0.00	All Cohort RR = 1	30.99668	7	< 0.00	All Cohort RR = 1	14.65475	7	0.04
		All Local Drifts = Net Drift	42.11978	3	< 0.00	All Local Drifts = Net Drift	27.18776	3	< 0.00	All Local Drifts = Net Drift	1.486118	3	0.69
	Male	Net Drift = 0	153.5557	1	< 0.00	Net Drift = 0	4.043068	1	0.04	Net Drift = 0	8.716968	1	< 0.00
		All Age Deviations = 0	306.9223	1	< 0.00	All Age Deviations = 0	1463.427	1	3.46685863686803e-320	All Age Deviations = 0	76.21562	1	< 0.00
		All Period Deviations = 0	9.856018	4	0.04	All Period Deviations = 0	271.4466	4	< 0.00	All Period Deviations = 0	3.61752	4	0.46
		All Cohort Deviations = 0	54.8201	6	< 0.00	All Cohort Deviations = 0	41.74527	6	< 0.00	All Cohort Deviations = 0	4.695134	6	0.58
		All Period RR = 1	167.5845	5	< 0.00	All Period RR = 1	331.8919	5	< 0.00	All Period RR = 1	23.98186	5	< 0.00
		All Cohort RR = 1	215.0546	7	< 0.00	All Cohort RR = 1	61.70151	7	< 0.00	All Cohort RR = 1	75.92535	7	< 0.00
		All Local Drifts = Net Drift	43.18277	3	< 0.00	All Local Drifts = Net Drift	39.14845	3	< 0.00	All Local Drifts = Net Drift	4.196615	3	0.24
	Female	Net Drift = 0	166.4271	1	< 0.00	Net Drift = 0	15.0864	1	< 0.00	Net Drift = 0	0.912779	1	0.34
		All Age Deviations = 0	189.1261	1	< 0.00	All Age Deviations = 0	1543.125	1	< 0.00	All Age Deviations = 0	267.4997	1	< 0.00
		All Period Deviations = 0	13.89504	4	0.01	All Period Deviations = 0	143.6913	4	< 0.00	All Period Deviations = 0	0.432895	4	0.98
		All Cohort Deviations = 0	52.9014	6	< 0.00	All Cohort Deviations = 0	17.24199	6	0.01	All Cohort Deviations = 0	1.451871	6	0.96
		All Period RR = 1	186.1532	5	< 0.00	All Period RR = 1	143.8642	5	< 0.00	All Period RR = 1	1.653205	5	0.89
		All Cohort RR = 1	223.2256	7	< 0.00	All Cohort RR = 1	28.15256	7	< 0.00	All Cohort RR = 1	8.805194	7	0.27
		All Local Drifts = Net Drift	43.56422	3	< 0.00	All Local Drifts = Net Drift	13.96734	3	< 0.00	All Local Drifts = Net Drift	1.422744	3	0.70
Rwanda	Both	Net Drift = 0	109.5592	1	< 0.00	Net Drift = 0	0.062049	1	0.80	Net Drift = 0	0.012347	1	0.91
		All Age Deviations = 0	79.18587	1	< 0.00	All Age Deviations = 0	698.7623	1	< 0.00	All Age Deviations = 0	37.66942	1	< 0.00
		All Period Deviations = 0	0.375475	4	0.98	All Period Deviations = 0	14.81549	4	0.01	All Period Deviations = 0	1.521664	4	0.82
		All Cohort Deviations = 0	2.886611	6	0.82	All Cohort Deviations = 0	2.843815	6	0.83	All Cohort Deviations = 0	0.620768	6	1.00
		All Period RR = 1	112.9071	5	< 0.00	All Period RR = 1	14.8601	5	0.01	All Period RR = 1	1.670924	5	0.89
		All Cohort RR = 1	115.8099	7	< 0.00	All Cohort RR = 1	3.228126	7	0.86	All Cohort RR = 1	0.977852	7	1.00

		All Local Drifts = Net Drift	2.474871	3	0.48	All Local Drifts = Net Drift	2.289001	3	0.51	All Local Drifts = Net Drift	0.558869	3	0.91
	Male	Net Drift = 0	40.97674	1	< 0.00	Net Drift = 0	0.958055	1	0.33	Net Drift = 0	0.099771	1	0.75
		All Age Deviations = 0	36.69303	1	< 0.00	All Age Deviations = 0	239.8371	1	< 0.00	All Age Deviations = 0	24.56752	1	< 0.00
		All Period Deviations = 0	0.13582	4	1.00	All Period Deviations = 0	1.701147	4	0.79	All Period Deviations = 0	1.898742	4	0.75
		All Cohort Deviations = 0	0.740139	6	0.99	All Cohort Deviations = 0	0.861939	6	0.99	All Cohort Deviations = 0	0.553464	6	1.00
		All Period RR = 1	42.1141	5	< 0.00	All Period RR = 1	3.092083	5	0.69	All Period RR = 1	1.898757	5	0.86
		All Cohort RR = 1	42.92929	7	< 0.00	All Cohort RR = 1	1.525656	7	0.98	All Cohort RR = 1	1.466285	7	0.98
		All Local Drifts = Net Drift	0.53895	3	0.91	All Local Drifts = Net Drift	0.564272	3	0.90	All Local Drifts = Net Drift	0.526575	3	0.91
	Female	Net Drift = 0	67.78291	1	< 0.00	Net Drift = 0	0.302759	1	0.58	Net Drift = 0	0.05591	1	0.81
		All Age Deviations = 0	42.86166	1	< 0.00	All Age Deviations = 0	461.6963	1	< 0.00	All Age Deviations = 0	13.80283	1	< 0.00
		All Period Deviations = 0	0.323293	4	0.99	All Period Deviations = 0	14.98491	4	< 0.00	All Period Deviations = 0	0.133702	4	1.00
		All Cohort Deviations = 0	2.11615	6	0.91	All Cohort Deviations = 0	1.41884	6	0.96	All Cohort Deviations = 0	0.27796	6	1.00
		All Period RR = 1	70.0287	5	< 0.00	All Period RR = 1	15.38196	5	0.01	All Period RR = 1	0.332447	5	1.00
		All Cohort RR = 1	72.37387	7	< 0.00	All Cohort RR = 1	2.095814	7	0.95	All Cohort RR = 1	1.402975	7	0.99
		All Local Drifts = Net Drift	1.973009	3	0.58	All Local Drifts = Net Drift	1.102171	3	0.78	All Local Drifts = Net Drift	0.225485	3	0.97
Sao Tome and Principe	Both	Net Drift = 0	1.344233	1	0.25	Net Drift = 0	0.758782	1	0.38	Net Drift = 0	0.015631	1	0.90
		All Age Deviations = 0	1.850807	1	0.17	All Age Deviations = 0	9.075871	1	< 0.00	All Age Deviations = 0	0.283581	1	0.59
		All Period Deviations = 0	0.03325	4	1.00	All Period Deviations = 0	0.153481	4	1.00	All Period Deviations = 0	0.113858	4	1.00
		All Cohort Deviations = 0	0.062122	6	1.00	All Cohort Deviations = 0	0.083537	6	1.00	All Cohort Deviations = 0	1.094178	6	0.98
		All Period RR = 1	1.417409	5	0.92	All Period RR = 1	1.122717	5	0.95	All Period RR = 1	0.199277	5	1.00
		All Cohort RR = 1	1.554602	7	0.98	All Cohort RR = 1	1.011677	7	0.99	All Cohort RR = 1	1.094882	7	0.99
		All Local Drifts = Net Drift	0.025307	3	1.00	All Local Drifts = Net Drift	0.043421	3	1.00	All Local Drifts = Net Drift	0.549018	3	0.91
	Male	Net Drift = 0	0.348056	1	0.56	Net Drift = 0	0.15568	1	0.69	Net Drift = 0	0.033408	1	0.85
		All Age Deviations = 0	0.748446	1	0.39	All Age Deviations = 0	3.771697	1	0.05	All Age Deviations = 0	0.000101	1	0.99

		All Period Deviations = 0	0.058338	4	1.00	All Period Deviations = 0	0.055224	4	1.00	All Period Deviations = 0	0.000593	4	1.00
		All Cohort Deviations = 0	0.05499	6	1.00	All Cohort Deviations = 0	0.052153	6	1.00	All Cohort Deviations = 0	0.002139	6	1.00
		All Period RR = 1	0.435818	5	0.99	All Period RR = 1	0.254924	5	1.00	All Period RR = 1	0.034108	5	1.00
		All Cohort RR = 1	0.42342	7	1.00	All Cohort RR = 1	0.196767	7	1.00	All Cohort RR = 1	0.034729	7	1.00
		All Local Drifts = Net Drift	0.007605	3	1.00	All Local Drifts = Net Drift	0.036718	3	1.00	All Local Drifts = Net Drift	0.002041	3	1.00
	Female	Net Drift = 0	0.729857	1	0.39	Net Drift = 0	0.37202	1	0.54	Net Drift = 0	0.111532	1	0.74
		All Age Deviations = 0	1.225698	1	0.27	All Age Deviations = 0	6.125933	1	0.01	All Age Deviations = 0	0.23276	1	0.63
		All Period Deviations = 0	0.068719	4	1.00	All Period Deviations = 0	0.071604	4	1.00	All Period Deviations = 0	0.314636	4	0.99
		All Cohort Deviations = 0	0.076033	6	1.00	All Cohort Deviations = 0	0.027155	6	1.00	All Cohort Deviations = 0	0.552046	6	1.00
		All Period RR = 1	0.835284	5	0.97	All Period RR = 1	0.539841	5	0.99	All Period RR = 1	0.491091	5	0.99
		All Cohort RR = 1	0.81963	7	1.00	All Cohort RR = 1	0.46723	7	1.00	All Cohort RR = 1	0.832209	7	1.00
		All Local Drifts = Net Drift	0.048871	3	1.00	All Local Drifts = Net Drift	0.011624	3	1.00	All Local Drifts = Net Drift	0.452894	3	0.93
Senegal	Both	Net Drift = 0	190.9613	1	< 0.00	Net Drift = 0	126.0756	1	< 0.00	Net Drift = 0	0.003374	1	0.95
		All Age Deviations = 0	79.88499	1	< 0.00	All Age Deviations = 0	452.0369	1	< 0.00	All Age Deviations = 0	21.9122	1	< 0.00
		All Period Deviations = 0	0.999389	4	0.91	All Period Deviations = 0	1.315348	4	0.86	All Period Deviations = 0	0.018025	4	1.00
		All Cohort Deviations = 0	3.890233	6	0.69	All Cohort Deviations = 0	1.159781	6	0.98	All Cohort Deviations = 0	0.080123	6	1.00
		All Period RR = 1	197.3331	5	< 0.00	All Period RR = 1	144.936	5	< 0.00	All Period RR = 1	0.020475	5	1.00
		All Cohort RR = 1	218.4992	7	< 0.00	All Cohort RR = 1	136.9506	7	< 0.00	All Cohort RR = 1	0.081197	7	1.00
		All Local Drifts = Net Drift	2.871872	3	0.41	All Local Drifts = Net Drift	1.140755	3	0.77	All Local Drifts = Net Drift	0.036829	3	1.00
	Male	Net Drift = 0	61.27649	1	< 0.00	Net Drift = 0	39.63049	1	< 0.00	Net Drift = 0	0.00413	1	0.95
		All Age Deviations = 0	39.94258	1	< 0.00	All Age Deviations = 0	207.675	1	< 0.00	All Age Deviations = 0	11.28428	1	< 0.00
		All Period Deviations = 0	0.867067	4	0.93	All Period Deviations = 0	0.723879	4	0.95	All Period Deviations = 0	0.032164	4	1.00
		All Cohort Deviations = 0	1.347304	6	0.97	All Cohort Deviations = 0	0.127079	6	1.00	All Cohort Deviations = 0	0.262348	6	1.00
		All Period RR = 1	65.03159	5	< 0.00	All Period RR = 1	46.0717	5	< 0.00	All Period RR = 1	0.055849	5	1.00

		All Cohort RR = 1	68.15198	7	< 0.00	All Cohort RR = 1	42.87635	7	< 0.00	All Cohort RR = 1	0.295427	7	1.00
		All Local Drifts = Net Drift	1.167796	3	0.76	All Local Drifts = Net Drift	0.1226	3	0.99	All Local Drifts = Net Drift	0.196691	3	0.98
	Female	Net Drift = 0	132.7614	1	< 0.00	Net Drift = 0	90.74177	1	< 0.00	Net Drift = 0	0.016817	1	0.90
		All Age Deviations = 0	42.27403	1	< 0.00	All Age Deviations = 0	245.4535	1	< 0.00	All Age Deviations = 0	10.29137	1	< 0.00
		All Period Deviations = 0	0.310888	4	0.99	All Period Deviations = 0	0.610597	4	0.96	All Period Deviations = 0	0.031437	4	1.00
		All Cohort Deviations = 0	2.695252	6	0.85	All Cohort Deviations = 0	1.443284	6	0.96	All Cohort Deviations = 0	0.099897	6	1.00
		All Period RR = 1	135.2842	5	< 0.00	All Period RR = 1	103.8156	5	< 0.00	All Period RR = 1	0.049922	5	1.00
		All Cohort RR = 1	158.1327	7	< 0.00	All Cohort RR = 1	98.90341	7	< 0.00	All Cohort RR = 1	0.14518	7	1.00
		All Local Drifts = Net Drift	2.001763	3	0.57	All Local Drifts = Net Drift	1.399612	3	0.71	All Local Drifts = Net Drift	0.098152	3	0.99
Sierra Leone	Both	Net Drift = 0	80.62932	1	< 0.00	Net Drift = 0	68.10924	1	< 0.00	Net Drift = 0	0.00145	1	0.97
		All Age Deviations = 0	58.27797	1	< 0.00	All Age Deviations = 0	286.7614	1	< 0.00	All Age Deviations = 0	10.5463	1	< 0.00
		All Period Deviations = 0	0.05024	4	1.00	All Period Deviations = 0	0.122364	4	1.00	All Period Deviations = 0	0.084717	4	1.00
		All Cohort Deviations = 0	0.276559	6	1.00	All Cohort Deviations = 0	0.835507	6	0.99	All Cohort Deviations = 0	0.052201	6	1.00
		All Period RR = 1	81.87528	5	< 0.00	All Period RR = 1	77.23299	5	< 0.00	All Period RR = 1	0.100748	5	1.00
		All Cohort RR = 1	85.52176	7	< 0.00	All Cohort RR = 1	73.72954	7	< 0.00	All Cohort RR = 1	0.054294	7	1.00
		All Local Drifts = Net Drift	0.195588	3	0.98	All Local Drifts = Net Drift	0.681046	3	0.88	All Local Drifts = Net Drift	0.014089	3	1.00
	Male	Net Drift = 0	23.00379	1	< 0.00	Net Drift = 0	15.1894	1	< 0.00	Net Drift = 0	0.030435	1	0.86
		All Age Deviations = 0	27.4665	1	< 0.00	All Age Deviations = 0	107.5385	1	< 0.00	All Age Deviations = 0	2.897654	1	0.09
		All Period Deviations = 0	0.027524	4	1.00	All Period Deviations = 0	0.08052	4	1.00	All Period Deviations = 0	0.043489	4	1.00
		All Cohort Deviations = 0	0.137402	6	1.00	All Cohort Deviations = 0	0.582484	6	1.00	All Cohort Deviations = 0	0.0866	6	1.00
		All Period RR = 1	23.33628	5	< 0.00	All Period RR = 1	17.71934	5	< 0.00	All Period RR = 1	0.114951	5	1.00
		All Cohort RR = 1	23.95585	7	< 0.00	All Cohort RR = 1	16.47077	7	0.02	All Cohort RR = 1	0.210033	7	1.00
		All Local Drifts = Net Drift	0.085838	3	0.99	All Local Drifts = Net Drift	0.435482	3	0.93	All Local Drifts = Net Drift	0.025491	3	1.00
	Female	Net Drift = 0	59.46917	1	< 0.00	Net Drift = 0	59.0483	1	< 0.00	Net Drift = 0	0.096121	1	0.76

		All Age Deviations = 0	31.78464	1	< 0.00	All Age Deviations = 0	181.2413	1	< 0.00	All Age Deviations = 0	5.086972	1	0.02
		All Period Deviations = 0	0.092157	4	1.00	All Period Deviations = 0	0.188194	4	1.00	All Period Deviations = 0	0.054536	4	1.00
		All Cohort Deviations = 0	0.172422	6	1.00	All Cohort Deviations = 0	0.29902	6	1.00	All Cohort Deviations = 0	0.35838	6	1.00
		All Period RR = 1	60.44017	5	< 0.00	All Period RR = 1	66.32422	5	< 0.00	All Period RR = 1	0.112973	5	1.00
		All Cohort RR = 1	65.20466	7	< 0.00	All Cohort RR = 1	64.66272	7	< 0.00	All Cohort RR = 1	0.403635	7	1.00
		All Local Drifts = Net Drift	0.159329	3	0.98	All Local Drifts = Net Drift	0.250226	3	0.97	All Local Drifts = Net Drift	0.333074	3	0.95
Somalia	Both	Net Drift = 0	293.7999	1	< 0.00	Net Drift = 0	262.1465	1	< 0.00	Net Drift = 0	0.023934	1	0.88
		All Age Deviations = 0	103.1795	1	< 0.00	All Age Deviations = 0	672.0068	1	< 0.00	All Age Deviations = 0	47.213	1	< 0.00
		All Period Deviations = 0	0.242205	4	0.99	All Period Deviations = 0	0.149537	4	1.00	All Period Deviations = 0	0.035774	4	1.00
		All Cohort Deviations = 0	1.103024	6	0.98	All Cohort Deviations = 0	2.337369	6	0.89	All Cohort Deviations = 0	0.097096	6	1.00
		All Period RR = 1	307.0419	5	< 0.00	All Period RR = 1	302.1681	5	< 0.00	All Period RR = 1	0.071549	5	1.00
		All Cohort RR = 1	321.9677	7	< 0.00	All Cohort RR = 1	278.9237	7	< 0.00	All Cohort RR = 1	0.218586	7	1.00
		All Local Drifts = Net Drift	0.956121	3	0.81	All Local Drifts = Net Drift	2.139556	3	0.54	All Local Drifts = Net Drift	0.088055	3	0.99
	Male	Net Drift = 0	136.4142	1	< 0.00	Net Drift = 0	113.3541	1	< 0.00	Net Drift = 0	0.123316	1	0.73
		All Age Deviations = 0	64.36917	1	< 0.00	All Age Deviations = 0	343.5526	1	< 0.00	All Age Deviations = 0	30.90785	1	< 0.00
		All Period Deviations = 0	0.21681	4	0.99	All Period Deviations = 0	0.087248	4	1.00	All Period Deviations = 0	0.001417	4	1.00
		All Cohort Deviations = 0	0.710772	6	0.99	All Cohort Deviations = 0	0.475528	6	1.00	All Cohort Deviations = 0	0.063608	6	1.00
		All Period RR = 1	142.0218	5	< 0.00	All Period RR = 1	133.0552	5	< 0.00	All Period RR = 1	0.170511	5	1.00
		All Cohort RR = 1	149.5535	7	< 0.00	All Cohort RR = 1	123.4395	7	0.00	All Cohort RR = 1	0.178576	7	1.00
		All Local Drifts = Net Drift	0.619569	3	0.89	All Local Drifts = Net Drift	0.344133	3	0.95	All Local Drifts = Net Drift	0.043449	3	1.00
	Female	Net Drift = 0	168.4065	1	< 0.00	Net Drift = 0	150.9885	1	< 0.00	Net Drift = 0	0.027047	1	0.87
		All Age Deviations = 0	38.79032	1	< 0.00	All Age Deviations = 0	328.8783	1	< 0.00	All Age Deviations = 0	15.86033	1	< 0.00
		All Period Deviations = 0	0.124721	4	1.00	All Period Deviations = 0	0.482381	4	0.98	All Period Deviations = 0	0.030754	4	1.00
		All Cohort Deviations = 0	0.907564	6	0.99	All Cohort Deviations = 0	2.814492	6	0.83	All Cohort Deviations = 0	0.108715	6	1.00

		All Period RR = 1	177.4475	5	< 0.00	All Period RR = 1	171.5292	5	< 0.00	All Period RR = 1	0.085257	5	1.00
		All Cohort RR = 1	187.8703	7	< 0.00	All Cohort RR = 1	158.1075	7	< 0.00	All Cohort RR = 1	0.117395	7	1.00
		All Local Drifts = Net Drift	0.842631	3	0.84	All Local Drifts = Net Drift	2.70313	3	0.44	All Local Drifts = Net Drift	0.059192	3	1.00
South Africa	Both	Net Drift = 0	3.990973	1	0.05	Net Drift = 0	167.6156	1	< 0.00	Net Drift = 0	0.989709	1	0.32
		All Age Deviations = 0	183.7192	1	< 0.00	All Age Deviations = 0	2791.312	1	< 0.00	All Age Deviations = 0	75.39282	1	< 0.00
		All Period Deviations = 0	7.859674	4	0.10	All Period Deviations = 0	22.35183	4	< 0.00	All Period Deviations = 0	27.35338	4	< 0.00
		All Cohort Deviations = 0	16.6917	6	0.01	All Cohort Deviations = 0	20.13457	6	< 0.00	All Cohort Deviations = 0	21.47427	6	< 0.00
		All Period RR = 1	12.18633	5	0.03	All Period RR = 1	234.5565	5	< 0.00	All Period RR = 1	28.32895	5	< 0.00
		All Cohort RR = 1	22.39109	7	< 0.00	All Cohort RR = 1	310.1498	7	< 0.00	All Cohort RR = 1	42.53526	7	< 0.00
		All Local Drifts = Net Drift	8.566585	3	0.04	All Local Drifts = Net Drift	12.07486	3	0.01	All Local Drifts = Net Drift	20.05657	3	< 0.00
	Male	Net Drift = 0	14.63455	1	< 0.00	Net Drift = 0	186.2944	1	< 0.00	Net Drift = 0	2.343563	1	0.13
		All Age Deviations = 0	201.4062	1	< 0.00	All Age Deviations = 0	1292.481	1	< 0.00	All Age Deviations = 0	47.7812	1	< 0.00
		All Period Deviations = 0	1.435655	4	0.84	All Period Deviations = 0	7.235793	4	0.12	All Period Deviations = 0	37.08078	4	< 0.00
		All Cohort Deviations = 0	12.92797	6	0.04	All Cohort Deviations = 0	4.954917	6	0.55	All Cohort Deviations = 0	31.65312	6	< 0.00
		All Period RR = 1	17.05077	5	< 0.00	All Period RR = 1	226.6408	5	< 0.00	All Period RR = 1	37.60828	5	< 0.00
		All Cohort RR = 1	31.41519	7	< 0.00	All Cohort RR = 1	297.3395	7	< 0.00	All Cohort RR = 1	55.31735	7	< 0.00
		All Local Drifts = Net Drift	6.554319	3	0.09	All Local Drifts = Net Drift	1.919802	3	0.59	All Local Drifts = Net Drift	29.69308	3	< 0.00
	Female	Net Drift = 0	0.052674	1	0.82	Net Drift = 0	23.50152	1	< 0.00	Net Drift = 0	0.895762	1	0.34
		All Age Deviations = 0	180.3065	1	< 0.00	All Age Deviations = 0	1562.492	1	< 0.00	All Age Deviations = 0	41.61943	1	< 0.00
		All Period Deviations = 0	23.45219	4	< 0.00	All Period Deviations = 0	20.35828	4	< 0.00	All Period Deviations = 0	0.515721	4	0.97
		All Cohort Deviations = 0	16.95283	6	0.01	All Cohort Deviations = 0	12.26757	6	0.06	All Cohort Deviations = 0	0.094142	6	1.00
		All Period RR = 1	23.54084	5	< 0.00	All Period RR = 1	53.53355	5	< 0.00	All Period RR = 1	1.942711	5	0.86
		All Cohort RR = 1	17.18704	7	0.02	All Cohort RR = 1	59.95708	7	< 0.00	All Cohort RR = 1	5.152835	7	0.64
		All Local Drifts = Net Drift	8.078406	3	0.04	All Local Drifts = Net Drift	7.509334	3	0.06	All Local Drifts = Net Drift	0.064621	3	1.00

South Sudan	Both	Net Drift = 0	40.71952	1	< 0.00	Net Drift = 0	23.00679	1	< 0.00	Net Drift = 0	0.10719	1	0.74
		All Age Deviations = 0	83.72831	1	< 0.00	All Age Deviations = 0	434.9827	1	< 0.00	All Age Deviations = 0	33.54843	1	< 0.00
		All Period Deviations = 0	0.438796	4	0.98	All Period Deviations = 0	0.561052	4	0.97	All Period Deviations = 0	0.029307	4	1.00
		All Cohort Deviations = 0	1.136384	6	0.98	All Cohort Deviations = 0	1.324457	6	0.97	All Cohort Deviations = 0	0.019542	6	1.00
		All Period RR = 1	42.07778	5	< 0.00	All Period RR = 1	27.39471	5	< 0.00	All Period RR = 1	0.171725	5	1.00
		All Cohort RR = 1	42.24455	7	< 0.00	All Cohort RR = 1	24.08882	7	< 0.00	All Cohort RR = 1	0.310841	7	1.00
		All Local Drifts = Net Drift	0.508999	3	0.92	All Local Drifts = Net Drift	0.921402	3	0.82	All Local Drifts = Net Drift	0.011107	3	1.00
	Male	Net Drift = 0	21.94055	1	< 0.00	Net Drift = 0	10.3963	1	< 0.00	Net Drift = 0	0.024846	1	0.87
		All Age Deviations = 0	50.2662	1	< 0.00	All Age Deviations = 0	226.0662	1	< 0.00	All Age Deviations = 0	22.41841	1	< 0.00
		All Period Deviations = 0	0.320766	4	0.99	All Period Deviations = 0	0.448194	4	0.98	All Period Deviations = 0	0.01588	4	1.00
		All Cohort Deviations = 0	0.455645	6	1.00	All Cohort Deviations = 0	0.397217	6	1.00	All Cohort Deviations = 0	0.058288	6	1.00
		All Period RR = 1	22.67627	5	< 0.00	All Period RR = 1	12.71023	5	0.03	All Period RR = 1	0.035142	5	1.00
		All Cohort RR = 1	22.4619	7	< 0.00	All Cohort RR = 1	11.29866	7	0.13	All Cohort RR = 1	0.237376	7	1.00
		All Local Drifts = Net Drift	0.142376	3	0.99	All Local Drifts = Net Drift	0.201572	3	0.98	All Local Drifts = Net Drift	0.035303	3	1.00
	Female	Net Drift = 0	20.70796	1	< 0.00	Net Drift = 0	13.125	1	< 0.00	Net Drift = 0	0.074596	1	0.78
		All Age Deviations = 0	30.72395	1	< 0.00	All Age Deviations = 0	207.1687	1	< 0.00	All Age Deviations = 0	10.48868	1	< 0.00
		All Period Deviations = 0	0.43249	4	0.98	All Period Deviations = 0	0.199057	4	1.00	All Period Deviations = 0	0.036822	4	1.00
		All Cohort Deviations = 0	0.429987	6	1.00	All Cohort Deviations = 0	1.124484	6	0.98	All Cohort Deviations = 0	0.151025	6	1.00
		All Period RR = 1	22.09302	5	< 0.00	All Period RR = 1	15.40072	5	0.01	All Period RR = 1	0.121098	5	1.00
		All Cohort RR = 1	21.39341	7	< 0.00	All Cohort RR = 1	13.41666	7	0.06	All Cohort RR = 1	0.152006	7	1.00
		All Local Drifts = Net Drift	0.212707	3	0.98	All Local Drifts = Net Drift	0.963477	3	0.81	All Local Drifts = Net Drift	0.14634	3	0.99
Sudan	Both	Net Drift = 0	140.0222	1	< 0.00	Net Drift = 0	55.37771	1	< 0.00	Net Drift = 0	0.206242	1	0.65
		All Age Deviations = 0	573.2139	1	< 0.00	All Age Deviations = 0	2153.265	1	< 0.00	All Age Deviations = 0	37.03369	1	< 0.00
		All Period Deviations = 0	12.64801	4	0.01	All Period Deviations = 0	4.761215	4	0.31	All Period Deviations = 0	0.025367	4	1.00

		All Cohort Deviations = 0	16.11745	6	0.01	All Cohort Deviations = 0	27.1899	6	< 0.00	All Cohort Deviations = 0	0.03663	6	1.00
		All Period RR = 1	159.1067	5	< 0.00	All Period RR = 1	67.82483	5	< 0.00	All Period RR = 1	0.354551	5	1.00
		All Cohort RR = 1	165.328	7	< 0.00	All Cohort RR = 1	68.26678	7	< 0.00	All Cohort RR = 1	0.836884	7	1.00
		All Local Drifts = Net Drift	12.88595	3	< 0.00	All Local Drifts = Net Drift	23.02787	3	< 0.00	All Local Drifts = Net Drift	0.005666	3	1.00
	Male	Net Drift = 0	12.00072	1	< 0.00	Net Drift = 0	12.12713	1	< 0.00	Net Drift = 0	0.067964	1	0.79
		All Age Deviations = 0	357.7578	1	< 0.00	All Age Deviations = 0	1212.775	1	< 0.00	All Age Deviations = 0	15.44149	1	< 0.00
		All Period Deviations = 0	4.641228	4	0.33	All Period Deviations = 0	2.683655	4	0.61	All Period Deviations = 0	0.122761	4	1.00
		All Cohort Deviations = 0	12.68543	6	0.05	All Cohort Deviations = 0	16.1678	6	0.01	All Cohort Deviations = 0	0.113834	6	1.00
		All Period RR = 1	17.38116	5	< 0.00	All Period RR = 1	18.94769	5	< 0.00	All Period RR = 1	0.319009	5	1.00
		All Cohort RR = 1	24.66194	7	< 0.00	All Cohort RR = 1	23.31944	7	< 0.00	All Cohort RR = 1	0.801036	7	1.00
		All Local Drifts = Net Drift	9.021801	3	< 0.03	All Local Drifts = Net Drift	13.53741	3	< 0.00	All Local Drifts = Net Drift	0.052083	3	1.00
	Female	Net Drift = 0	190.3453	1	< 0.00	Net Drift = 0	38.76843	1	< 0.00	Net Drift = 0	0.056082	1	0.81
		All Age Deviations = 0	218.1838	1	< 0.00	All Age Deviations = 0	939.8486	1	< 0.00	All Age Deviations = 0	21.60665	1	< 0.00
		All Period Deviations = 0	4.183222	4	0.38	All Period Deviations = 0	20.34762	4	< 0.00	All Period Deviations = 0	0.122162	4	1.00
		All Cohort Deviations = 0	13.07943	6	0.04	All Cohort Deviations = 0	26.94106	6	< 0.00	All Cohort Deviations = 0	0.017599	6	1.00
		All Period RR = 1	203.2956	5	< 0.00	All Period RR = 1	56.58083	5	< 0.00	All Period RR = 1	0.263583	5	1.00
		All Cohort RR = 1	213.7521	7	< 0.00	All Cohort RR = 1	55.24805	7	< 0.00	All Cohort RR = 1	0.157456	7	1.00
		All Local Drifts = Net Drift	12.53772	3	0.01	All Local Drifts = Net Drift	25.55741	3	< 0.00	All Local Drifts = Net Drift	0.00741	3	1.00
Togo	Both	Net Drift = 0	86.85306	1	< 0.00	Net Drift = 0	64.58563	1	< 0.00	Net Drift = 0	0.003233	1	0.95
		All Age Deviations = 0	65.62081	1	< 0.00	All Age Deviations = 0	307.5326	1	< 0.00	All Age Deviations = 0	9.765079	1	< 0.00
		All Period Deviations = 0	0.566278	4	0.97	All Period Deviations = 0	0.115174	4	1.00	All Period Deviations = 0	0.163036	4	1.00
		All Cohort Deviations = 0	3.356759	6	0.76	All Cohort Deviations = 0	0.697343	6	0.99	All Cohort Deviations = 0	0.090894	6	1.00
		All Period RR = 1	89.9672	5	< 0.00	All Period RR = 1	73.56639	5	< 0.00	All Period RR = 1	0.168304	5	1.00
		All Cohort RR = 1	98.93046	7	< 0.00	All Cohort RR = 1	75.83512	7	< 0.00	All Cohort RR = 1	0.090894	7	1.00

		All Local Drifts = Net Drift	3.319953	3	0.34	All Local Drifts = Net Drift	0.514568	3	0.92	All Local Drifts = Net Drift	0.025912	3	1.00
	Male	Net Drift = 0	17.93156	1	< 0.00	Net Drift = 0	16.62025	1	< 0.00	Net Drift = 0	0.023633	1	0.88
		All Age Deviations = 0	32.104	1	< 0.00	All Age Deviations = 0	137.5602	1	< 0.00	All Age Deviations = 0	3.737437	1	0.05
		All Period Deviations = 0	0.261497	4	0.99	All Period Deviations = 0	0.045915	4	1.00	All Period Deviations = 0	0.145635	4	1.00
		All Cohort Deviations = 0	0.558702	6	1.00	All Cohort Deviations = 0	0.384105	6	1.00	All Cohort Deviations = 0	0.14026	6	1.00
		All Period RR = 1	18.85483	5	< 0.00	All Period RR = 1	19.04147	5	< 0.00	All Period RR = 1	0.250215	5	1.00
		All Cohort RR = 1	19.43785	7	0.01	All Cohort RR = 1	20.15698	7	0.01	All Cohort RR = 1	0.252959	7	1.00
		All Local Drifts = Net Drift	0.494234	3	0.92	All Local Drifts = Net Drift	0.173086	3	0.98	All Local Drifts = Net Drift	0.07542	3	0.99
	Female	Net Drift = 0	82.2007	1	< 0.00	Net Drift = 0	53.26192	1	< 0.00	Net Drift = 0	0.130992	1	0.72
		All Age Deviations = 0	32.54799	1	< 0.00	All Age Deviations = 0	169.0597	1	< 0.00	All Age Deviations = 0	4.371611	1	0.04
		All Period Deviations = 0	0.337922	4	0.99	All Period Deviations = 0	0.216596	4	0.99	All Period Deviations = 0	0.072916	4	1.00
		All Cohort Deviations = 0	3.246207	6	0.78	All Cohort Deviations = 0	0.803408	6	0.99	All Cohort Deviations = 0	0.270882	6	1.00
		All Period RR = 1	84.21398	5	< 0.00	All Period RR = 1	60.18326	5	< 0.00	All Period RR = 1	0.162974	5	1.00
		All Cohort RR = 1	94.84788	7	< 0.00	All Cohort RR = 1	61.36556	7	< 0.00	All Cohort RR = 1	0.270883	7	1.00
		All Local Drifts = Net Drift	3.205638	3	0.36	All Local Drifts = Net Drift	0.797362	3	0.85	All Local Drifts = Net Drift	0.247279	3	0.97
Tunisia	Both	Net Drift = 0	56.24209	1	< 0.00	Net Drift = 0	86.65099	1	< 0.00	Net Drift = 0	0.009799	1	0.92
		All Age Deviations = 0	139.1954	1	< 0.00	All Age Deviations = 0	520.9551	1	< 0.00	All Age Deviations = 0	7.37312	1	0.01
		All Period Deviations = 0	0.295268	4	0.99	All Period Deviations = 0	1.624356	4	0.80	All Period Deviations = 0	0.068945	4	1.00
		All Cohort Deviations = 0	5.142185	6	0.53	All Cohort Deviations = 0	3.820182	6	0.70	All Cohort Deviations = 0	0.047181	6	1.00
		All Period RR = 1	56.99372	5	< 0.00	All Period RR = 1	87.1852	5	< 0.00	All Period RR = 1	0.069707	5	1.00
		All Cohort RR = 1	61.5316	7	< 0.00	All Cohort RR = 1	93.27044	7	< 0.00	All Cohort RR = 1	0.054935	7	1.00
		All Local Drifts = Net Drift	4.656662	3	0.20	All Local Drifts = Net Drift	3.647044	3	0.30	All Local Drifts = Net Drift	0.031845	3	1.00
	Male	Net Drift = 0	12.70962	1	< 0.00	Net Drift = 0	34.58672	1	< 0.00	Net Drift = 0	0.006165	1	0.94
		All Age Deviations = 0	86.37599	1	< 0.00	All Age Deviations = 0	351.215	1	< 0.00	All Age Deviations = 0	4.027137	1	0.04

		All Period Deviations = 0	0.302006	4	0.99	All Period Deviations = 0	1.592411	4	0.81	All Period Deviations = 0	0.054961	4	1.00
		All Cohort Deviations = 0	1.09501	6	0.98	All Cohort Deviations = 0	3.563123	6	0.74	All Cohort Deviations = 0	0.054656	6	1.00
		All Period RR = 1	13.30569	5	0.02	All Period RR = 1	34.98599	5	< 0.00	All Period RR = 1	0.056527	5	1.00
		All Cohort RR = 1	14.09739	7	0.05	All Cohort RR = 1	36.99439	7	< 0.00	All Cohort RR = 1	0.097093	7	1.00
		All Local Drifts = Net Drift	0.836792	3	0.84	All Local Drifts = Net Drift	3.459294	3	0.33	All Local Drifts = Net Drift	0.013553	3	1.00
	Female	Net Drift = 0	57.85574	1	< 0.00	Net Drift = 0	80.29765	1	< 0.00	Net Drift = 0	0.001228	1	0.97
		All Age Deviations = 0	52.29132	1	< 0.00	All Age Deviations = 0	259.6123	1	< 0.00	All Age Deviations = 0	4.288334	1	0.04
		All Period Deviations = 0	0.068279	4	1.00	All Period Deviations = 0	0.375755	4	0.98	All Period Deviations = 0	0.045903	4	1.00
		All Cohort Deviations = 0	6.552117	6	0.36	All Cohort Deviations = 0	0.638121	6	1.00	All Cohort Deviations = 0	0.031758	6	1.00
		All Period RR = 1	58.0935	5	< 0.00	All Period RR = 1	81.62088	5	< 0.00	All Period RR = 1	0.048465	5	1.00
		All Cohort RR = 1	65.25628	7	< 0.00	All Cohort RR = 1	89.29717	7	< 0.00	All Cohort RR = 1	0.033101	7	1.00
		All Local Drifts = Net Drift	6.271274	3	0.10	All Local Drifts = Net Drift	0.572649	3	0.90	All Local Drifts = Net Drift	0.002855	3	1.00
Uganda	Both	Net Drift = 0	632.2096	1	< 0.00	Net Drift = 0	34.57888	1	< 0.00	Net Drift = 0	0.029194	1	0.86
		All Age Deviations = 0	295.1375	1	< 0.00	All Age Deviations = 0	594.3172	1	< 0.00	All Age Deviations = 0	54.42097	1	< 0.00
		All Period Deviations = 0	0.702784	4	0.95	All Period Deviations = 0	50.75101	4	< 0.00	All Period Deviations = 0	1.374855	4	0.85
		All Cohort Deviations = 0	100.7901	6	< 0.00	All Cohort Deviations = 0	77.76643	6	< 0.00	All Cohort Deviations = 0	2.990922	6	0.81
		All Period RR = 1	654.9922	5	< 0.00	All Period RR = 1	92.04165	5	< 0.00	All Period RR = 1	2.07891	5	0.84
		All Cohort RR = 1	729.5808	7	< 0.00	All Cohort RR = 1	95.53746	7	< 0.00	All Cohort RR = 1	7.702472	7	0.36
		All Local Drifts = Net Drift	92.81575	3	< 0.00	All Local Drifts = Net Drift	63.44043	3	< 0.00	All Local Drifts = Net Drift	2.878583	3	0.41
	Male	Net Drift = 0	274.1237	1	< 0.00	Net Drift = 0	61.48343	1	< 0.00	Net Drift = 0	0.005617	1	0.94
		All Age Deviations = 0	158.0604	1	< 0.00	All Age Deviations = 0	748.1058	1	< 0.00	All Age Deviations = 0	26.8182	1	< 0.00
		All Period Deviations = 0	1.034967	4	0.90	All Period Deviations = 0	54.20175	4	< 0.00	All Period Deviations = 0	1.554027	4	0.82
		All Cohort Deviations = 0	43.53196	6	< 0.00	All Cohort Deviations = 0	90.87511	6	< 0.00	All Cohort Deviations = 0	2.690766	6	0.85
		All Period RR = 1	283.0427	5	< 0.00	All Period RR = 1	115.5144	5	< 0.00	All Period RR = 1	2.135365	5	0.83

		All Cohort RR = 1	315.806	7	< 0.00	All Cohort RR = 1	128.7163	7	< 0.00	All Cohort RR = 1	7.230186	7	0.41
		All Local Drifts = Net Drift	40.02924	3	< 0.00	All Local Drifts = Net Drift	77.84019	3	< 0.00	All Local Drifts = Net Drift	2.561621	3	0.46
	Female	Net Drift = 0	353.4759	1	< 0.00	Net Drift = 0	19.20758	1	< 0.00	Net Drift = 0	0.003779	1	0.95
		All Age Deviations = 0	132.5565	1	< 0.00	All Age Deviations = 0	477.4541	1	< 0.00	All Age Deviations = 0	27.20447	1	< 0.00
		All Period Deviations = 0	0.310942	4	0.99	All Period Deviations = 0	54.51093	4	< 0.00	All Period Deviations = 0	0.210665	4	0.99
		All Cohort Deviations = 0	59.55677	6	< 0.00	All Cohort Deviations = 0	67.08495	6	< 0.00	All Cohort Deviations = 0	0.663536	6	1.00
		All Period RR = 1	366.1735	5	< 0.00	All Period RR = 1	83.7735	5	< 0.00	All Period RR = 1	0.270587	5	1.00
		All Cohort RR = 1	409.773	7	< 0.00	All Cohort RR = 1	74.73493	7	< 0.00	All Cohort RR = 1	1.174052	7	0.99
		All Local Drifts = Net Drift	54.83463	3	< 0.00	All Local Drifts = Net Drift	51.83171	3	< 0.00	All Local Drifts = Net Drift	0.649022	3	0.89
United Republic of Tanzania	Both	Net Drift = 0	673.6606	1	< 0.00	Net Drift = 0	439.3733	1	< 0.00	Net Drift = 0	0.011861	1	0.91
		All Age Deviations = 0	186.2887	1	< 0.00	All Age Deviations = 0	2116.377	1	< 0.00	All Age Deviations = 0	243.1829	1	< 0.00
		All Period Deviations = 0	0.72561	4	0.95	All Period Deviations = 0	1.227624	4	0.87	All Period Deviations = 0	0.393929	4	0.98
		All Cohort Deviations = 0	32.26153	6	< 0.00	All Cohort Deviations = 0	19.76977	6	< 0.00	All Cohort Deviations = 0	0.623965	6	1.00
		All Period RR = 1	692.6503	5	< 0.00	All Period RR = 1	500.5861	5	< 0.00	All Period RR = 1	0.403432	5	1.00
		All Cohort RR = 1	769.089	7	< 0.00	All Cohort RR = 1	524.4606	7	< 0.00	All Cohort RR = 1	0.732186	7	1.00
		All Local Drifts = Net Drift	31.65247	3	< 0.00	All Local Drifts = Net Drift	19.23779	3	< 0.00	All Local Drifts = Net Drift	0.473551	3	0.92
	Male	Net Drift = 0	309.7502	1	< 0.00	Net Drift = 0	150.6828	1	< 0.00	Net Drift = 0	0.587776	1	0.44
		All Age Deviations = 0	105.3457	1	< 0.00	All Age Deviations = 0	802.5399	1	< 0.00	All Age Deviations = 0	141.4977	1	< 0.00
		All Period Deviations = 0	1.434849	4	0.84	All Period Deviations = 0	1.251304	4	0.87	All Period Deviations = 0	0.467764	4	0.98
		All Cohort Deviations = 0	15.30315	6	0.02	All Cohort Deviations = 0	10.87649	6	0.09	All Cohort Deviations = 0	0.762384	6	0.99
		All Period RR = 1	314.6433	5	< 0.00	All Period RR = 1	176.5063	5	< 0.00	All Period RR = 1	1.033587	5	0.96
		All Cohort RR = 1	354.9618	7	< 0.00	All Cohort RR = 1	188.9794	7	< 0.00	All Cohort RR = 1	2.415236	7	0.93
		All Local Drifts = Net Drift	15.01394	3	< 0.00	All Local Drifts = Net Drift	10.29122	3	0.02	All Local Drifts = Net Drift	0.598729	3	0.90
	Female	Net Drift = 0	369.6175	1	< 0.00	Net Drift = 0	297.6262	1	< 0.00	Net Drift = 0	1.647394	1	0.20

		All Age Deviations = 0	75.59592	1	< 0.00	All Age Deviations = 0	1337.015	1	< 0.00	All Age Deviations = 0	100.3292	1	< 0.00
		All Period Deviations = 0	0.162725	4	1.00	All Period Deviations = 0	1.862957	4	0.76	All Period Deviations = 0	0.339675	4	0.99
		All Cohort Deviations = 0	17.49783	6	0.01	All Cohort Deviations = 0	11.00685	6	0.09	All Cohort Deviations = 0	0.116024	6	1.00
		All Period RR = 1	385.7362	5	< 0.00	All Period RR = 1	334.1451	5	< 0.00	All Period RR = 1	1.690799	5	0.89
		All Cohort RR = 1	421.2875	7	< 0.00	All Cohort RR = 1	345.0428	7	< 0.00	All Cohort RR = 1	4.838414	7	0.68
		All Local Drifts = Net Drift	17.00512	3	< 0.00	All Local Drifts = Net Drift	10.84283	3	0.01	All Local Drifts = Net Drift	0.109579	3	0.99
Zambia	Both	Net Drift = 0	201.8652	1	< 0.00	Net Drift = 0	58.9261	1	< 0.00	Net Drift = 0	0.232951	1	0.63
		All Age Deviations = 0	196.6398	1	< 0.00	All Age Deviations = 0	1156.026	1	< 0.00	All Age Deviations = 0	69.11605	1	< 0.00
		All Period Deviations = 0	1.558565	4	0.82	All Period Deviations = 0	13.87816	4	0.01	All Period Deviations = 0	0.214444	4	0.99
		All Cohort Deviations = 0	13.4465	6	0.04	All Cohort Deviations = 0	5.389557	6	0.49	All Cohort Deviations = 0	0.326942	6	1.00
		All Period RR = 1	211.7231	5	< 0.00	All Period RR = 1	65.86164	5	< 0.00	All Period RR = 1	0.325506	5	1.00
		All Cohort RR = 1	226.7407	7	< 0.00	All Cohort RR = 1	78.56813	7	< 0.00	All Cohort RR = 1	0.691474	7	1.00
		All Local Drifts = Net Drift	10.04182	3	0.02	All Local Drifts = Net Drift	2.18789	3	0.53	All Local Drifts = Net Drift	0.320711	3	0.96
	Male	Net Drift = 0	61.27575	1	< 0.00	Net Drift = 0	10.88964	1	< 0.00	Net Drift = 0	0.030132	1	0.86
		All Age Deviations = 0	92.56967	1	< 0.00	All Age Deviations = 0	515.674	1	< 0.00	All Age Deviations = 0	39.27262	1	< 0.00
		All Period Deviations = 0	1.151851	4	0.89	All Period Deviations = 0	5.726557	4	0.22	All Period Deviations = 0	0.057474	4	1.00
		All Cohort Deviations = 0	6.618198	6	0.36	All Cohort Deviations = 0	10.09284	6	0.12	All Cohort Deviations = 0	0.042233	6	1.00
		All Period RR = 1	63.68974	5	< 0.00	All Period RR = 1	15.14896	5	0.01	All Period RR = 1	0.0756	5	1.00
		All Cohort RR = 1	71.07783	7	< 0.00	All Cohort RR = 1	28.79801	7	< 0.00	All Cohort RR = 1	0.072325	7	1.00
		All Local Drifts = Net Drift	4.783957	3	0.19	All Local Drifts = Net Drift	6.406384	3	0.09	All Local Drifts = Net Drift	0.027291	3	1.00
	Female	Net Drift = 0	147.6209	1	< 0.00	Net Drift = 0	54.79457	1	< 0.00	Net Drift = 0	0.829909	1	0.36
		All Age Deviations = 0	104.0363	1	< 0.00	All Age Deviations = 0	642.2522	1	< 0.00	All Age Deviations = 0	31.55203	1	< 0.00
		All Period Deviations = 0	0.796832	4	0.94	All Period Deviations = 0	9.136614	4	0.06	All Period Deviations = 0	0.462317	4	0.98
		All Cohort Deviations = 0	7.554583	6	0.27	All Cohort Deviations = 0	1.267927	6	0.97	All Cohort Deviations = 0	0.606366	6	1.00

		All Period RR = 1	156.4374	5	< 0.00	All Period RR = 1	57.7754	5	< 0.00	All Period RR = 1	0.934446	5	0.97
		All Cohort RR = 1	164.1983	7	0.00	All Cohort RR = 1	60.43757	7	< 0.00	All Cohort RR = 1	1.485048	7	0.98
		All Local Drifts = Net Drift	5.893103	3	0.12	All Local Drifts = Net Drift	0.129835	3	0.99	All Local Drifts = Net Drift	0.561221	3	0.91
Zimbabwe	Both	Net Drift = 0	9.194383	1	< 0.00	Net Drift = 0	13.47174	1	< 0.00	Net Drift = 0	0.005531	1	0.94
		All Age Deviations = 0	154.7228	1	< 0.00	All Age Deviations = 0	584.7442	1	< 0.00	All Age Deviations = 0	26.90875	1	< 0.00
		All Period Deviations = 0	0.244488	4	0.99	All Period Deviations = 0	10.56696	4	0.03	All Period Deviations = 0	0.028877	4	1.00
		All Cohort Deviations = 0	28.38293	6	< 0.00	All Cohort Deviations = 0	18.02495	6	0.01	All Cohort Deviations = 0	0.079685	6	1.00
		All Period RR = 1	9.578954	5	0.09	All Period RR = 1	30.53126	5	< 0.00	All Period RR = 1	0.029292	5	1.00
		All Cohort RR = 1	35.64309	7	< 0.00	All Cohort RR = 1	45.57502	7	< 0.00	All Cohort RR = 1	0.138209	7	1.00
		All Local Drifts = Net Drift	23.90063	3	< 0.00	All Local Drifts = Net Drift	13.98954	3	< 0.00	All Local Drifts = Net Drift	0.063851	3	1.00
	Male	Net Drift = 0	0.091263	1	0.76	Net Drift = 0	5.600784	1	0.02	Net Drift = 0	0.144839	1	0.70
		All Age Deviations = 0	76.71042	1	< 0.00	All Age Deviations = 0	254.6861	1	< 0.00	All Age Deviations = 0	11.76293	1	< 0.00
		All Period Deviations = 0	0.120225	4	1.00	All Period Deviations = 0	4.619378	4	0.33	All Period Deviations = 0	0.03845	4	1.00
		All Cohort Deviations = 0	17.17872	6	0.01	All Cohort Deviations = 0	10.53969	6	0.10	All Cohort Deviations = 0	0.526225	6	1.00
		All Period RR = 1	0.226257	5	1.00	All Period RR = 1	13.02747	5	0.02	All Period RR = 1	0.273155	5	1.00
		All Cohort RR = 1	17.1881	7	0.02	All Cohort RR = 1	23.55579	7	< 0.00	All Cohort RR = 1	0.534838	7	1.00
		All Local Drifts = Net Drift	14.2288	3	< 0.00	All Local Drifts = Net Drift	8.183105	3	0.04	All Local Drifts = Net Drift	0.514898	3	0.92
	Female	Net Drift = 0	19.21711	1	< 0.00	Net Drift = 0	7.883765	1	< 0.00	Net Drift = 0	0.025505	1	0.87
		All Age Deviations = 0	77.94985	1	< 0.00	All Age Deviations = 0	334.7476	1	< 0.00	All Age Deviations = 0	18.49579	1	< 0.00
		All Period Deviations = 0	0.641808	4	0.96	All Period Deviations = 0	6.225782	4	0.18	All Period Deviations = 0	0.059796	4	1.00
		All Cohort Deviations = 0	13.43652	6	0.04	All Cohort Deviations = 0	7.928268	6	0.24	All Cohort Deviations = 0	0.066	6	1.00
		All Period RR = 1	19.84006	5	< 0.00	All Period RR = 1	17.72652	5	< 0.00	All Period RR = 1	0.116179	5	1.00
		All Cohort RR = 1	29.81432	7	< 0.00	All Cohort RR = 1	22.49834	7	< 0.00	All Cohort RR = 1	0.098148	7	1.00
		All Local Drifts = Net Drift	11.86024	3	0.01	All Local Drifts = Net Drift	6.219107	3	0.10	All Local Drifts = Net Drift	0.060839	3	1.00

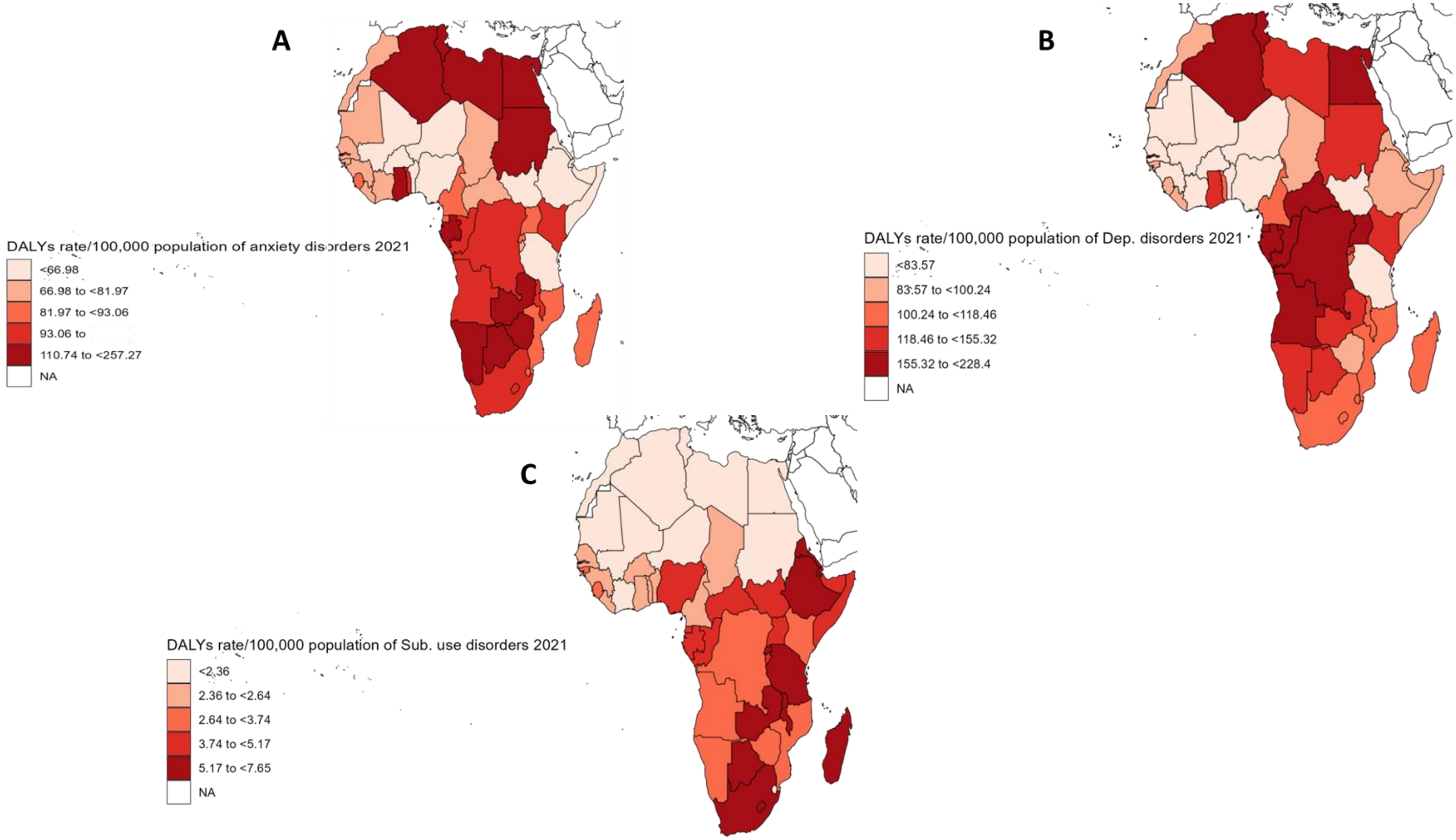

Figure S1. DALYs rate per 100, 000 population aged 10-24 years of anxiety (A), depression (B), and substance use disorder(C) attributable to childhood maltreatment in 2021 in 52 African countries.

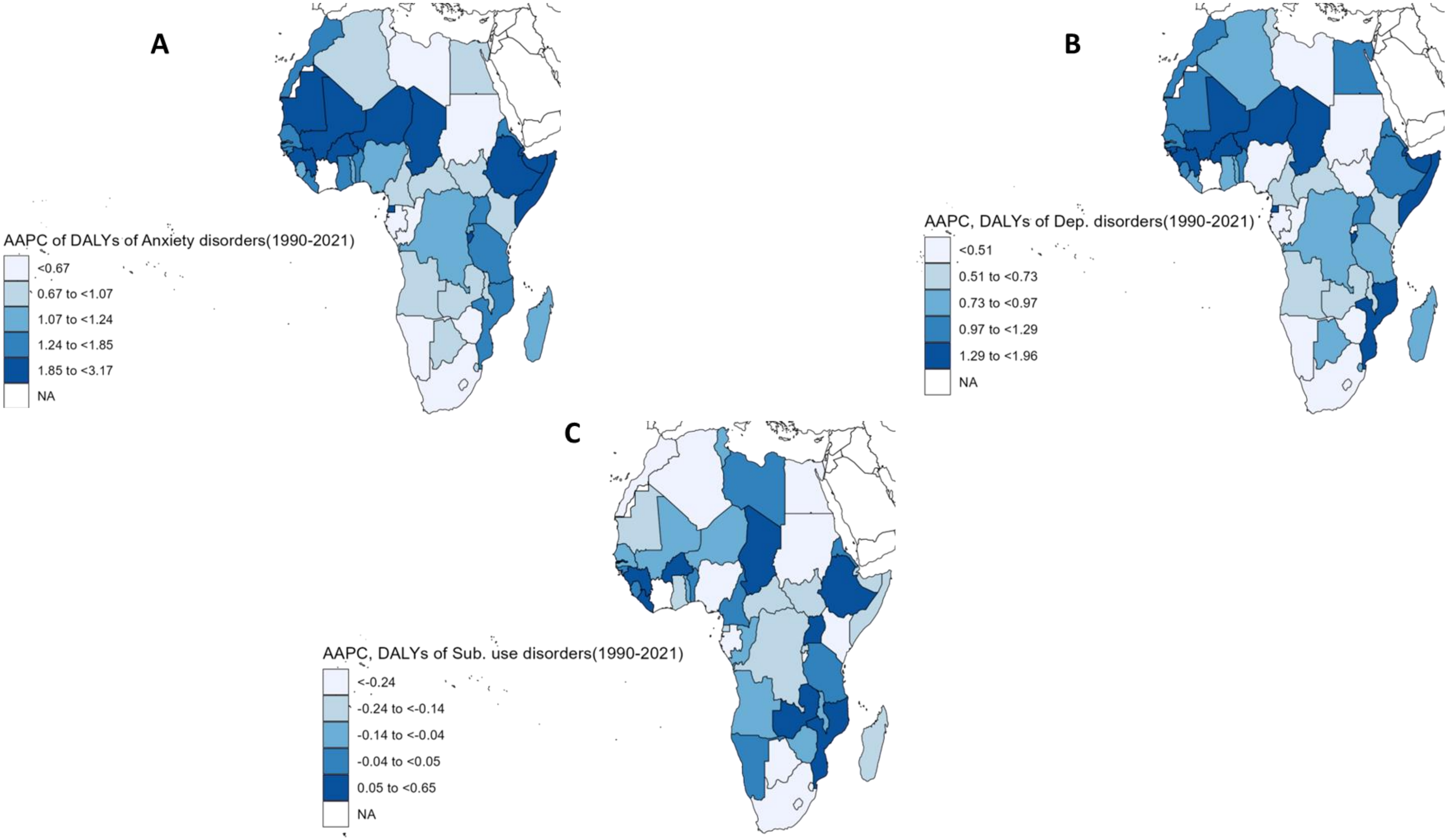

Figure S2. AAPC in DALY rate of anxiety (SA1), depression (SB1), and substance use disorder (SC1) attributed to childhood maltreatment in 10–24-year population in 52 African countries.

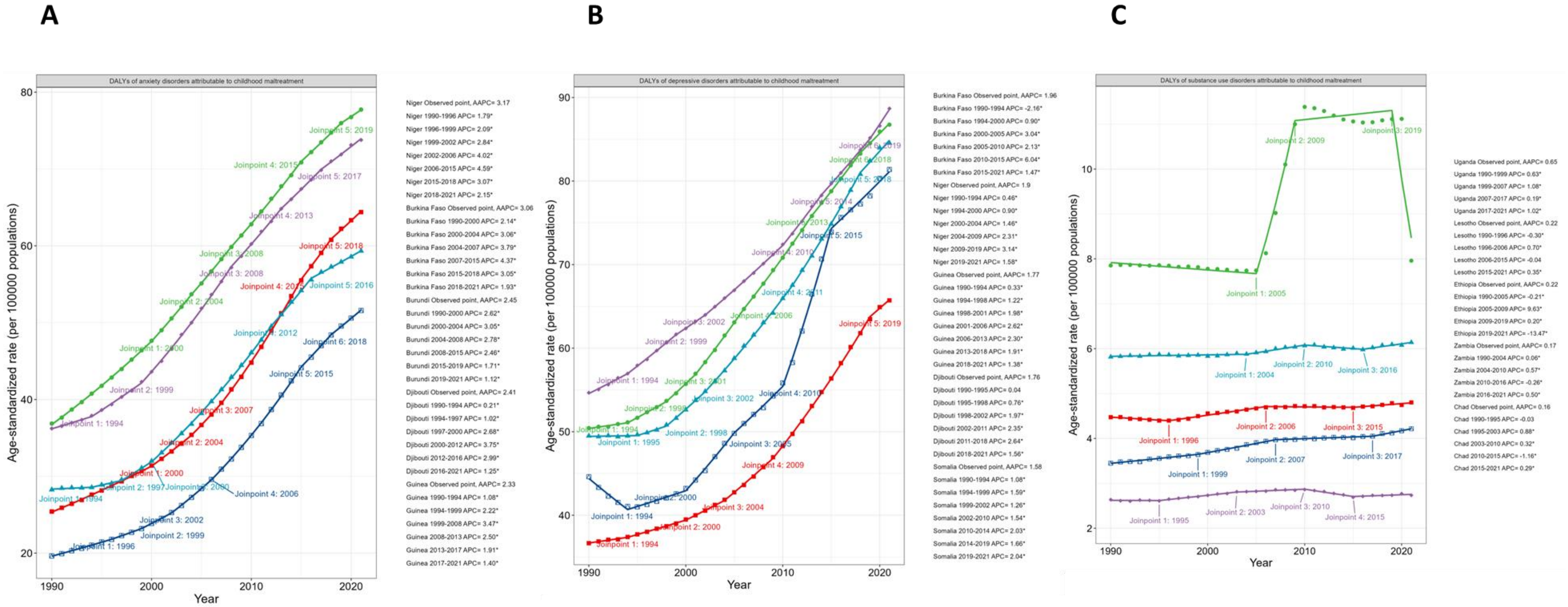

Figure S3. Joinpoint regression analysis of DALY rate of anxiety (A), depression (B), and substance use disorder (C) attributed to childhood maltreatment in the African countries with the top 5 highest recorded AAPC from 1990-2021.

The joinpoints indicate a turning point demarking significance. *Indicates p <0.05. AAPC- average annual percentage change, APC- annual percentage change.

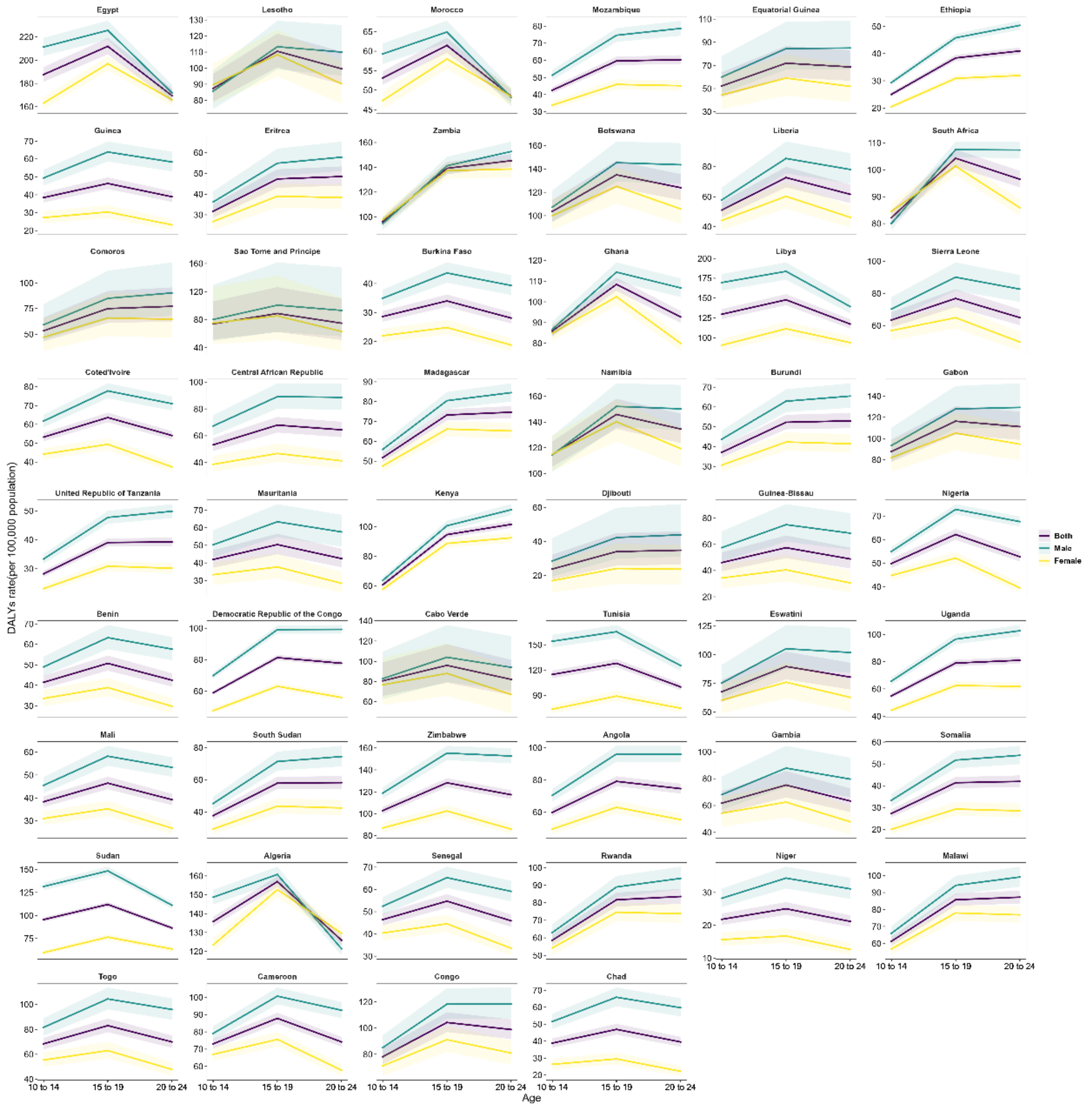

Figure S4. Age effects on anxiety disorders attributable to childhood maltreatment in 52 African countries.
The age effect on DALY of anxiety is depicted through the longitudinal rates specific to age, adjusted for variations across different birth cohorts, considering the period-specific deviations. The shaded areas denote the corresponding 95% CIs. DALY- Disability-adjusted life years.

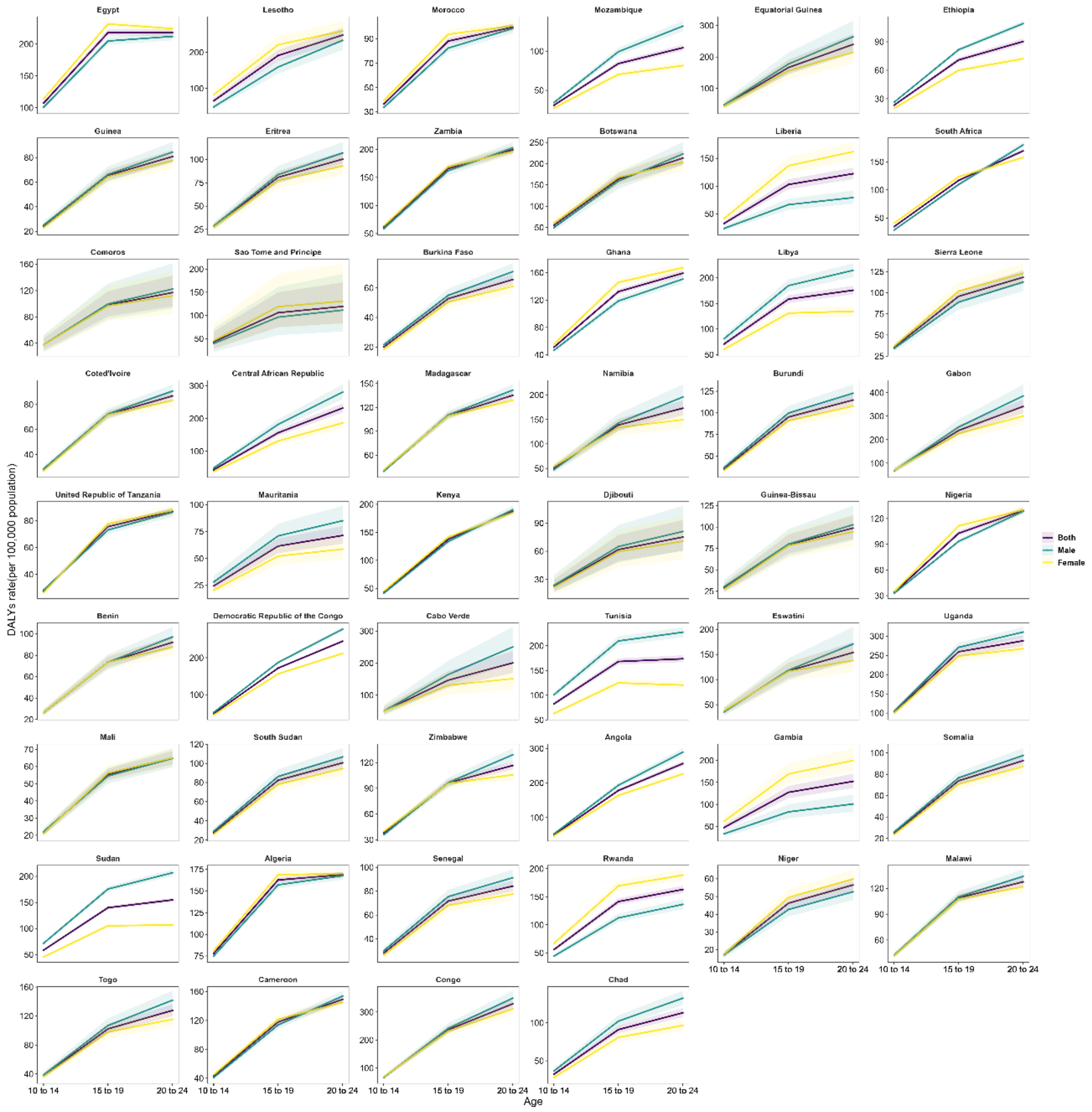

Figure S5. Age effects on depressive disorders attributable to childhood maltreatment in 52 African countries.

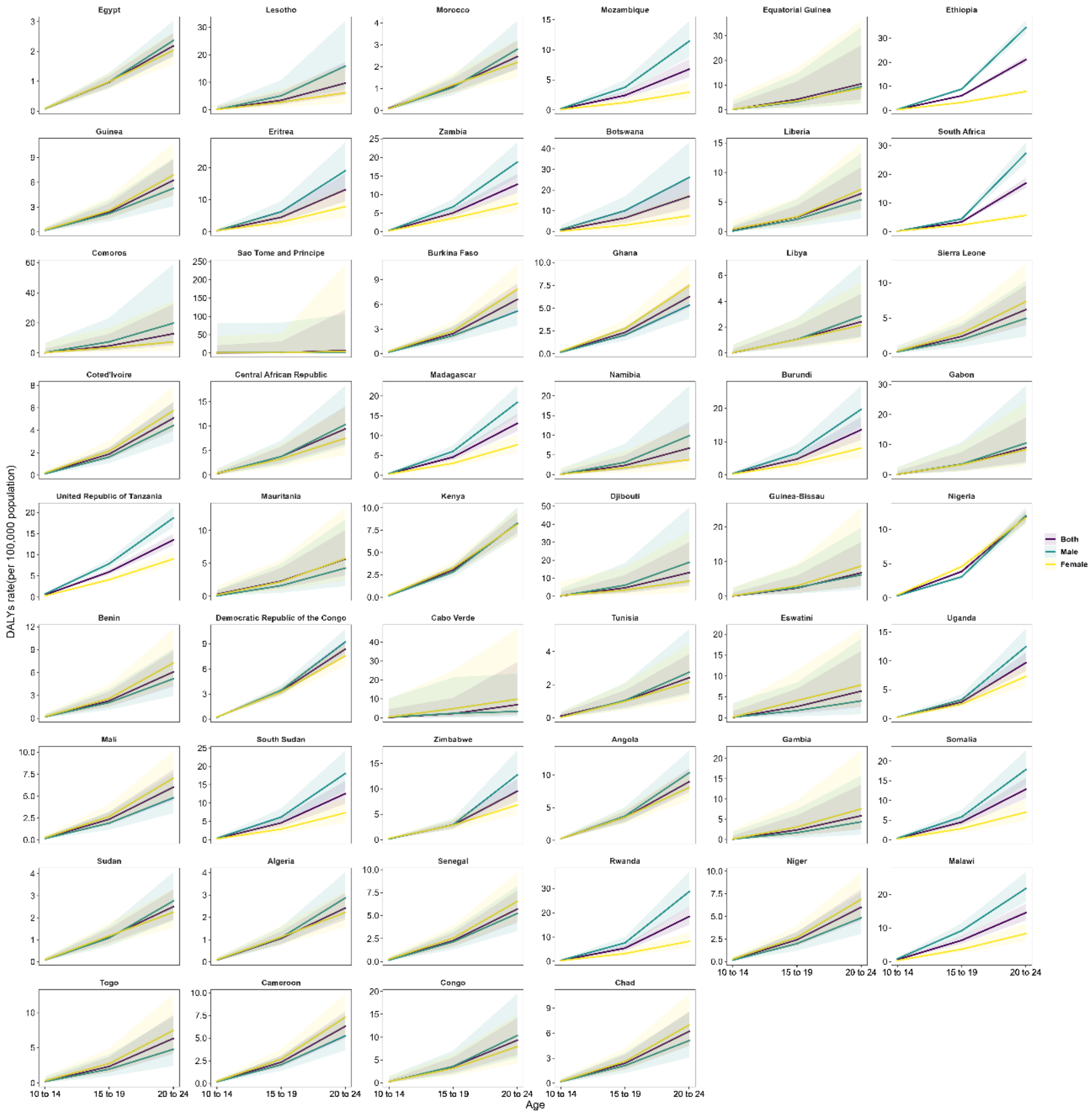

Figure S6. Age effects on substance use disorders attributable to childhood maltreatment in 52 African countries.

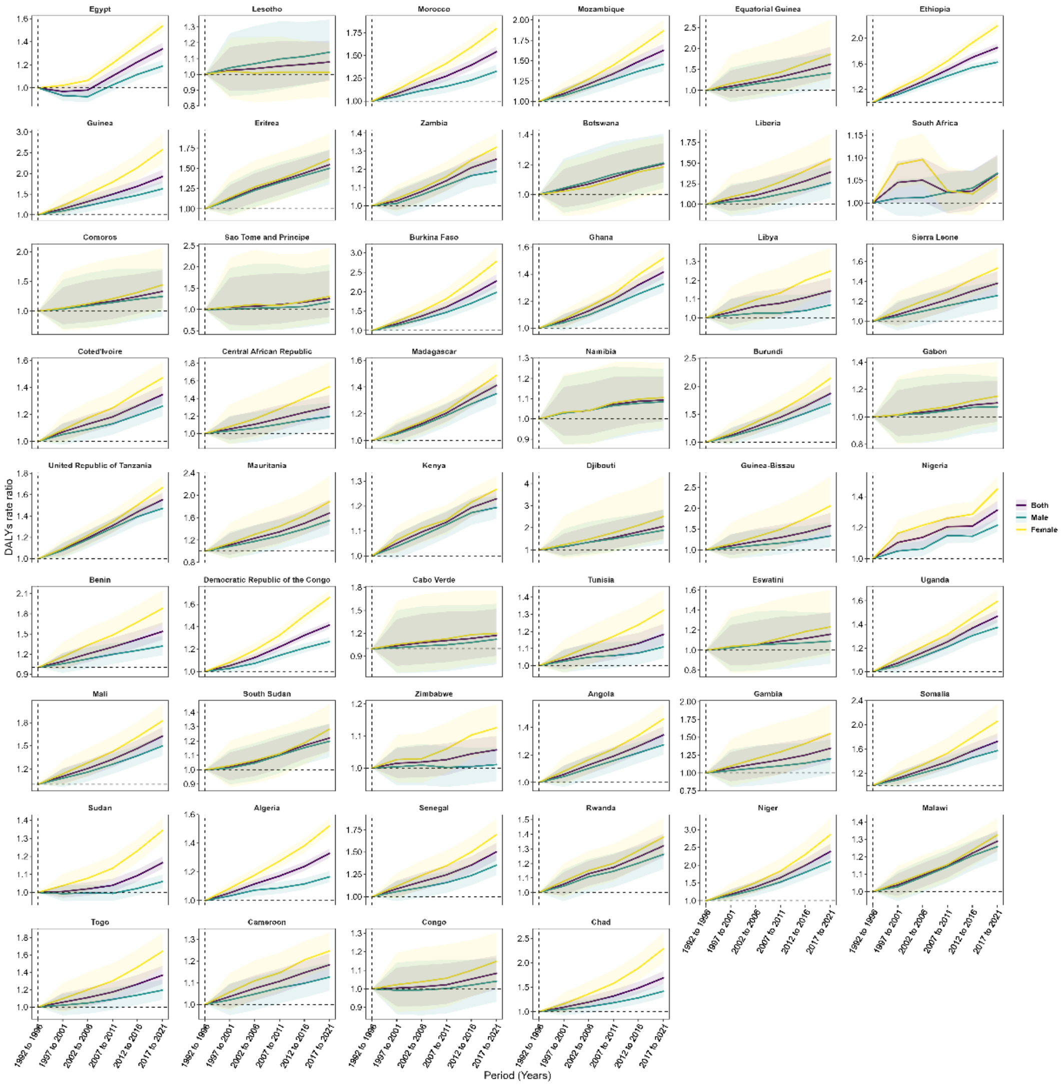

Figure S7. Period effects on anxiety disorders attributable to childhood maltreatment in 52 African countries.

Period effects are shown through the relative risk of DALYs of anxiety during different periods, calculated as the ratio of the age-specific rates from the period from 1992 - 1996 to 2017 - 2021, with the reference period set as 1992 - 1996. The shaded areas denote the corresponding 95% CIs. DALY- Disability-adjusted life years.

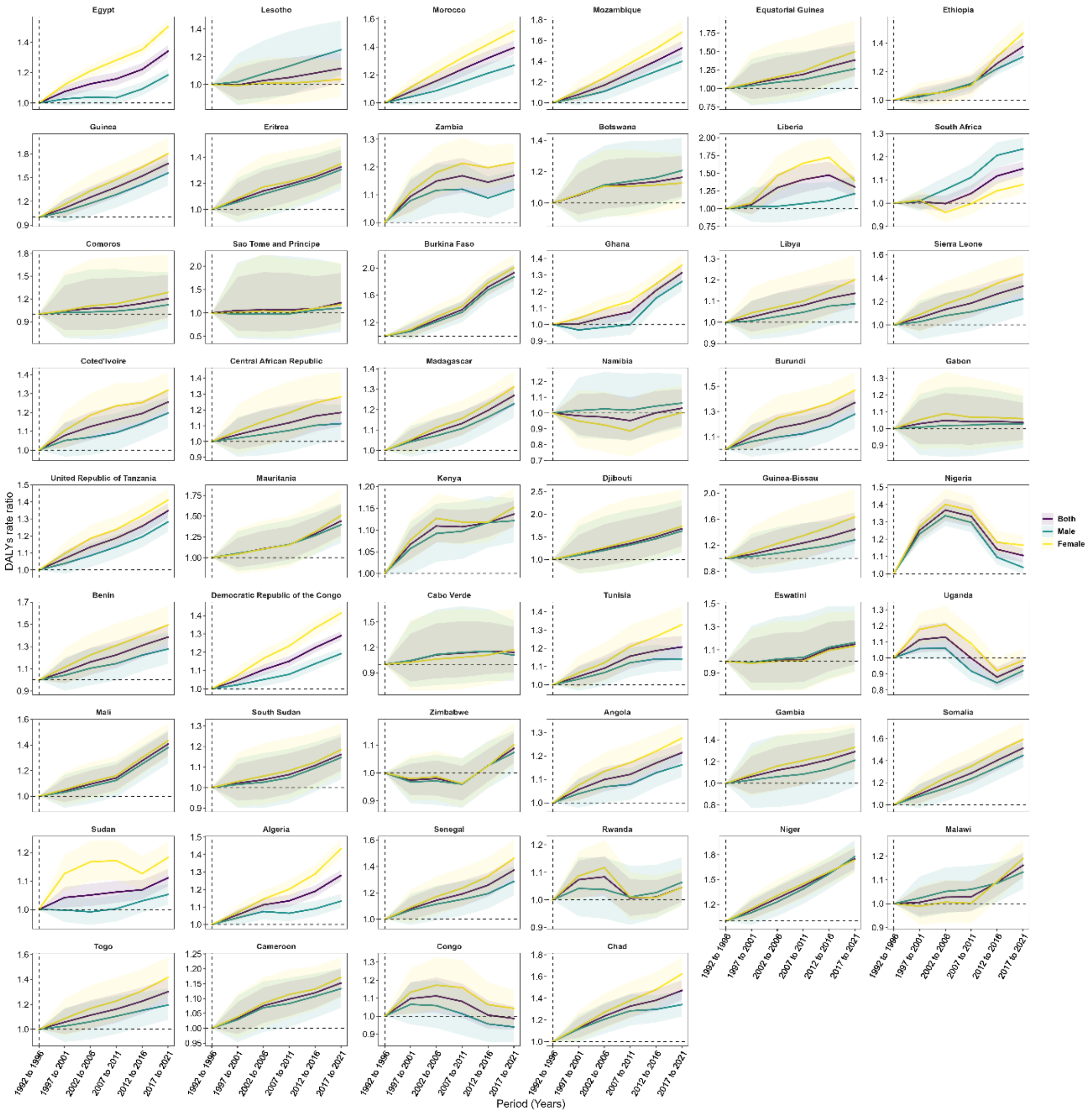

Figure S8. Period effects on depressive disorders attributable to childhood maltreatment in 52 African countries.

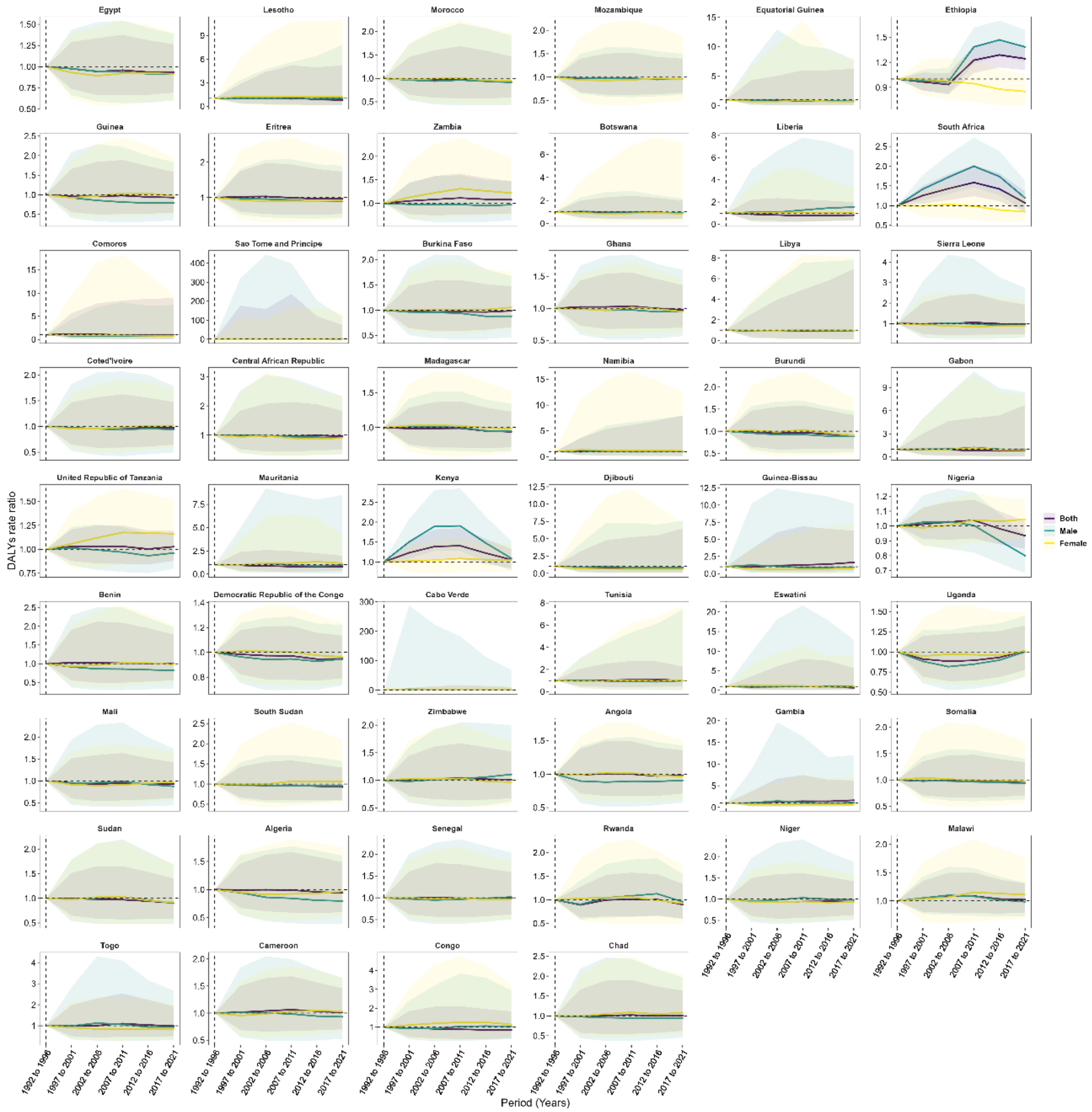

Figure S9. Period effects on substance use disorders attributable to childhood maltreatment in 52 African countries.

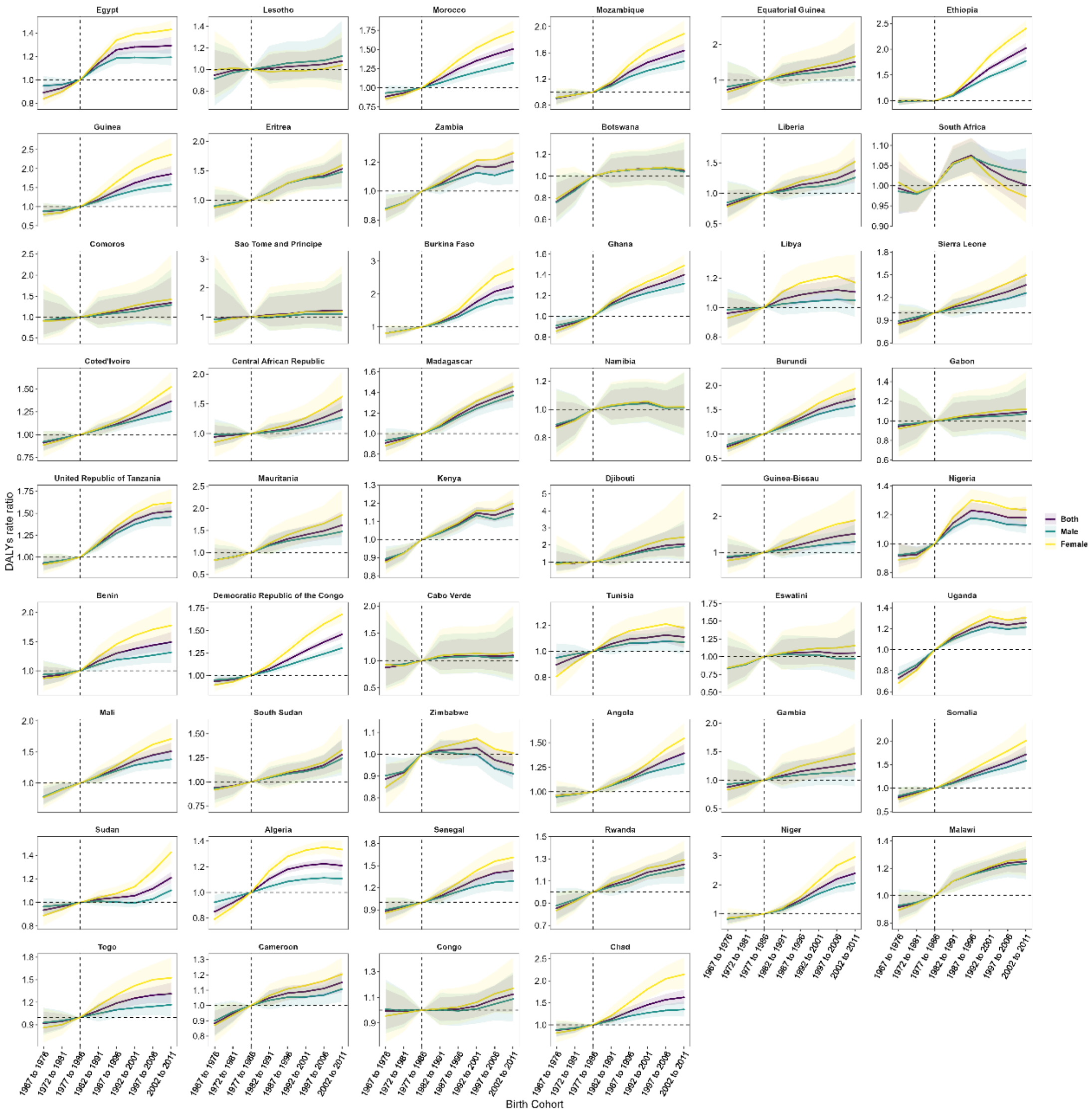

Figure S10 Cohort effects on anxiety disorders attributable to childhood maltreatment in 52 African countries.
Birth cohort effects are demonstrated by the cohort relative risk of DALYs and calculated as the ratio of age-specific rates from the 1967–1976 cohort to the 2002–2011 cohort, with the reference cohort set at 1977–1986. The shaded areas denote the corresponding 95% CIs. DALY- Disability-adjusted life years.

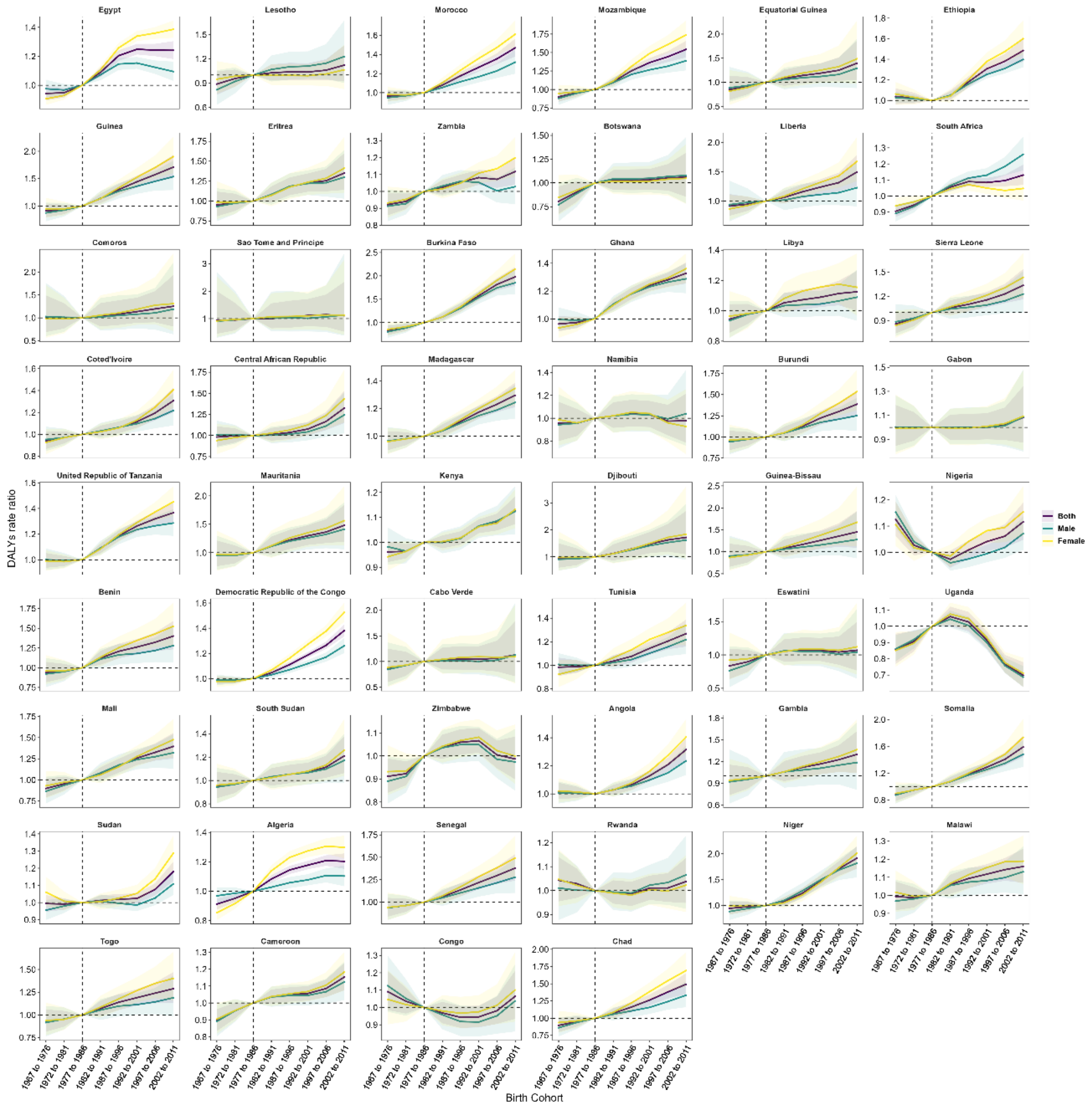

Figure S11 Cohort effects on depressive disorders attributable to childhood maltreatment in 52 African countries.

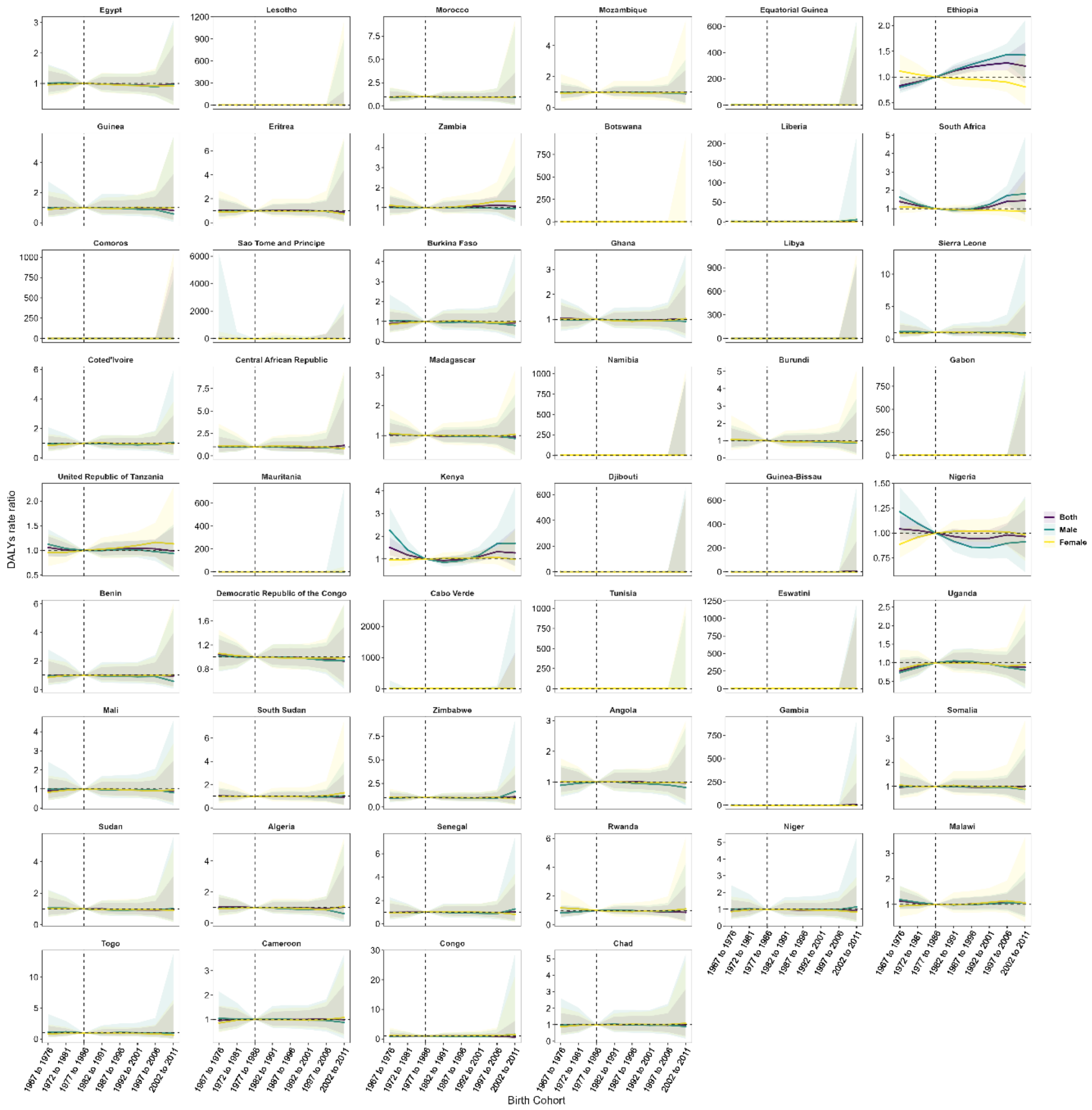

Figure S12 Cohort effects on substance use disorders attributable to childhood maltreatment in 52 African countries.
